# Integrative Analysis of ceRNA Network Reveals Functional lncRNAs in Intrahepatic Cholangiocarcinoma

**DOI:** 10.1155/2019/2601271

**Published:** 2019-11-25

**Authors:** Dongkai Zhou, Bingqiang Gao, Qifan Yang, Yang Kong, Weilin Wang

**Affiliations:** Department of Hepatobiliary & Pancreatic Surgery, The Second Affiliated Hospital, College of Medicine, Zhejiang University, No. 88 Jiefang Road, Hangzhou, Zhejiang, China

## Abstract

Intrahepatic cholangiocarcinoma (ICC) is the second most common lethal liver cancer worldwide. Currently, despite the latest developments in genomics and transcriptomics for ICC in recent years, the molecular pathogenesis promoting ICC remains elusive, especially in regulatory mechanisms of long noncoding RNAs (lncRNAs), which acts as competing endogenous RNA (ceRNA). In order to elucidate the molecular mechanism of functional lncRNA, expression profiles of lncRNAs, microRNAs (miRNAs), and messenger RNAs (mRNAs) were obtained from The Cancer Genome Atlas (TCGA) database and an integrative analysis of the ICC-associated ceRNA network was performed. Moreover, gene oncology enrichment analyses for the genes in the ceRNA network were implemented and novel prognostic biomarker lncRNA molecules were identified. In total, 6,738 differentially expressed mRNAs (DEmRNAs), 2,768 lncRNAs (DElncRNAs), and 173 miRNAs (DEmiRNAs) were identified in tumor tissues and adjacent nontumor ICC tissues with the thresholds of adjusted *P* < 0.01 and |logFC| > 2. An ICC-specific ceRNA network was successfully constructed with 30 miRNAs, 16 lncRNAs, and 80 mRNAs. Gene oncology enrichment analyses revealed that they were associated with the adaptive immune response, T cell selection and positive regulation of GTPase activity categories. Among the ceRNA networks, DElncRNAs ARHGEF26-AS1 and MIAT were found to be hub genes in underexpressed and overexpressed networks, respectively. Notably, univariate Cox regression analysis indicated that DElncRNAs HULC significantly correlated with overall survival (OS) in ICC patients (*P* value < 0.05), and an additional survival analysis for HULC was reconfirmed in an independent ICC cohort from the Gene Expression Omnibus (GEO) database. These findings contribute to a more comprehensive understanding of the ICC-specific ceRNA network and provide novel strategies for subsequent functional studies of lncRNAs in ICC.

## 1. Introduction

Intrahepatic cholangiocarcinoma (ICC) is the second most common lethal liver cancer worldwide [1]. Various risk factors have been confirmed to contribute to the progression of this disease, including sclerosing cholangitis, chronic hepatitis B or C viral (HBV, HCV) infection, and fibropolycystic liver disease, and so on [2]. Surgical resection, chemotherapy, and emerging immunotherapy are alternative options to cure patients with ICC. However, due to the high rate of recurrence in this tumor [3], none of these approaches can significantly prolong long-term survival. Currently, although latest advances in genomics and transcriptomics [4–6] have broadened our knowledge of ICC tremendously, the molecular pathogenesis promoting ICC remains elusive [7]. Therefore, there is a great need for understanding the specific molecular mechanism of tumors and for the identification of potential molecular biomarkers for ICC diagnosis and treatment.

In recent years, accumulating studies have focused on long noncoding RNAs (lncRNA) that are defined as transcripts over 200 nucleotides in length and have indicated that lncRNAs substantially affect gene expression that is dysregulated in numerous cancers. For instance, homeobox transcript antisense intergenic RNA (HOTAIR) was found to facilitate tumorigenesis by promoting phosphatase and tensin homolog (PTEN) methylation [8], and PVT1 binds EZH2 directly to silence *ANGPTL4* expression by promoting cell growth and migration in cholangiocarcinoma [9]. One of the most known mechanisms for lncRNA is that it works as a competing endogenous RNA (ceRNA). This hypothesis was first proposed by Salmena et al. [10], which holds that lncRNA is involved in posttranscriptional regulation by functioning as sponges to modulate both miRNAs and mRNA expressional levels. In addition, emerging studies have confirmed that lncRNA acts as a hub gene in the regulation network between miRNA and target genes that are involved in a variety of cellular biological processes, especially in tumor proliferation and metastasis [11]. However, little is known about the comprehensive landscape of the ICC-associated ceRNA regulatory network, and very few studies were performed to investigate the lncRNAs mechanism for ICC.

In the present study, in order to elucidate the molecular mechanism of lncRNA and the lncRNA-mediated regulatory network, we performed an integrative analysis of identifying differentially expressed mRNAs (DEmRNAs), lncRNAs (DElncRNAs), and miRNA (DEmiRNAs) of ICC using data from The Cancer Genome Atlas (TCGA) database. Next, an ICC-associated ceRNA network was successfully constructed and the overall survival (OS) analyses were carried out to identify molecules that were novel prognostic biomarkers and potential targets for ICC [12]. This study contributes to the exploration of the ceRNA regulatory mechanism of ICC and provides valuable insight for further functional research.

## 2. Materials and Methods

### 2.1. Study Population

Patients and samples of RNAseq associated with ICC were downloaded from the Genomic Data Commons (GDC) database (https://portal.gdc.cancer.gov/, Data Release version 16.0, release time: March 26, 2019). In total, 33 tumors and 9 adjacent nontumor tissues of ICC were included in our study. Lack of complete clinical data was considered as the exclusion criteria. The RNA and miRNA expression profiles and clinical follow-up data of the ICC cohort were downloaded from the TCGA database. This study was conducted in accordance with the publication guidelines of TCGA (http://cancergenome.nih.gov/publications/publicationguidelines). In addition, the lncRNA expression profiles from GSE89749 (including 81 ICC samples) were used as an independent validation set.

### 2.2. Differentially Expressed Analysis

The raw read counts (lncRNA, miRNA, and mRNA) were first normalized by using the trimmed mean of *M*-values (TMM) method in the “edgeR” package of R software (Version: 3.4.3). The expression levels of lncRNA, miRNA, and mRNAs in ICC were calculated as the log 2(*x*+1) of the TMM normalized level. To identify DElncRNAs, DEmiRNAs, and DEmRNAs, we compared the nontumor tissues with the ICC tumors by using the “edgeR” package [13] with a significance threshold of an absolute log2 fold change ≥2 and a false discovery rate- (FDR-) adjusted *P* value less than 0.05. In addition, a volcano plot was made by using the ggplot2 package to identify the differential expression genes (DEGs) with statistical significance between the tumor and nontumor groups. Hierarchical clustering and heatmap plot analyses were performed for the DEGs of DElncRNAs, DEmiRNAs, and DEmRNAs by using ComplexHeatmap [14] package in R.

### 2.3. Collection of mRNA-lncRNA-miRNA Interactions Data

The lncRNA-miRNA interactions data were predicted from the miRcode database (http://www.mircode.org/) and the starBase v3.0 (http://starbase.sysu.edu.cn/). The lncRNA-miRNA interactions were then obtained according to the intersection of the 2 databases. In addition, miRNA-mRNA interactions data were predicted using miRcode (http://www.mircode.org/), miRDB (http://www.mirdb.org/), miRanda (http://www.microrna.org/microrna/home.do), miRTarBase (http://mirtarbase.mbc.nctu.edu.tw/), and TargetScan database (http://www.targetscan.org/). The intersection of the lncRNA-miRNA interaction pairs predicted by at least 3 of these databases was considered as the final lncRNA-miRNA interactions.

### 2.4. Constructing the ceRNA Network Analysis

To investigate the potential interactions between lncRNAs and mRNAs, we constructed the ceRNA coregulated network using DElncRNAs, DEmiRNAs, and DEmRNAs. The coexpression analysis was performed by using Pearson's correlation coefficient (PCC) to identify correlation pairs according to the expression levels between significantly dysregulated DElncRNAs and DEmRNAs. The Pearson correlation cutoff value >0.7 or <−0.7 with *P* < 0.001 was defined as the screening threshold for retaining the RNAs in the analysis. Finally, the coregulated network was constructed using Cytoscape (version 3.7.0) [15] to illustrate the ceRNA network.

### 2.5. GO and KEGG Functional Enrichment Analysis of DEGs

The GO [16] database stores extensive information of gene sets including GO terms and the annotations of genes and provides informative pathways for substantial genes. The GO function and KEGG pathway involved in DEGs were analyzed by the online tool DAVID [17] (version: 6.7, https://david-d.ncifcrf.gov/summary.jsp). The terms with the number of enriched genes counts ≥2 and the hypergeometric significance *P* values <0.05 were considered significant.

### 2.6. Protein-Protein Interaction (PPI) Network Submodule Analysis

In the PPI network, proteins with similar functions tend to cluster. Therefore, the analysis of the functional clustering module in the PPI network may help us understand the unknown functions of proteins. In this study, the Molecular Complex Detection (MCODE) plugin in Cytoscape [18], a recursive vertex weighting scoring scheme based algorithm, was used to analyze the subnetwork module in the PPI network with a module score >0.5. By using the MCODE plugin of Cytoscape (Version: 1.4.2), the submodule of PPI network was analyzed with the default thresholds: Degree Cutoff: 2, Node Score Cutoff: 0.2, K-Core: 2, and Max Depth: 100. GO and KEGG pathway enrichment analyses for the DEGs involved in the module were then performed.

### 2.7. Prognostic Analysis

In order to determine the association between DEmRNAs, DElncRNAs, and DEmiRNAs in the ceRNA network and prognostic OS in ICC, univariate Cox proportional hazards regression method was performed to analyze the relationship between the DElncRNAs and OS at a significant level of 0.05.

### 2.8. Validation of Prognostic lncRNA in an Independent Data Set

To assess the prognostic lncRNA in another independent data set, lncRNA expression profiling of 81 ICC patients, based on Illumina HumanHT-12 V4.0 Expression Beadchip, was downloaded from the NCBI database of Gene Expression Omnibus (GEO) accession GSE89749. Optimal cutoff value was achieved by the function of surv_cutpoint in “survminer” R package. Kaplan–Meier plot was used to depict the survival curves and *P* < 0.05 was considered as significant.

### 2.9. Statistical Analysis

Wilcoxon test was used for comparison between the two groups with R (Version: 3.4.3) software. Statistical significance was set at *P* < 0.05.

## 3. Results

### 3.1. Identification of DEmRNAs

**Figure 1:**
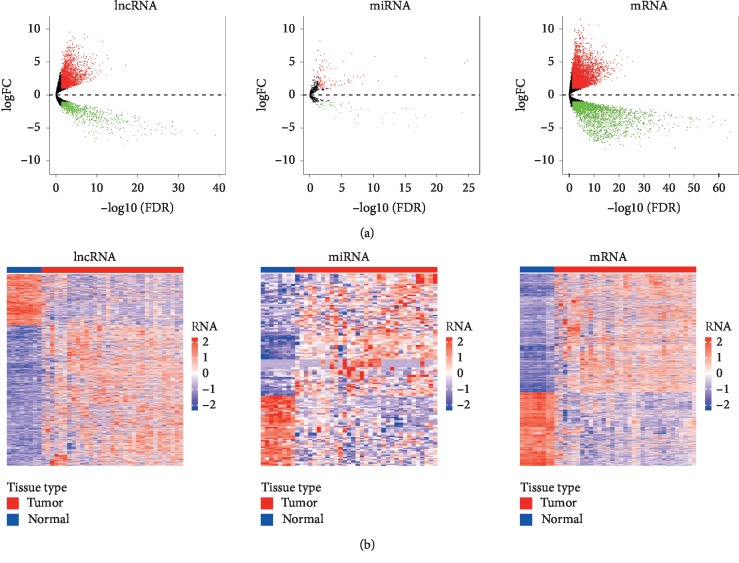
(a) Volcano plots showing the differential expression of lncRNAs, miRNAs, and mRNAs between intrahepatic cholangiocarcinoma (ICC) and adjacent normal tissues (|log 2FoldChange| > 2.0 and adjusted *P* value (FDR) < 0.01). The *x*-axis represents log2 transformed false discovery rate (FDR) values plotted in −log10, and the *y*-axis depicts the value of log2 transformed fold change in gene expression. Red dots represent upregulation while green dots represent downregulation. (b) Heatmaps of the differential expressed lncRNAs, miRNAs, and mRNAs between ICC and adjacent normal samples. Differential expressed genes are represented in rows, and samples are represented in columns. The expression value for each row was normalized by the *z*-score. Red stripes indicate high expression and blue stripes indicate low expression of genes. Blue bar represents normal samples, while red one represents samples.

We identified a total of 6738 DEmRNAs, 2768 DElncRNAs, and 173 DEmiRNAs in tumor tissues and adjacent nontumor ICC tissues using the “edgeR” package with the thresholds of *P* < 0.01 and |log  FC| > 2. We found 4164 (61.80%) upregulated and 2574 (38.20%) downregulated DEmRNAs, 2028 (73.27%) upregulated and 740 (26.73%) downregulated DElncRNAs, and 110 (63.58%) upregulated and 63 (36.42%) downregulated DEmiRNAs between ICC and normal samples (Figure 1(a)). In addition, volcano plots were generated to demonstrate the distribution of the DElncRNAs, DEmiRNAs, and DEmRNAs, and the heatmap with clustering of DEmRNAs was illustrated using the “ggplots” package in R software (Figure 1(b)).

### 3.2. Prediction of Target miRNAs of DElncRNAs and Target mRNAs of miRNAs

Based on the hypothesis that miRNAs interact with the lncRNAs through MREs, data of miRNAs targeting lncRNAs were from the miRcode and starBase v3.0 database. A total of 273 miRNAs that putatively target 10,349 lncRNAs, including 476,957 DElncRNA-DEmiRNA interactions, were obtained. Subsequently, we obtained the intersection of lncRNA-miRNA interactions by screening DElncRNAs and DEmiRNAs. Finally, 42 miRNAs and 196 lncRNAs were identified including 2132 DElncRNA-DEmiRNA interactions ([Supplementary-material supplementary-material-1]). Moreover, 173 identified DEmiRNAs were mapped into the mircode, miRDB, miRanda, miRTarBase, and TargetScan databases to predict their target mRNAs. Then, the intersection pairs of the DEmRNAs to miRNAs predicted by at least 3 three databases were considered as target DEmRNAs of DEmiRNAs. We obtained 2068 DEmRNAs targeted by 43 DEmiRNAs with 4623 DEmRNAs-DEmiRNAs pairs ([Supplementary-material supplementary-material-1]).

### 3.3. Establishment of the ceRNA Network in ICCs and Functional Enrichment Analysis

**Figure 2:**
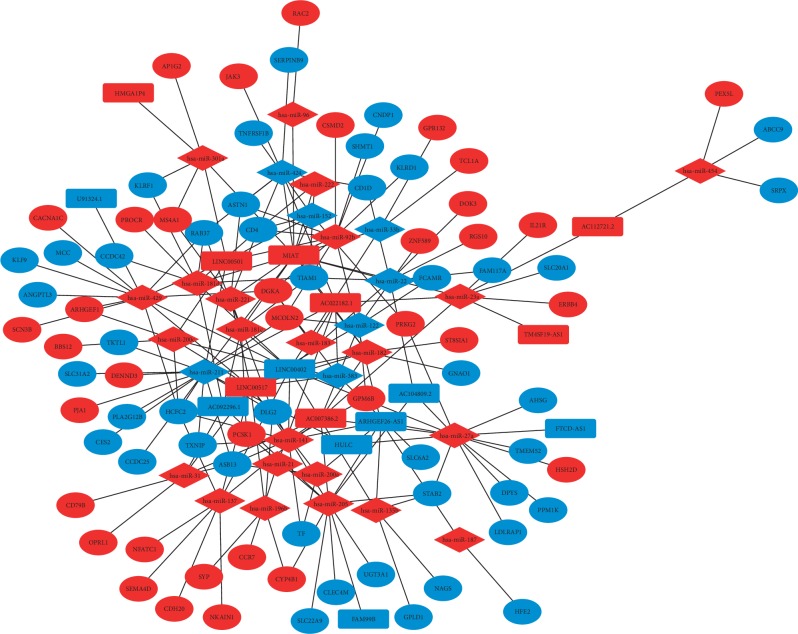
The regulatory network of the DEmRNAs-DEmiRNAs-DElncRNAs in ICC. DEmRNAs, DEmiRNAs, and DElncRNAs are indicated by the ellipse, rectangle, and diamond shape, respectively. The color red represents overexpression, and blue represents underexpression.

**Figure 3:**
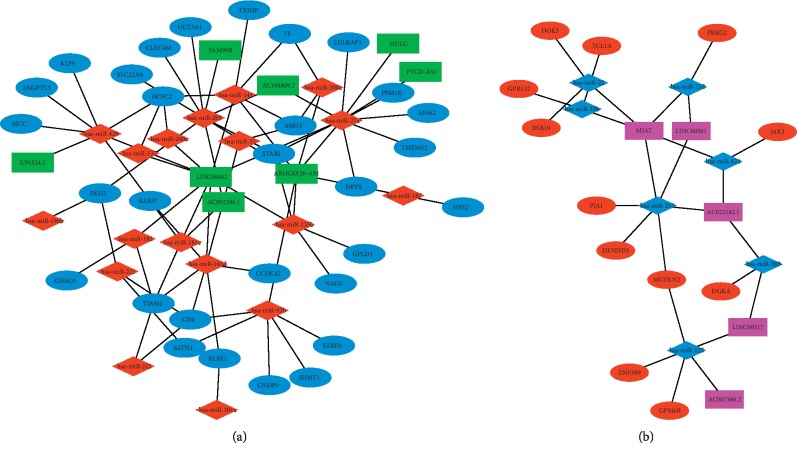
The lncRNA-miRNA-mRNA competing endogenous RNA (ceRNA) of underexpressed (a) and overexpressed (b) lncRNAs-mRNAs networks in ICC. In network (a), blue ellipse represents downregulated mRNAs; green rectangle, downregulated lncRNAs; and red diamond, upregulated miRNAs. In network (b), red ellipse represents upregulated mRNAs; pink rectangle, upregulated lncRNAs; and blue diamond, downregulated miRNAs.

To better understand the pivotal roles of the DElncRNA, DEmiRNA, and DEmRNA in ICC, the ceRNA network was constructed on the hypothesis that lncRNAs are involved in posttranscriptional regulation by working as sponges to directly modulate both miRNAs and mRNA expressional levels. Based on this hypothesis, a total of 122 lncRNA-miRNA-mRNA interaction pairs were identified in the proposed ceRNA network, including 30 miRNAs (23 upregulated and 7 downregulated, [Supplementary-material supplementary-material-1]), 16 lncRNAs (8 upregulated and 8 downregulated, [Supplementary-material supplementary-material-1]), and 80 mRNAs (36 upregulated and 44 downregulated, [Supplementary-material supplementary-material-1]). The whole lncRNA-miRNA-mRNA ceRNA regulatory network was visualized by Cytoscape (version 3.7.0) (Figure 2). In order to interrogate the biological functions of DEmRNAs in the ceRNA network, pathway enrichment analysis was applied to these DEmRNAs. We observed that most of the enriched GO pathways were associated with the adaptive immune response (*P*=0.0041), T cell selection (*P*=0.013), and positive regulation of GTPase activity (*P*=0.038) categories. The detailed results of the GO analysis are shown in [Supplementary-material supplementary-material-1]. In addition, in order to systematically investigate the effects of lncRNA-mediated regulation on the ceRNA network, 2 underexpressed (Figure 3(a)) and overexpressed networks (Figure 3(b)) were reconstructed. In the underexpressed network, ARHGEF26-AS1 was the important hub regulator in the ceRNA network. ARHGEF26-AS1 interacted with 7 DEmiRNAs (hsa-miR-135b, hsa-miR-141, hsa-miR-187, hsa-miR-200a, hsa-miR-205, hsa-miR-27a, and hsa-miR-92b) and was coregulated with 30 DEmRNAs (Figure 3(a)). In the overexpressed network, MIAT was the important hub regulator, which interacted with 14 DEmiRNAs (hsa-miR-152, hsa-miR-181c, hsa-miR-181d, hsa-miR-182, hsa-miR-211, hsa-miR-22, hsa-miR-221, hsa-miR-222, hsa-miR-23a, hsa-miR-301a, hsa-miR-33b, hsa-miR-424, hsa-miR-92b, and hsa-miR-96) and was coregulated with 43 DEmRNAs (Figure 3(b)).

### 3.4. PPI Network Construction and Pathway Enrichment Analysis of Subnetworks

**Figure 4:**
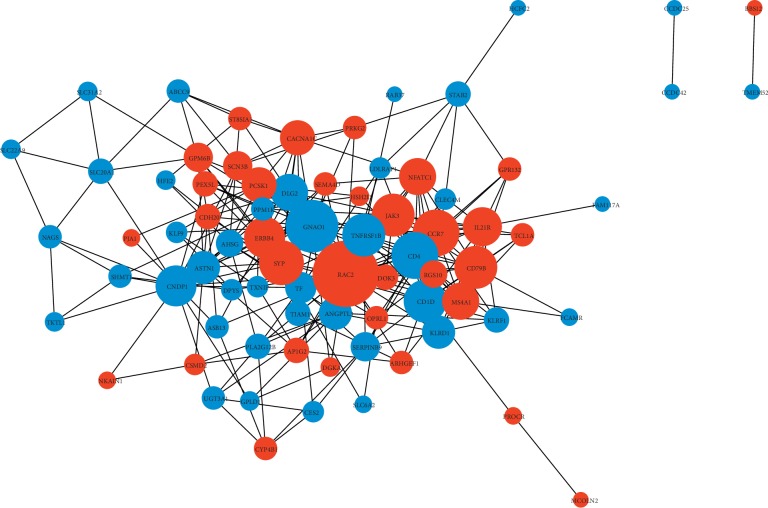
Protein-protein interaction (PPI) network of hub genes of ceRNA in ICC. PPI data obtained from the online database STRING v11 and PPI network was constructed with scores of >0.4. The gray line indicates the interaction of the mRNAs. Red represents high expression and blue represents low expression. The size of the circle represents the degree of connection. The larger circles are hub genes in the network.

To better understand the hub genes in ICC, we established a PPI network that harbored 78 genes according to the information from the STRING database v11 with scores of >0.4 (Figure 4). From Figure 4, we determined that the key hub nodes were RAC2, SYP, ERBB4, GNAO1, TNFRSF1B, CCR7, and CD4. Through MCODE analysis (score > 5), 4 subnetworks consisting of 42 nodes and 120 edges ([Supplementary-material supplementary-material-1]) were found. Based on the enrichment analysis for each subnetwork, we observed that subnetwork 1 was strongly associated with the chemokine signaling pathway (*P*=0.0046), B cell receptor signaling pathway (*P*=0.0072), and natural killer cell-mediated cytotoxicity (*P*=0.021). Subnetwork 2 was mainly involved in renin secretion (*P*=0.037) and GABAergic synapse (*P*=0.0049), and subnetwork 3 was mainly involved in the biosynthesis of amino acids (*P*=0.0003), metabolic pathways (*P*=0.0056), and carbon metabolism (*P*=0.0048). Only subnetwork 4 had a significant association with KEGG pathways ([Supplementary-material supplementary-material-1]).

### 3.5. Survival Analysis for RNAs in ICC ceRNA Network

**Figure 5:**
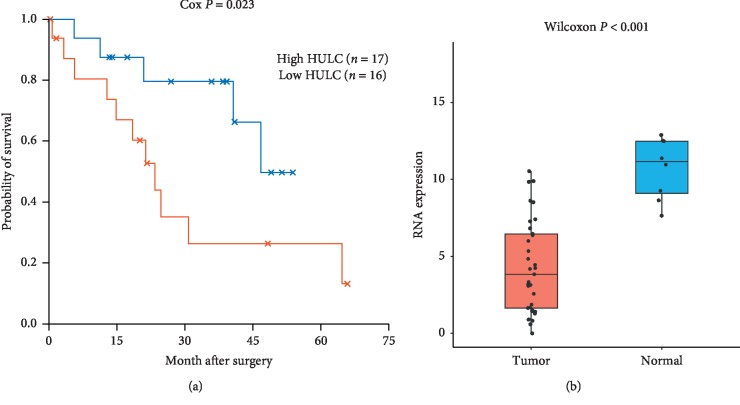
(a) Kaplan–Meier survival curves for a DElncRNA associated with the overall survival (OS) in ICC patients. Cox *P* value was used to assess the survival differences between the two groups. (b) Box-plot for the RNA expression of lncRNA (HULC) between ICC and normal tissues. The *y*-axis represents the relative gene expressions (TMM normalized and log 2(*x*+1) transformed) and the *x*-axis represents the tumor or normal tissues.

To investigate the OS for these hub genes of DElncRNAs, DEmiRNAs, and DEmRNAs in the ceRNA network, we performed Cox proportional hazard analysis to predict OS in ICC patients. Notably, only 1 DElncRNAs HULC was correlated with the prognosis of patients with ICC. We observed that lncRNA HULC had low expression in tumor tissues compared to nontumor tissues but the high expression of HULC was associated with poor prognosis with a Cox *P* value of 0.023, HR = 1.22, 95% CI 1.02–1.47 (Figures 5(a) and 5(b)). Moreover, lncRNA of HULC was reconfirmed to be significantly associated with OS (*P*=0.013) in another independent ICC cohort from GEO accession GSE89749 ([Supplementary-material supplementary-material-1]). In addition, several lncRNA-miRNA-mRNA interactions were screened in the ceRNA network such as “HULC-mir-27a-LDLRAP1,” “HULC-mir-27a-PPM1K,” “HULC-mir-27a-AHSG,” “HULC-mir-27a-DPYS,” “HULC-mir-27a-STAB2,” and “HULC-mir-27a-TMEM52” (Figure 3(a)).

## 4. Discussion

Intrahepatic cholangiocarcinoma (ICC) is a serious disease affecting millions of individuals worldwide; however, the molecular pathogenesis promoting ICC remains unexplained. To date, numerous studies have been performed to reveal the underlying mechanisms of lncRNAs in regulating the development and progression of ICC, but these studies only focus on a single genetic event and may not satisfy the current requirement on discovering potential molecular targets for treating ICC. To better understand the role of lncRNA on the ceRNA network of ICC, we systematically integrated transcriptome expression profiles from TCGA to construct a lncRNA-associated ceRNA network to determine the regulatory mechanisms of lncRNAs. Moreover, we screened hub genes to identify novel prognostic biomarkers and potential treatment targets in the ceRNA network for ICC.

In total, 6,738 DEmRNAs, 2,768 DElncRNAs, and 173 DEmiRNAs were identified in ICC tumors compared to nontumor tissues. We further constructed an ICC-specific network, and in order to make the results more reliable and stable, we used miRcode and starBase databases to predict target lncRNAs of DEmiRNAs and used mircode, miRDB, miRanda, miRTarBase, and TargetScan databases to predict target mRNAs of DEmiRNAs. We constructed an ICC-specific ceRNA network based on a total of 122 lncRNA-miRNA-mRNA interaction pairs, including 30 miRNAs, 16 lncRNAs, and 80 mRNAs. Pathway enrichment analysis was performed on the DEmRNAs in the ceRNA network and revealed that most of the enriched GO pathways were associated with the adaptive immune response, T cell selection, and positive regulation of GTPase activity categories. Subsequently, 2 underexpressed and overexpressed networks were reconstructed to investigate the effects of lncRNA-mediated regulation on the ceRNA network. Notably, 2 hub regulators were highlighted in the ICC-specific ceRNA network, one being ARHGEF26-AS1, which interacted with 7 DEmiRNAs and was coregulated with 30 DEmRNAs. ARHGEF26-AS1 (ARHGEF26 antisense RNA 1) is an antisense RNA of ARHGEF26, and it has been reported that ARHGEF26-AS1 was significantly correlated with OS (*P* value < 0.05) in colon cancer [19]. However, no experimental evidence was found to support the biological function of ARHGEF26-AS1 in recent studies. MIAT was another important hub regulator, which interacted with 14 DEmiRNAs and was coregulated with 43 DEmRNAs. Many studies revealed that MIAT has been demonstrated as a key switcher in regulation of various biological and pathological processes in human cancers including non-small-cell lung cancer, neuroendocrine prostate cancer, and colorectal cancer [20–22]. The aberrant upregulation expression of MIAT in human malignancies can promote tumorigenesis through lncRNA-miRNA-mRNA networks. For example, it was reported that MIAT regulates cell proliferation, migration, and invasion in gastric cancer through a mechanism involving the miR-29a-3p/HDAC4 axis [23]. Moreover, a PPI network harboring 78 hub genes was constructed, and according to the MCODE analysis, we finally obtained 4 modules consisting of 42 nodes and 120 edges ([Supplementary-material supplementary-material-1]). Enrichment analysis for the subnetworks discovered that subnetwork 1 was strongly associated with the chemokine signaling pathway, cell receptor signaling pathway, and natural killer cell-mediated cytotoxicity. Subnetwork 2 was involved in renin secretion and GABAergic synapse. Subnetwork 3 was involved in the biosynthesis of amino acids, metabolic pathways, and carbon metabolism. Except for Subnetwork 4, no significantly associated KEGG pathways were observed. These results suggest that lncRNA-associated ceRNA subnetworks may disrupt cancer immune and metabolic pathways in ICC.

To further investigate OS for these hub genes of DElncRNAs, DEmiRNAs, and DEmRNAs in the ceRNA network, Cox's proportional hazards analysis revealed that DElncRNA HULC was associated with the prognosis of patients with ICC (*P* value < 0.05). lncRNA HULC was found to have a lower expression level in tumors compared to nontumor tissues but the high expression of HULC in tumors was associated with poor prognosis of ICC. HULC functions as an oncogene in human cancer which promotes tumorigenesis by regulating multiple signaling pathways [24]. Especially in hepatocellular carcinoma (HCC), accumulating studies have been reported that lncRNA HULC has a potential as a promising therapeutic target in HCC and it can accelerate liver cancer by inhibiting PTEN via autophagy cooperation to miR15a [25–27]. However, to date, no studies have been reported about the association of HULC with ICC. With this study, we have shown that the aberrant upregulated expression of HULC affects OS in ICC and indicates a potential prognostic biomarker in ICC.

Our findings indicate that the lncRNA-associated ceRNA network plays pivotal roles in tumorigenesis and progression of ICC. Although our study provides comprehensive analysis of the competitive ceRNAs network and functional lncRNAs of ICC, several limitations of our research need to be noted. Firstly, the relationship between the ceRNA network and clinical features of ICC was not well addressed in our study due to incomplete clinical information. Secondly, the cohorts of ICC in the public databases were not large enough because ICC itself is a rare tumor and only accounts for about 3% of all gastrointestinal cancers [28], which might bias the results. Thirdly, additional experiments are needed to verify our results on whether ceRNA networks and functional lncRNAs are functional or specific for ICC rather than other cancer types.

## 5. Conclusions

Our study has identified several novel prognostic factors and potential treatment targets for ICC by analyzing RNA expression profiling data from the TCGA database. Our data provide a comprehensive understanding of the lncRNA-related ceRNA network in ICC but further functional experiments are needed to elucidate the underlying mechanisms.

## Data Availability

The data used to support the findings of this study are included within the article. Moreover, RNA and miRNA expression profiles and clinical follow-up data are obtained from a public database of TCGA.

## Supplementary Materials

Supplementary Materials Figure S1: the ceRNA subnetworks of protein-protein interaction (PPI) network. Figure S2: Kaplan–Meier survival curves for lncRNAs HULC associated with overall survival of 81 ICC patients from GSE89749. Table S1: details of DElncRNAs-DEmiRNAs pairs. Table S2: details of DEmRNAs-DEmiRNAs pairs. Table S3: details of upregulated and downregulated miRNAs in ceRNA network. Table S4: details of upregulated and downregulated lncRNAs in ceRNA network. Table S5: details of upregulated and downregulated mRNAs in ceRNA network. Table S6: GO pathway enrichment of mRNAs in ceRNA network. Table S7: KEGG pathway enrichment of subnetworks in ceRNA network.
