# Supplementary material for: Integrative Analysis of ceRNA Network Reveals Functional lncRNAs in Intrahepatic Cholangiocarcinoma
Source: Biomed Res Int. 2019 Nov 25;2019:2601271. doi: 10.1155/2019/2601271 (PMC6899321; doi:10.1155/2019/2601271)
Supplement: Supplementary Materials — Figure S1: the ceRNA subnetworks of protein-protein interaction (PPI) network. Figure S2: Kaplan–Meier survival curves for lncRNAs HULC associated with overall survival of 81 ICC patients from GSE89749. Table S1: details of DElncRNAs-DEmiRNAs pairs. Table S2: details of DEmRNAs-DEmiRNAs pairs. Table S3: details of upregulated and downregulated miRNAs in ceRNA network. Table S4: details of upregulated and downregulated lncRNAs in ceRNA network. Table S5: details of upregulated and downregulated mRNAs in ceRNA network. Table S6: GO pathway enrichment of mRNAs in ceRNA network. Table S7: KEGG pathway enrichment of subnetworks in ceRNA network. [file 2601271.f1.docx]

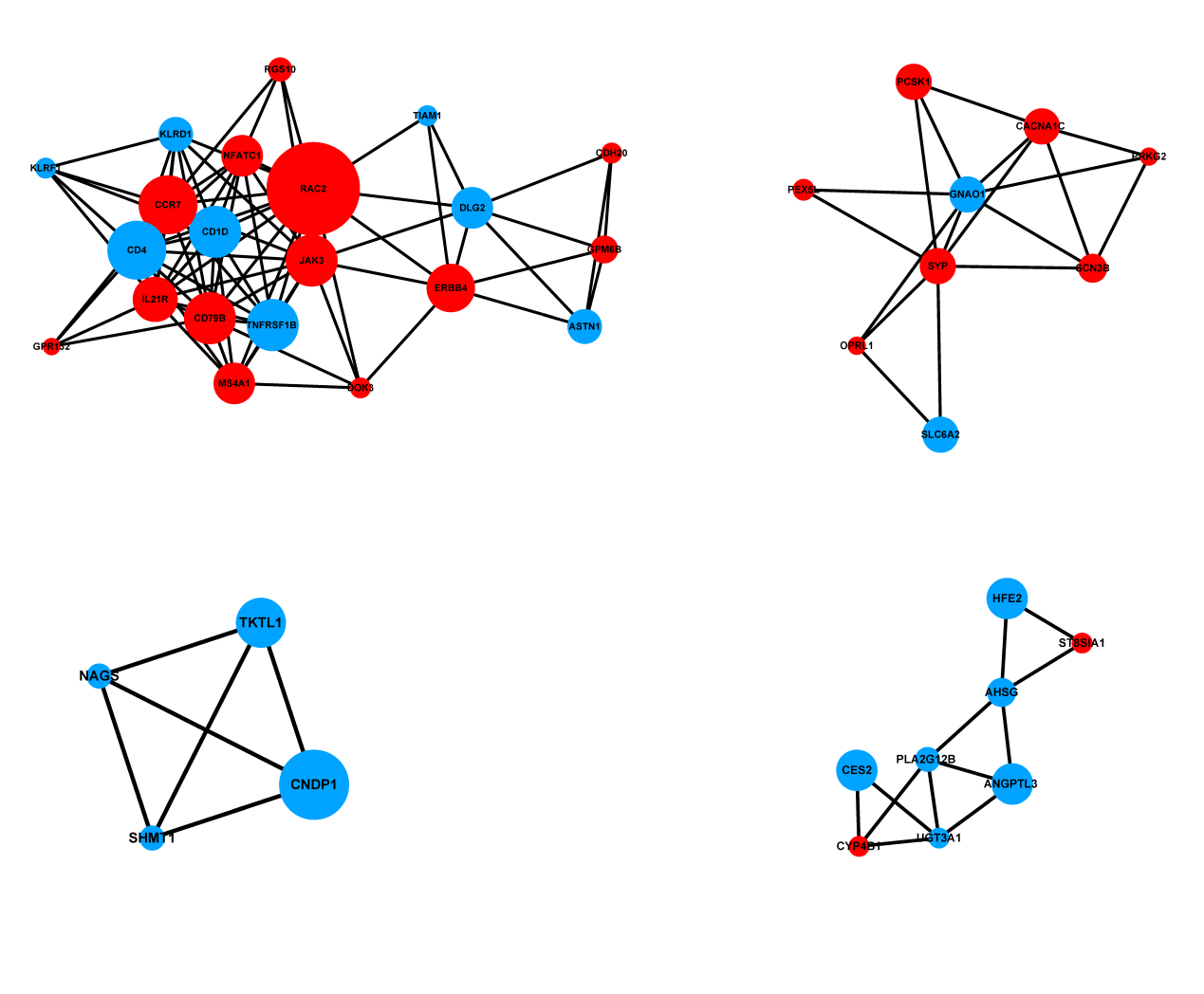


**Figure S1. The ceRNA subnetworks of protein-protein interaction (PPI) network in ICC.** Four subnetworks of PPI data were obtained from the Molecular Complex Detection (MCODE) plug-ins in the Cytoscape software v3.7.0. The dark line indicates the interaction of the mRNAs. Red represents high expression and blue represents low expression. The size of the circle represents the degree of connection. The larger circles are hub genes in the network.


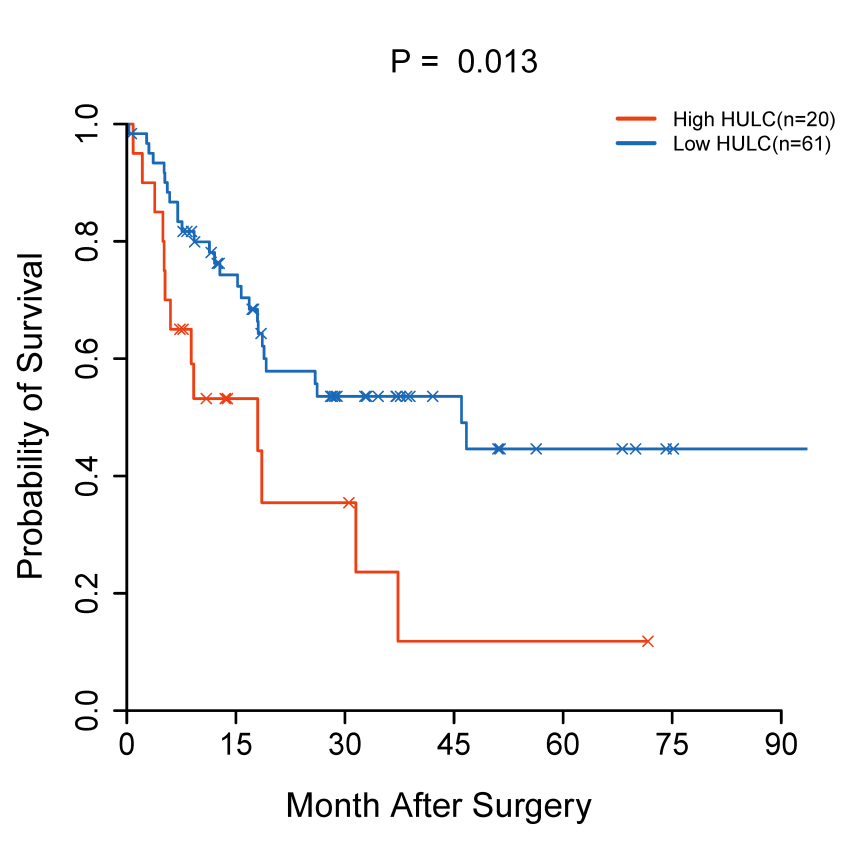


**Figure S2. Kaplan-Meier survival curves for lncRNAs HULC associated with overall survival of 81 ICC patients from GSE89749.** Kaplan-Meier survival curves for lncRNAs HULC associated with overall survival based on their optimal cutoffs in ICC. The horizontal axis represents the overall survival time in months; while the vertical axis represents the probability of survival.

| **Table S1. Details of DElncRNAs-DEmiRNAs pairs.** | |
| --- | --- |
| miRNA | lncRNA |
| hsa-miR-301a | AC062028.1.1 |
| hsa-miR-454 | AC062028.1.1 |
| hsa-miR-96 | AC062028.1.1 |
| hsa-miR-96 | AC062028.1.1 |
| hsa-miR-182 | AC062028.1.1 |
| hsa-miR-211 | AC062028.1.1 |
| hsa-miR-92b | AC062028.1.1 |
| hsa-miR-27a | AC062028.1.1 |
| hsa-miR-31 | AC062028.1.1 |
| hsa-miR-338 | AC062028.1.1 |
| hsa-miR-33b | AC062028.1.1 |
| hsa-miR-34c | AC062028.1.1 |
| hsa-miR-10b | AC062028.1.1 |
| hsa-miR-135b | AC007364.1.1 |
| hsa-miR-137 | AC007364.1.1 |
| hsa-miR-141 | AC007364.1.1 |
| hsa-miR-200a | AC007364.1.1 |
| hsa-miR-155 | AC007364.1.1 |
| hsa-miR-192 | AC007364.1.1 |
| hsa-miR-211 | AC007364.1.1 |
| hsa-miR-211 | AC007364.1.1 |
| hsa-miR-211 | AC007364.1.1 |
| hsa-miR-205 | AC007364.1.1 |
| hsa-miR-383 | AC007364.1.1 |
| hsa-miR-135b | AC017048.3.1 |
| hsa-miR-135b | AC017048.3.1 |
| hsa-miR-192 | AC017048.3.1 |
| hsa-miR-122 | AC017048.3.1 |
| hsa-miR-31 | AC017048.3.1 |
| hsa-miR-34c | AC017048.3.1 |
| hsa-miR-96 | LINC00483 |
| hsa-miR-144 | LINC00483 |
| hsa-miR-144 | LINC00483 |
| hsa-miR-182 | LINC00483 |
| hsa-miR-182 | LINC00483 |
| hsa-miR-183 | LINC00483 |
| hsa-miR-192 | LINC00483 |
| hsa-miR-192 | LINC00483 |
| hsa-miR-21 | LINC00483 |
| hsa-miR-122 | LINC00483 |
| hsa-miR-122 | LINC00483 |
| hsa-miR-122 | LINC00483 |
| hsa-miR-27a | LINC00483 |
| hsa-miR-27a | LINC00483 |
| hsa-miR-27a | LINC00483 |
| hsa-miR-30a | LINC00483 |
| hsa-miR-30b | LINC00483 |
| hsa-miR-30d | LINC00483 |
| hsa-miR-31 | LINC00483 |
| hsa-miR-33b | LINC00483 |
| hsa-miR-211 | GRIK1-AS1 |
| hsa-miR-205 | GRIK1-AS1 |
| hsa-miR-221 | GRIK1-AS1 |
| hsa-miR-222 | GRIK1-AS1 |
| hsa-miR-338 | GRIK1-AS1 |
| hsa-miR-338 | GRIK1-AS1 |
| hsa-miR-383 | GRIK1-AS1 |
| hsa-miR-301a | CASC2 |
| hsa-miR-454 | CASC2 |
| hsa-miR-135b | CASC2 |
| hsa-miR-135b | CASC2 |
| hsa-miR-141 | CASC2 |
| hsa-miR-200a | CASC2 |
| hsa-miR-144 | CASC2 |
| hsa-miR-144 | CASC2 |
| hsa-miR-144 | CASC2 |
| hsa-miR-144 | CASC2 |
| hsa-miR-152 | CASC2 |
| hsa-miR-155 | CASC2 |
| hsa-miR-155 | CASC2 |
| hsa-miR-155 | CASC2 |
| hsa-miR-155 | CASC2 |
| hsa-miR-155 | CASC2 |
| hsa-miR-424 | CASC2 |
| hsa-miR-181c | CASC2 |
| hsa-miR-181d | CASC2 |
| hsa-miR-183 | CASC2 |
| hsa-miR-192 | CASC2 |
| hsa-miR-192 | CASC2 |
| hsa-miR-200b | CASC2 |
| hsa-miR-200c | CASC2 |
| hsa-miR-429 | CASC2 |
| hsa-miR-211 | CASC2 |
| hsa-miR-205 | CASC2 |
| hsa-miR-205 | CASC2 |
| hsa-miR-205 | CASC2 |
| hsa-miR-21 | CASC2 |
| hsa-miR-21 | CASC2 |
| hsa-miR-21 | CASC2 |
| hsa-miR-221 | CASC2 |
| hsa-miR-222 | CASC2 |
| hsa-miR-221 | CASC2 |
| hsa-miR-222 | CASC2 |
| hsa-miR-23a | CASC2 |
| hsa-miR-23a | CASC2 |
| hsa-miR-92b | CASC2 |
| hsa-miR-27a | CASC2 |
| hsa-miR-31 | CASC2 |
| hsa-miR-31 | CASC2 |
| hsa-miR-31 | CASC2 |
| hsa-miR-338 | CASC2 |
| hsa-miR-33b | CASC2 |
| hsa-miR-33b | CASC2 |
| hsa-miR-34c | CASC2 |
| hsa-miR-383 | CASC2 |
| hsa-miR-383 | CASC2 |
| hsa-miR-135b | LINC00304 |
| hsa-miR-152 | LINC00304 |
| hsa-miR-155 | LINC00304 |
| hsa-miR-183 | LINC00304 |
| hsa-miR-187 | LINC00304 |
| hsa-miR-221 | LINC00304 |
| hsa-miR-222 | LINC00304 |
| hsa-miR-30a | LINC00304 |
| hsa-miR-30b | LINC00304 |
| hsa-miR-30d | LINC00304 |
| hsa-miR-338 | LINC00304 |
| hsa-miR-10b | LINC00304 |
| hsa-miR-96 | TP53TG1 |
| hsa-miR-182 | TP53TG1 |
| hsa-miR-338 | TP53TG1 |
| hsa-miR-33b | TP53TG1 |
| hsa-miR-34c | TP53TG1 |
| hsa-miR-34c | LINC00334 |
| hsa-miR-187 | COL18A1-AS1 |
| hsa-miR-196b | COL18A1-AS1 |
| hsa-miR-205 | COL18A1-AS1 |
| hsa-miR-22 | COL18A1-AS1 |
| hsa-miR-30a | COL18A1-AS1 |
| hsa-miR-30b | COL18A1-AS1 |
| hsa-miR-30d | COL18A1-AS1 |
| hsa-miR-338 | COL18A1-AS1 |
| hsa-miR-34c | COL18A1-AS1 |
| hsa-miR-34c | COL18A1-AS1 |
| hsa-miR-135b | LINC00315 |
| hsa-miR-424 | LINC00315 |
| hsa-miR-183 | LINC00315 |
| hsa-miR-22 | LINC00315 |
| hsa-miR-22 | LINC00315 |
| hsa-miR-27a | LINC00315 |
| hsa-miR-34c | LINC00315 |
| hsa-miR-424 | LINC00313 |
| hsa-miR-424 | LINC00313 |
| hsa-miR-424 | LINC00313 |
| hsa-miR-187 | LINC00313 |
| hsa-miR-211 | LINC00313 |
| hsa-miR-205 | LINC00313 |
| hsa-miR-122 | LINC00313 |
| hsa-miR-31 | LINC00313 |
| hsa-miR-338 | LINC00313 |
| hsa-miR-10b | LINC00313 |
| hsa-miR-181c | LINC00158 |
| hsa-miR-181d | LINC00158 |
| hsa-miR-183 | LINC00158 |
| hsa-miR-211 | LINC00158 |
| hsa-miR-221 | LINC00158 |
| hsa-miR-222 | LINC00158 |
| hsa-miR-23a | LINC00158 |
| hsa-miR-22 | AC092171.2.1 |
| hsa-miR-135b | AC010969.1.1 |
| hsa-miR-152 | AC010969.1.1 |
| hsa-miR-211 | AC010969.1.1 |
| hsa-miR-22 | AC010969.1.1 |
| hsa-miR-22 | AC010969.1.1 |
| hsa-miR-30a | AC010969.1.1 |
| hsa-miR-30b | AC010969.1.1 |
| hsa-miR-30d | AC010969.1.1 |
| hsa-miR-34c | AC010969.1.1 |
| hsa-miR-96 | LINC00336 |
| hsa-miR-99a | LINC00336 |
| hsa-miR-99b | LINC00336 |
| hsa-miR-21 | LINC00336 |
| hsa-miR-23a | LINC00336 |
| hsa-miR-23a | LINC00336 |
| hsa-miR-27a | LINC00336 |
| hsa-miR-152 | SNHG12 |
| hsa-miR-424 | SNHG12 |
| hsa-miR-181c | SNHG12 |
| hsa-miR-181d | SNHG12 |
| hsa-miR-187 | SNHG12 |
| hsa-miR-200b | SNHG12 |
| hsa-miR-200c | SNHG12 |
| hsa-miR-429 | SNHG12 |
| hsa-miR-211 | SNHG12 |
| hsa-miR-122 | SNHG12 |
| hsa-miR-338 | SNHG12 |
| hsa-miR-338 | SNHG12 |
| hsa-miR-33b | SNHG12 |
| hsa-miR-10b | SNHG12 |
| hsa-miR-10b | SNHG12 |
| hsa-miR-137 | AC004988.1.1 |
| hsa-miR-144 | AC004988.1.1 |
| hsa-miR-155 | AC004988.1.1 |
| hsa-miR-424 | AC004988.1.1 |
| hsa-miR-181c | AC004988.1.1 |
| hsa-miR-181d | AC004988.1.1 |
| hsa-miR-181c | AC004988.1.1 |
| hsa-miR-181d | AC004988.1.1 |
| hsa-miR-192 | AC004988.1.1 |
| hsa-miR-196b | AC004988.1.1 |
| hsa-miR-196b | AC004988.1.1 |
| hsa-miR-338 | AC004988.1.1 |
| hsa-miR-383 | IGBP1-AS1 |
| hsa-miR-301a | LINC00501 |
| hsa-miR-454 | LINC00501 |
| hsa-miR-301a | LINC00501 |
| hsa-miR-454 | LINC00501 |
| hsa-miR-152 | LINC00501 |
| hsa-miR-183 | LINC00501 |
| hsa-miR-200b | LINC00501 |
| hsa-miR-200c | LINC00501 |
| hsa-miR-429 | LINC00501 |
| hsa-miR-211 | LINC00501 |
| hsa-miR-23a | LINC00501 |
| hsa-miR-92b | LINC00501 |
| hsa-miR-338 | LINC00501 |
| hsa-miR-338 | LINC00501 |
| hsa-miR-338 | LINC00501 |
| hsa-miR-152 | TTLL10-AS1 |
| hsa-miR-22 | TTLL10-AS1 |
| hsa-miR-122 | TTLL10-AS1 |
| hsa-miR-23a | TTLL10-AS1 |
| hsa-miR-92b | TTLL10-AS1 |
| hsa-miR-10b | TTLL10-AS1 |
| hsa-miR-152 | FAM99B |
| hsa-miR-205 | FAM99B |
| hsa-miR-152 | FAM99A |
| hsa-miR-205 | FAM99A |
| hsa-miR-122 | FAM99A |
| hsa-miR-30a | FAM99A |
| hsa-miR-30b | FAM99A |
| hsa-miR-30d | FAM99A |
| hsa-miR-10b | FAM99A |
| hsa-miR-152 | H1FX-AS1 |
| hsa-miR-196b | H1FX-AS1 |
| hsa-miR-200b | H1FX-AS1 |
| hsa-miR-200c | H1FX-AS1 |
| hsa-miR-429 | H1FX-AS1 |
| hsa-miR-205 | H1FX-AS1 |
| hsa-miR-22 | H1FX-AS1 |
| hsa-miR-122 | H1FX-AS1 |
| hsa-miR-122 | H1FX-AS1 |
| hsa-miR-122 | H1FX-AS1 |
| hsa-miR-23a | H1FX-AS1 |
| hsa-miR-383 | H1FX-AS1 |
| hsa-miR-301a | AC084018.1.1 |
| hsa-miR-454 | AC084018.1.1 |
| hsa-miR-135b | AC084018.1.1 |
| hsa-miR-152 | AC084018.1.1 |
| hsa-miR-424 | AC084018.1.1 |
| hsa-miR-187 | AC084018.1.1 |
| hsa-miR-187 | AC084018.1.1 |
| hsa-miR-192 | AC084018.1.1 |
| hsa-miR-22 | AC084018.1.1 |
| hsa-miR-27a | AC084018.1.1 |
| hsa-miR-30a | AC084018.1.1 |
| hsa-miR-30b | AC084018.1.1 |
| hsa-miR-30d | AC084018.1.1 |
| hsa-miR-34c | AC084018.1.1 |
| hsa-miR-10b | AC084018.1.1 |
| hsa-miR-10b | AC084018.1.1 |
| hsa-miR-96 | UCA1 |
| hsa-miR-135b | UCA1 |
| hsa-miR-135b | UCA1 |
| hsa-miR-182 | UCA1 |
| hsa-miR-122 | UCA1 |
| hsa-miR-23a | UCA1 |
| hsa-miR-383 | UCA1 |
| hsa-miR-301a | MEG3 |
| hsa-miR-454 | MEG3 |
| hsa-miR-301a | MEG3 |
| hsa-miR-454 | MEG3 |
| hsa-miR-96 | MEG3 |
| hsa-miR-135b | MEG3 |
| hsa-miR-135b | MEG3 |
| hsa-miR-141 | MEG3 |
| hsa-miR-200a | MEG3 |
| hsa-miR-144 | MEG3 |
| hsa-miR-144 | MEG3 |
| hsa-miR-144 | MEG3 |
| hsa-miR-155 | MEG3 |
| hsa-miR-155 | MEG3 |
| hsa-miR-424 | MEG3 |
| hsa-miR-424 | MEG3 |
| hsa-miR-424 | MEG3 |
| hsa-miR-424 | MEG3 |
| hsa-miR-181c | MEG3 |
| hsa-miR-181d | MEG3 |
| hsa-miR-181c | MEG3 |
| hsa-miR-181d | MEG3 |
| hsa-miR-181c | MEG3 |
| hsa-miR-181d | MEG3 |
| hsa-miR-182 | MEG3 |
| hsa-miR-182 | MEG3 |
| hsa-miR-192 | MEG3 |
| hsa-miR-200b | MEG3 |
| hsa-miR-200c | MEG3 |
| hsa-miR-429 | MEG3 |
| hsa-miR-211 | MEG3 |
| hsa-miR-211 | MEG3 |
| hsa-miR-211 | MEG3 |
| hsa-miR-211 | MEG3 |
| hsa-miR-211 | MEG3 |
| hsa-miR-205 | MEG3 |
| hsa-miR-21 | MEG3 |
| hsa-miR-22 | MEG3 |
| hsa-miR-22 | MEG3 |
| hsa-miR-221 | MEG3 |
| hsa-miR-222 | MEG3 |
| hsa-miR-122 | MEG3 |
| hsa-miR-122 | MEG3 |
| hsa-miR-122 | MEG3 |
| hsa-miR-122 | MEG3 |
| hsa-miR-122 | MEG3 |
| hsa-miR-122 | MEG3 |
| hsa-miR-23a | MEG3 |
| hsa-miR-27a | MEG3 |
| hsa-miR-31 | MEG3 |
| hsa-miR-338 | MEG3 |
| hsa-miR-338 | MEG3 |
| hsa-miR-34c | MEG3 |
| hsa-miR-34c | MEG3 |
| hsa-miR-34c | MEG3 |
| hsa-miR-383 | MEG3 |
| hsa-miR-10b | MEG3 |
| hsa-miR-10b | MEG3 |
| hsa-miR-181c | FAM66B |
| hsa-miR-181d | FAM66B |
| hsa-miR-27a | FAM66B |
| hsa-miR-33b | FAM66B |
| hsa-miR-301a | MCM3AP-AS1 |
| hsa-miR-454 | MCM3AP-AS1 |
| hsa-miR-96 | MCM3AP-AS1 |
| hsa-miR-137 | MCM3AP-AS1 |
| hsa-miR-141 | MCM3AP-AS1 |
| hsa-miR-200a | MCM3AP-AS1 |
| hsa-miR-144 | MCM3AP-AS1 |
| hsa-miR-152 | MCM3AP-AS1 |
| hsa-miR-155 | MCM3AP-AS1 |
| hsa-miR-424 | MCM3AP-AS1 |
| hsa-miR-424 | MCM3AP-AS1 |
| hsa-miR-424 | MCM3AP-AS1 |
| hsa-miR-182 | MCM3AP-AS1 |
| hsa-miR-187 | MCM3AP-AS1 |
| hsa-miR-192 | MCM3AP-AS1 |
| hsa-miR-200b | MCM3AP-AS1 |
| hsa-miR-200c | MCM3AP-AS1 |
| hsa-miR-429 | MCM3AP-AS1 |
| hsa-miR-200b | MCM3AP-AS1 |
| hsa-miR-200c | MCM3AP-AS1 |
| hsa-miR-429 | MCM3AP-AS1 |
| hsa-miR-200b | MCM3AP-AS1 |
| hsa-miR-200c | MCM3AP-AS1 |
| hsa-miR-429 | MCM3AP-AS1 |
| hsa-miR-211 | MCM3AP-AS1 |
| hsa-miR-205 | MCM3AP-AS1 |
| hsa-miR-205 | MCM3AP-AS1 |
| hsa-miR-205 | MCM3AP-AS1 |
| hsa-miR-21 | MCM3AP-AS1 |
| hsa-miR-22 | MCM3AP-AS1 |
| hsa-miR-122 | MCM3AP-AS1 |
| hsa-miR-23a | MCM3AP-AS1 |
| hsa-miR-23a | MCM3AP-AS1 |
| hsa-miR-23a | MCM3AP-AS1 |
| hsa-miR-30a | MCM3AP-AS1 |
| hsa-miR-30b | MCM3AP-AS1 |
| hsa-miR-30d | MCM3AP-AS1 |
| hsa-miR-338 | MCM3AP-AS1 |
| hsa-miR-338 | MCM3AP-AS1 |
| hsa-miR-383 | MCM3AP-AS1 |
| hsa-miR-383 | MCM3AP-AS1 |
| hsa-miR-383 | MCM3AP-AS1 |
| hsa-miR-301a | AC112721.2.1 |
| hsa-miR-454 | AC112721.2.1 |
| hsa-miR-23a | AC112721.2.1 |
| hsa-miR-31 | AC112721.2.1 |
| hsa-miR-338 | AC112721.2.1 |
| hsa-miR-424 | AC093690.1.1 |
| hsa-miR-181c | AC093690.1.1 |
| hsa-miR-181d | AC093690.1.1 |
| hsa-miR-183 | AC093690.1.1 |
| hsa-miR-205 | AC093690.1.1 |
| hsa-miR-122 | AC093690.1.1 |
| hsa-miR-23a | AC093690.1.1 |
| hsa-miR-23a | AC093690.1.1 |
| hsa-miR-27a | AC093690.1.1 |
| hsa-miR-27a | AC093690.1.1 |
| hsa-miR-338 | AC093690.1.1 |
| hsa-miR-34c | AC093690.1.1 |
| hsa-miR-34c | AC093690.1.1 |
| hsa-miR-383 | AC093690.1.1 |
| hsa-miR-22 | AC106786.1.1 |
| hsa-miR-122 | AC106786.1.1 |
| hsa-miR-155 | LINC00205 |
| hsa-miR-141 | ENTPD3-AS1 |
| hsa-miR-200a | ENTPD3-AS1 |
| hsa-miR-155 | ENTPD3-AS1 |
| hsa-miR-181c | ENTPD3-AS1 |
| hsa-miR-181d | ENTPD3-AS1 |
| hsa-miR-211 | ENTPD3-AS1 |
| hsa-miR-205 | ENTPD3-AS1 |
| hsa-miR-21 | ENTPD3-AS1 |
| hsa-miR-27a | ENTPD3-AS1 |
| hsa-miR-338 | ENTPD3-AS1 |
| hsa-miR-338 | ENTPD3-AS1 |
| hsa-miR-383 | ENTPD3-AS1 |
| hsa-miR-10b | ENTPD3-AS1 |
| hsa-miR-10b | ENTPD3-AS1 |
| hsa-miR-137 | EFCAB6-AS1 |
| hsa-miR-137 | EFCAB6-AS1 |
| hsa-miR-200b | EFCAB6-AS1 |
| hsa-miR-200c | EFCAB6-AS1 |
| hsa-miR-429 | EFCAB6-AS1 |
| hsa-miR-211 | EFCAB6-AS1 |
| hsa-miR-205 | EFCAB6-AS1 |
| hsa-miR-30a | EFCAB6-AS1 |
| hsa-miR-30b | EFCAB6-AS1 |
| hsa-miR-30d | EFCAB6-AS1 |
| hsa-miR-383 | EFCAB6-AS1 |
| hsa-miR-144 | AC104809.2.1 |
| hsa-miR-205 | AC104809.2.1 |
| hsa-miR-22 | AC104809.2.1 |
| hsa-miR-92b | AC104809.2.1 |
| hsa-miR-27a | AC104809.2.1 |
| hsa-miR-34c | AC104809.2.1 |
| hsa-miR-34c | AC104809.2.1 |
| hsa-miR-10b | AC104809.2.1 |
| hsa-miR-141 | EPB41L4A-AS1 |
| hsa-miR-200a | EPB41L4A-AS1 |
| hsa-miR-152 | EPB41L4A-AS1 |
| hsa-miR-424 | EPB41L4A-AS1 |
| hsa-miR-183 | EPB41L4A-AS1 |
| hsa-miR-200b | EPB41L4A-AS1 |
| hsa-miR-200c | EPB41L4A-AS1 |
| hsa-miR-429 | EPB41L4A-AS1 |
| hsa-miR-200b | EPB41L4A-AS1 |
| hsa-miR-200c | EPB41L4A-AS1 |
| hsa-miR-429 | EPB41L4A-AS1 |
| hsa-miR-205 | EPB41L4A-AS1 |
| hsa-miR-22 | EPB41L4A-AS1 |
| hsa-miR-23a | EPB41L4A-AS1 |
| hsa-miR-30a | EPB41L4A-AS1 |
| hsa-miR-30b | EPB41L4A-AS1 |
| hsa-miR-30d | EPB41L4A-AS1 |
| hsa-miR-338 | EPB41L4A-AS1 |
| hsa-miR-34c | EPB41L4A-AS1 |
| hsa-miR-383 | EPB41L4A-AS1 |
| hsa-miR-196b | AC007319.1.1 |
| hsa-miR-205 | AC007319.1.1 |
| hsa-miR-205 | AC007319.1.1 |
| hsa-miR-22 | AC007319.1.1 |
| hsa-miR-23a | AC007319.1.1 |
| hsa-miR-27a | AC007319.1.1 |
| hsa-miR-30a | AC007319.1.1 |
| hsa-miR-30b | AC007319.1.1 |
| hsa-miR-30d | AC007319.1.1 |
| hsa-miR-383 | AC007319.1.1 |
| hsa-miR-383 | AC007319.1.1 |
| hsa-miR-301a | SNHG14 |
| hsa-miR-454 | SNHG14 |
| hsa-miR-301a | SNHG14 |
| hsa-miR-454 | SNHG14 |
| hsa-miR-301a | SNHG14 |
| hsa-miR-454 | SNHG14 |
| hsa-miR-301a | SNHG14 |
| hsa-miR-454 | SNHG14 |
| hsa-miR-301a | SNHG14 |
| hsa-miR-454 | SNHG14 |
| hsa-miR-301a | SNHG14 |
| hsa-miR-454 | SNHG14 |
| hsa-miR-301a | SNHG14 |
| hsa-miR-454 | SNHG14 |
| hsa-miR-301a | SNHG14 |
| hsa-miR-454 | SNHG14 |
| hsa-miR-301a | SNHG14 |
| hsa-miR-454 | SNHG14 |
| hsa-miR-301a | SNHG14 |
| hsa-miR-454 | SNHG14 |
| hsa-miR-301a | SNHG14 |
| hsa-miR-454 | SNHG14 |
| hsa-miR-96 | SNHG14 |
| hsa-miR-135b | SNHG14 |
| hsa-miR-135b | SNHG14 |
| hsa-miR-135b | SNHG14 |
| hsa-miR-137 | SNHG14 |
| hsa-miR-137 | SNHG14 |
| hsa-miR-137 | SNHG14 |
| hsa-miR-137 | SNHG14 |
| hsa-miR-141 | SNHG14 |
| hsa-miR-200a | SNHG14 |
| hsa-miR-141 | SNHG14 |
| hsa-miR-200a | SNHG14 |
| hsa-miR-141 | SNHG14 |
| hsa-miR-200a | SNHG14 |
| hsa-miR-141 | SNHG14 |
| hsa-miR-200a | SNHG14 |
| hsa-miR-141 | SNHG14 |
| hsa-miR-200a | SNHG14 |
| hsa-miR-141 | SNHG14 |
| hsa-miR-200a | SNHG14 |
| hsa-miR-141 | SNHG14 |
| hsa-miR-200a | SNHG14 |
| hsa-miR-141 | SNHG14 |
| hsa-miR-200a | SNHG14 |
| hsa-miR-141 | SNHG14 |
| hsa-miR-200a | SNHG14 |
| hsa-miR-144 | SNHG14 |
| hsa-miR-144 | SNHG14 |
| hsa-miR-144 | SNHG14 |
| hsa-miR-152 | SNHG14 |
| hsa-miR-152 | SNHG14 |
| hsa-miR-152 | SNHG14 |
| hsa-miR-152 | SNHG14 |
| hsa-miR-152 | SNHG14 |
| hsa-miR-152 | SNHG14 |
| hsa-miR-152 | SNHG14 |
| hsa-miR-152 | SNHG14 |
| hsa-miR-152 | SNHG14 |
| hsa-miR-152 | SNHG14 |
| hsa-miR-152 | SNHG14 |
| hsa-miR-152 | SNHG14 |
| hsa-miR-152 | SNHG14 |
| hsa-miR-152 | SNHG14 |
| hsa-miR-152 | SNHG14 |
| hsa-miR-152 | SNHG14 |
| hsa-miR-152 | SNHG14 |
| hsa-miR-152 | SNHG14 |
| hsa-miR-152 | SNHG14 |
| hsa-miR-152 | SNHG14 |
| hsa-miR-152 | SNHG14 |
| hsa-miR-152 | SNHG14 |
| hsa-miR-152 | SNHG14 |
| hsa-miR-152 | SNHG14 |
| hsa-miR-152 | SNHG14 |
| hsa-miR-152 | SNHG14 |
| hsa-miR-152 | SNHG14 |
| hsa-miR-152 | SNHG14 |
| hsa-miR-152 | SNHG14 |
| hsa-miR-152 | SNHG14 |
| hsa-miR-152 | SNHG14 |
| hsa-miR-152 | SNHG14 |
| hsa-miR-152 | SNHG14 |
| hsa-miR-152 | SNHG14 |
| hsa-miR-152 | SNHG14 |
| hsa-miR-152 | SNHG14 |
| hsa-miR-152 | SNHG14 |
| hsa-miR-152 | SNHG14 |
| hsa-miR-152 | SNHG14 |
| hsa-miR-152 | SNHG14 |
| hsa-miR-155 | SNHG14 |
| hsa-miR-155 | SNHG14 |
| hsa-miR-424 | SNHG14 |
| hsa-miR-424 | SNHG14 |
| hsa-miR-181c | SNHG14 |
| hsa-miR-181d | SNHG14 |
| hsa-miR-181c | SNHG14 |
| hsa-miR-181d | SNHG14 |
| hsa-miR-181c | SNHG14 |
| hsa-miR-181d | SNHG14 |
| hsa-miR-181c | SNHG14 |
| hsa-miR-181d | SNHG14 |
| hsa-miR-181c | SNHG14 |
| hsa-miR-181d | SNHG14 |
| hsa-miR-181c | SNHG14 |
| hsa-miR-181d | SNHG14 |
| hsa-miR-181c | SNHG14 |
| hsa-miR-181d | SNHG14 |
| hsa-miR-181c | SNHG14 |
| hsa-miR-181d | SNHG14 |
| hsa-miR-181c | SNHG14 |
| hsa-miR-181d | SNHG14 |
| hsa-miR-182 | SNHG14 |
| hsa-miR-182 | SNHG14 |
| hsa-miR-182 | SNHG14 |
| hsa-miR-182 | SNHG14 |
| hsa-miR-182 | SNHG14 |
| hsa-miR-182 | SNHG14 |
| hsa-miR-183 | SNHG14 |
| hsa-miR-183 | SNHG14 |
| hsa-miR-183 | SNHG14 |
| hsa-miR-192 | SNHG14 |
| hsa-miR-192 | SNHG14 |
| hsa-miR-192 | SNHG14 |
| hsa-miR-200b | SNHG14 |
| hsa-miR-200c | SNHG14 |
| hsa-miR-429 | SNHG14 |
| hsa-miR-200b | SNHG14 |
| hsa-miR-200c | SNHG14 |
| hsa-miR-429 | SNHG14 |
| hsa-miR-200b | SNHG14 |
| hsa-miR-200c | SNHG14 |
| hsa-miR-429 | SNHG14 |
| hsa-miR-200b | SNHG14 |
| hsa-miR-200c | SNHG14 |
| hsa-miR-429 | SNHG14 |
| hsa-miR-200b | SNHG14 |
| hsa-miR-200c | SNHG14 |
| hsa-miR-429 | SNHG14 |
| hsa-miR-200b | SNHG14 |
| hsa-miR-200c | SNHG14 |
| hsa-miR-429 | SNHG14 |
| hsa-miR-200b | SNHG14 |
| hsa-miR-200c | SNHG14 |
| hsa-miR-429 | SNHG14 |
| hsa-miR-211 | SNHG14 |
| hsa-miR-211 | SNHG14 |
| hsa-miR-211 | SNHG14 |
| hsa-miR-211 | SNHG14 |
| hsa-miR-205 | SNHG14 |
| hsa-miR-205 | SNHG14 |
| hsa-miR-205 | SNHG14 |
| hsa-miR-205 | SNHG14 |
| hsa-miR-205 | SNHG14 |
| hsa-miR-21 | SNHG14 |
| hsa-miR-22 | SNHG14 |
| hsa-miR-122 | SNHG14 |
| hsa-miR-122 | SNHG14 |
| hsa-miR-122 | SNHG14 |
| hsa-miR-23a | SNHG14 |
| hsa-miR-23a | SNHG14 |
| hsa-miR-23a | SNHG14 |
| hsa-miR-23a | SNHG14 |
| hsa-miR-23a | SNHG14 |
| hsa-miR-23a | SNHG14 |
| hsa-miR-23a | SNHG14 |
| hsa-miR-23a | SNHG14 |
| hsa-miR-92b | SNHG14 |
| hsa-miR-92b | SNHG14 |
| hsa-miR-92b | SNHG14 |
| hsa-miR-27a | SNHG14 |
| hsa-miR-27a | SNHG14 |
| hsa-miR-27a | SNHG14 |
| hsa-miR-27a | SNHG14 |
| hsa-miR-27a | SNHG14 |
| hsa-miR-30a | SNHG14 |
| hsa-miR-30b | SNHG14 |
| hsa-miR-30d | SNHG14 |
| hsa-miR-30a | SNHG14 |
| hsa-miR-30b | SNHG14 |
| hsa-miR-30d | SNHG14 |
| hsa-miR-31 | SNHG14 |
| hsa-miR-31 | SNHG14 |
| hsa-miR-31 | SNHG14 |
| hsa-miR-31 | SNHG14 |
| hsa-miR-31 | SNHG14 |
| hsa-miR-31 | SNHG14 |
| hsa-miR-338 | SNHG14 |
| hsa-miR-338 | SNHG14 |
| hsa-miR-338 | SNHG14 |
| hsa-miR-338 | SNHG14 |
| hsa-miR-338 | SNHG14 |
| hsa-miR-338 | SNHG14 |
| hsa-miR-338 | SNHG14 |
| hsa-miR-338 | SNHG14 |
| hsa-miR-338 | SNHG14 |
| hsa-miR-338 | SNHG14 |
| hsa-miR-33b | SNHG14 |
| hsa-miR-33b | SNHG14 |
| hsa-miR-33b | SNHG14 |
| hsa-miR-34c | SNHG14 |
| hsa-miR-34c | SNHG14 |
| hsa-miR-34c | SNHG14 |
| hsa-miR-34c | SNHG14 |
| hsa-miR-383 | SNHG14 |
| hsa-miR-383 | SNHG14 |
| hsa-miR-383 | SNHG14 |
| hsa-miR-10b | SNHG14 |
| hsa-miR-10b | SNHG14 |
| hsa-miR-144 | POU6F2-AS1 |
| hsa-miR-192 | POU6F2-AS1 |
| hsa-miR-92b | POU6F2-AS1 |
| hsa-miR-34c | POU6F2-AS1 |
| hsa-miR-30a | HCG14 |
| hsa-miR-30b | HCG14 |
| hsa-miR-30d | HCG14 |
| hsa-miR-96 | LINC00466 |
| hsa-miR-135b | LINC00466 |
| hsa-miR-137 | LINC00466 |
| hsa-miR-141 | LINC00466 |
| hsa-miR-200a | LINC00466 |
| hsa-miR-144 | LINC00466 |
| hsa-miR-152 | LINC00466 |
| hsa-miR-155 | LINC00466 |
| hsa-miR-155 | LINC00466 |
| hsa-miR-183 | LINC00466 |
| hsa-miR-192 | LINC00466 |
| hsa-miR-200b | LINC00466 |
| hsa-miR-200c | LINC00466 |
| hsa-miR-429 | LINC00466 |
| hsa-miR-211 | LINC00466 |
| hsa-miR-211 | LINC00466 |
| hsa-miR-211 | LINC00466 |
| hsa-miR-205 | LINC00466 |
| hsa-miR-205 | LINC00466 |
| hsa-miR-21 | LINC00466 |
| hsa-miR-22 | LINC00466 |
| hsa-miR-22 | LINC00466 |
| hsa-miR-23a | LINC00466 |
| hsa-miR-92b | LINC00466 |
| hsa-miR-92b | LINC00466 |
| hsa-miR-92b | LINC00466 |
| hsa-miR-27a | LINC00466 |
| hsa-miR-27a | LINC00466 |
| hsa-miR-33b | LINC00466 |
| hsa-miR-34c | LINC00466 |
| hsa-miR-205 | AP000345.2.1 |
| hsa-miR-10b | AP000345.2.1 |
| hsa-miR-22 | AC068831.3.1 |
| hsa-miR-152 | COL18A1-AS2 |
| hsa-miR-22 | AC016735.1.1 |
| hsa-miR-211 | AC015987.1.1 |
| hsa-miR-301a | TMEM72-AS1 |
| hsa-miR-454 | TMEM72-AS1 |
| hsa-miR-301a | TMEM72-AS1 |
| hsa-miR-454 | TMEM72-AS1 |
| hsa-miR-424 | TMEM72-AS1 |
| hsa-miR-23a | TMEM72-AS1 |
| hsa-miR-27a | TMEM72-AS1 |
| hsa-miR-27a | TMEM72-AS1 |
| hsa-miR-27a | TMEM72-AS1 |
| hsa-miR-338 | TMEM72-AS1 |
| hsa-miR-338 | TMEM72-AS1 |
| hsa-miR-34c | TMEM72-AS1 |
| hsa-miR-34c | TMEM72-AS1 |
| hsa-miR-155 | AL592494.1.1 |
| hsa-miR-135b | LINC00184 |
| hsa-miR-181c | LINC00184 |
| hsa-miR-181d | LINC00184 |
| hsa-miR-181c | LINC00184 |
| hsa-miR-181d | LINC00184 |
| hsa-miR-205 | LINC00184 |
| hsa-miR-22 | LINC00184 |
| hsa-miR-23a | LINC00184 |
| hsa-miR-23a | LINC00184 |
| hsa-miR-27a | LINC00184 |
| hsa-miR-31 | LINC00184 |
| hsa-miR-31 | LINC00184 |
| hsa-miR-338 | LINC00184 |
| hsa-miR-33b | LINC00184 |
| hsa-miR-34c | LINC00184 |
| hsa-miR-33b | AC000067.1.1 |
| hsa-miR-181c | LINC00337 |
| hsa-miR-181d | LINC00337 |
| hsa-miR-182 | LINC00337 |
| hsa-miR-23a | LINC00337 |
| hsa-miR-338 | LINC00337 |
| hsa-miR-383 | LINC00337 |
| hsa-miR-10b | LINC00337 |
| hsa-miR-301a | GRTP1-AS1 |
| hsa-miR-454 | GRTP1-AS1 |
| hsa-miR-30a | GRTP1-AS1 |
| hsa-miR-30b | GRTP1-AS1 |
| hsa-miR-30d | GRTP1-AS1 |
| hsa-miR-33b | GRTP1-AS1 |
| hsa-miR-424 | TRAPPC12-AS1 |
| hsa-miR-424 | TRAPPC12-AS1 |
| hsa-miR-205 | TRAPPC12-AS1 |
| hsa-miR-21 | TRAPPC12-AS1 |
| hsa-miR-21 | TRAPPC12-AS1 |
| hsa-miR-22 | TRAPPC12-AS1 |
| hsa-miR-34c | TRAPPC12-AS1 |
| hsa-miR-383 | TRAPPC12-AS1 |
| hsa-miR-135b | LINC00113 |
| hsa-miR-338 | LINC00113 |
| hsa-miR-424 | SFTA1P |
| hsa-miR-182 | SFTA1P |
| hsa-miR-211 | SFTA1P |
| hsa-miR-221 | SFTA1P |
| hsa-miR-222 | SFTA1P |
| hsa-miR-122 | SFTA1P |
| hsa-miR-301a | AP001626.1.1 |
| hsa-miR-454 | AP001626.1.1 |
| hsa-miR-141 | AP001626.1.1 |
| hsa-miR-200a | AP001626.1.1 |
| hsa-miR-182 | AP001626.1.1 |
| hsa-miR-183 | AP001626.1.1 |
| hsa-miR-183 | AP001626.1.1 |
| hsa-miR-205 | AP001626.1.1 |
| hsa-miR-22 | AP001626.1.1 |
| hsa-miR-22 | AP001626.1.1 |
| hsa-miR-22 | AP001626.1.1 |
| hsa-miR-27a | AP001626.1.1 |
| hsa-miR-34c | AP001626.1.1 |
| hsa-miR-34c | AP001626.1.1 |
| hsa-miR-10b | AP001626.1.1 |
| hsa-miR-301a | BOLA3-AS1 |
| hsa-miR-454 | BOLA3-AS1 |
| hsa-miR-152 | BOLA3-AS1 |
| hsa-miR-152 | BOLA3-AS1 |
| hsa-miR-182 | BOLA3-AS1 |
| hsa-miR-200b | BOLA3-AS1 |
| hsa-miR-200c | BOLA3-AS1 |
| hsa-miR-429 | BOLA3-AS1 |
| hsa-miR-211 | BOLA3-AS1 |
| hsa-miR-122 | BOLA3-AS1 |
| hsa-miR-338 | BOLA3-AS1 |
| hsa-miR-383 | BOLA3-AS1 |
| hsa-miR-10b | BOLA3-AS1 |
| hsa-miR-96 | LINC00475 |
| hsa-miR-135b | LINC00475 |
| hsa-miR-135b | LINC00475 |
| hsa-miR-137 | LINC00475 |
| hsa-miR-141 | LINC00475 |
| hsa-miR-200a | LINC00475 |
| hsa-miR-141 | LINC00475 |
| hsa-miR-200a | LINC00475 |
| hsa-miR-424 | LINC00475 |
| hsa-miR-182 | LINC00475 |
| hsa-miR-183 | LINC00475 |
| hsa-miR-211 | LINC00475 |
| hsa-miR-211 | LINC00475 |
| hsa-miR-205 | LINC00475 |
| hsa-miR-22 | LINC00475 |
| hsa-miR-27a | LINC00475 |
| hsa-miR-27a | LINC00475 |
| hsa-miR-30a | LINC00475 |
| hsa-miR-30b | LINC00475 |
| hsa-miR-30d | LINC00475 |
| hsa-miR-33b | LINC00475 |
| hsa-miR-34c | LINC00475 |
| hsa-miR-10b | LINC00475 |
| hsa-miR-10b | LINC00475 |
| hsa-miR-137 | NCBP2-AS1 |
| hsa-miR-301a | AP001627.1.1 |
| hsa-miR-454 | AP001627.1.1 |
| hsa-miR-135b | AP001627.1.1 |
| hsa-miR-122 | AP001627.1.1 |
| hsa-miR-122 | AP001627.1.1 |
| hsa-miR-92b | AP001627.1.1 |
| hsa-miR-10b | AP001627.1.1 |
| hsa-miR-10b | AP001627.1.1 |
| hsa-miR-135b | DBH-AS1 |
| hsa-miR-424 | DBH-AS1 |
| hsa-miR-211 | DBH-AS1 |
| hsa-miR-211 | DBH-AS1 |
| hsa-miR-205 | DBH-AS1 |
| hsa-miR-34c | DBH-AS1 |
| hsa-miR-34c | DBH-AS1 |
| hsa-miR-301a | MIAT |
| hsa-miR-454 | MIAT |
| hsa-miR-301a | MIAT |
| hsa-miR-454 | MIAT |
| hsa-miR-301a | MIAT |
| hsa-miR-454 | MIAT |
| hsa-miR-96 | MIAT |
| hsa-miR-141 | MIAT |
| hsa-miR-200a | MIAT |
| hsa-miR-141 | MIAT |
| hsa-miR-200a | MIAT |
| hsa-miR-152 | MIAT |
| hsa-miR-152 | MIAT |
| hsa-miR-155 | MIAT |
| hsa-miR-424 | MIAT |
| hsa-miR-424 | MIAT |
| hsa-miR-181c | MIAT |
| hsa-miR-181d | MIAT |
| hsa-miR-181c | MIAT |
| hsa-miR-181d | MIAT |
| hsa-miR-181c | MIAT |
| hsa-miR-181d | MIAT |
| hsa-miR-182 | MIAT |
| hsa-miR-182 | MIAT |
| hsa-miR-187 | MIAT |
| hsa-miR-187 | MIAT |
| hsa-miR-192 | MIAT |
| hsa-miR-192 | MIAT |
| hsa-miR-196b | MIAT |
| hsa-miR-211 | MIAT |
| hsa-miR-211 | MIAT |
| hsa-miR-211 | MIAT |
| hsa-miR-211 | MIAT |
| hsa-miR-205 | MIAT |
| hsa-miR-22 | MIAT |
| hsa-miR-22 | MIAT |
| hsa-miR-221 | MIAT |
| hsa-miR-222 | MIAT |
| hsa-miR-221 | MIAT |
| hsa-miR-222 | MIAT |
| hsa-miR-221 | MIAT |
| hsa-miR-222 | MIAT |
| hsa-miR-221 | MIAT |
| hsa-miR-222 | MIAT |
| hsa-miR-23a | MIAT |
| hsa-miR-23a | MIAT |
| hsa-miR-23a | MIAT |
| hsa-miR-92b | MIAT |
| hsa-miR-92b | MIAT |
| hsa-miR-27a | MIAT |
| hsa-miR-27a | MIAT |
| hsa-miR-338 | MIAT |
| hsa-miR-338 | MIAT |
| hsa-miR-338 | MIAT |
| hsa-miR-338 | MIAT |
| hsa-miR-33b | MIAT |
| hsa-miR-34c | MIAT |
| hsa-miR-10b | MIAT |
| hsa-miR-10b | MIAT |
| hsa-miR-10b | MIAT |
| hsa-miR-10b | MIAT |
| hsa-miR-211 | ZNF32-AS1 |
| hsa-miR-33b | ZNF32-AS1 |
| hsa-miR-301a | CFLAR-AS1 |
| hsa-miR-454 | CFLAR-AS1 |
| hsa-miR-424 | CFLAR-AS1 |
| hsa-miR-424 | CFLAR-AS1 |
| hsa-miR-205 | CFLAR-AS1 |
| hsa-miR-205 | CFLAR-AS1 |
| hsa-miR-96 | LINC00323 |
| hsa-miR-137 | LINC00323 |
| hsa-miR-424 | LINC00323 |
| hsa-miR-182 | LINC00323 |
| hsa-miR-211 | LINC00323 |
| hsa-miR-211 | LINC00323 |
| hsa-miR-22 | LINC00323 |
| hsa-miR-22 | LINC00323 |
| hsa-miR-92b | LINC00323 |
| hsa-miR-338 | LINC00323 |
| hsa-miR-34c | UPK1A-AS1 |
| hsa-miR-137 | AC016722.3.1 |
| hsa-miR-23a | AC016722.3.1 |
| hsa-miR-23a | AC016722.3.1 |
| hsa-miR-92b | AC016722.3.1 |
| hsa-miR-183 | LENG8-AS1 |
| hsa-miR-211 | LENG8-AS1 |
| hsa-miR-155 | FAM66C |
| hsa-miR-424 | FAM66C |
| hsa-miR-424 | FAM66C |
| hsa-miR-424 | FAM66C |
| hsa-miR-181c | FAM66C |
| hsa-miR-181d | FAM66C |
| hsa-miR-183 | FAM66C |
| hsa-miR-187 | FAM66C |
| hsa-miR-192 | FAM66C |
| hsa-miR-200b | FAM66C |
| hsa-miR-200c | FAM66C |
| hsa-miR-429 | FAM66C |
| hsa-miR-200b | FAM66C |
| hsa-miR-200c | FAM66C |
| hsa-miR-429 | FAM66C |
| hsa-miR-211 | FAM66C |
| hsa-miR-211 | FAM66C |
| hsa-miR-205 | FAM66C |
| hsa-miR-21 | FAM66C |
| hsa-miR-221 | FAM66C |
| hsa-miR-222 | FAM66C |
| hsa-miR-122 | FAM66C |
| hsa-miR-122 | FAM66C |
| hsa-miR-23a | FAM66C |
| hsa-miR-23a | FAM66C |
| hsa-miR-92b | FAM66C |
| hsa-miR-27a | FAM66C |
| hsa-miR-338 | FAM66C |
| hsa-miR-33b | FAM66C |
| hsa-miR-34c | FAM66C |
| hsa-miR-10b | FAM66C |
| hsa-miR-10b | FAM66C |
| hsa-miR-152 | AC007966.1.1 |
| hsa-miR-181c | AC007966.1.1 |
| hsa-miR-181d | AC007966.1.1 |
| hsa-miR-21 | AC007966.1.1 |
| hsa-miR-92b | AC007966.1.1 |
| hsa-miR-27a | AC007966.1.1 |
| hsa-miR-31 | AC007966.1.1 |
| hsa-miR-205 | AC109826.1.1 |
| hsa-miR-22 | AC109826.1.1 |
| hsa-miR-141 | AC007639.1.1 |
| hsa-miR-200a | AC007639.1.1 |
| hsa-miR-424 | AC007639.1.1 |
| hsa-miR-183 | AC007639.1.1 |
| hsa-miR-192 | AC007639.1.1 |
| hsa-miR-205 | AC007639.1.1 |
| hsa-miR-122 | AC007639.1.1 |
| hsa-miR-122 | AC007639.1.1 |
| hsa-miR-27a | AC007639.1.1 |
| hsa-miR-27a | AC007639.1.1 |
| hsa-miR-30a | AC007639.1.1 |
| hsa-miR-30b | AC007639.1.1 |
| hsa-miR-30d | AC007639.1.1 |
| hsa-miR-31 | AC007639.1.1 |
| hsa-miR-10b | AC007639.1.1 |
| hsa-miR-301a | HCG15 |
| hsa-miR-454 | HCG15 |
| hsa-miR-211 | HCG15 |
| hsa-miR-27a | HCG15 |
| hsa-miR-301a | AC105760.2.1 |
| hsa-miR-454 | AC105760.2.1 |
| hsa-miR-96 | AC105760.2.1 |
| hsa-miR-152 | AC105760.2.1 |
| hsa-miR-424 | AC105760.2.1 |
| hsa-miR-182 | AC105760.2.1 |
| hsa-miR-205 | AC105760.2.1 |
| hsa-miR-22 | AC105760.2.1 |
| hsa-miR-92b | AC105760.2.1 |
| hsa-miR-34c | AC105760.2.1 |
| hsa-miR-141 | MIS18A-AS1 |
| hsa-miR-200a | MIS18A-AS1 |
| hsa-miR-23a | MIS18A-AS1 |
| hsa-miR-34c | MIS18A-AS1 |
| hsa-miR-211 | LINC00310 |
| hsa-miR-22 | LINC00310 |
| hsa-miR-27a | LINC00310 |
| hsa-miR-27a | LINC00310 |
| hsa-miR-27a | LINC00310 |
| hsa-miR-338 | LINC00310 |
| hsa-miR-33b | LINC00310 |
| hsa-miR-301a | SOX21-AS1 |
| hsa-miR-454 | SOX21-AS1 |
| hsa-miR-152 | SOX21-AS1 |
| hsa-miR-338 | SOX21-AS1 |
| hsa-miR-338 | SOX21-AS1 |
| hsa-miR-205 | FAM66A |
| hsa-miR-221 | FAM66A |
| hsa-miR-222 | FAM66A |
| hsa-miR-27a | FAM66A |
| hsa-miR-155 | AC099684.1.1 |
| hsa-miR-187 | AC099684.1.1 |
| hsa-miR-211 | AC099684.1.1 |
| hsa-miR-22 | AC099684.1.1 |
| hsa-miR-221 | AC099684.1.1 |
| hsa-miR-222 | AC099684.1.1 |
| hsa-miR-338 | AC099684.1.1 |
| hsa-miR-338 | AC099684.1.1 |
| hsa-miR-137 | LMO7-AS1 |
| hsa-miR-181c | LMO7-AS1 |
| hsa-miR-181d | LMO7-AS1 |
| hsa-miR-196b | LMO7-AS1 |
| hsa-miR-211 | LMO7-AS1 |
| hsa-miR-122 | LMO7-AS1 |
| hsa-miR-96 | LINC00242 |
| hsa-miR-135b | LINC00242 |
| hsa-miR-141 | LINC00242 |
| hsa-miR-200a | LINC00242 |
| hsa-miR-152 | LINC00242 |
| hsa-miR-152 | LINC00242 |
| hsa-miR-155 | LINC00242 |
| hsa-miR-211 | LINC00242 |
| hsa-miR-211 | LINC00242 |
| hsa-miR-211 | LINC00242 |
| hsa-miR-221 | LINC00242 |
| hsa-miR-222 | LINC00242 |
| hsa-miR-92b | LINC00242 |
| hsa-miR-31 | LINC00242 |
| hsa-miR-34c | LINC00242 |
| hsa-miR-96 | LRRC3-AS1 |
| hsa-miR-182 | LRRC3-AS1 |
| hsa-miR-211 | LRRC3-AS1 |
| hsa-miR-23a | LRRC3-AS1 |
| hsa-miR-27a | LRRC3-AS1 |
| hsa-miR-183 | SZT2-AS1 |
| hsa-miR-135b | LINC00452 |
| hsa-miR-152 | LINC00452 |
| hsa-miR-424 | LINC00452 |
| hsa-miR-424 | LINC00452 |
| hsa-miR-187 | LINC00452 |
| hsa-miR-211 | LINC00452 |
| hsa-miR-21 | LINC00452 |
| hsa-miR-22 | LINC00452 |
| hsa-miR-22 | LINC00452 |
| hsa-miR-122 | LINC00452 |
| hsa-miR-27a | LINC00452 |
| hsa-miR-338 | LINC00452 |
| hsa-miR-338 | LINC00452 |
| hsa-miR-34c | LINC00452 |
| hsa-miR-383 | LINC00452 |
| hsa-miR-10b | LINC00452 |
| hsa-miR-144 | SACS-AS1 |
| hsa-miR-187 | SACS-AS1 |
| hsa-miR-205 | SACS-AS1 |
| hsa-miR-22 | SACS-AS1 |
| hsa-miR-34c | SACS-AS1 |
| hsa-miR-205 | MAST4-AS1 |
| hsa-miR-23a | MAST4-AS1 |
| hsa-miR-23a | MAST4-AS1 |
| hsa-miR-30a | MAST4-AS1 |
| hsa-miR-30b | MAST4-AS1 |
| hsa-miR-30d | MAST4-AS1 |
| hsa-miR-135b | U91324.1.1 |
| hsa-miR-135b | U91324.1.1 |
| hsa-miR-200b | U91324.1.1 |
| hsa-miR-200c | U91324.1.1 |
| hsa-miR-429 | U91324.1.1 |
| hsa-miR-205 | U91324.1.1 |
| hsa-miR-205 | U91324.1.1 |
| hsa-miR-96 | UBE2Q1-AS1 |
| hsa-miR-152 | UBE2Q1-AS1 |
| hsa-miR-182 | UBE2Q1-AS1 |
| hsa-miR-187 | UBE2Q1-AS1 |
| hsa-miR-211 | UBE2Q1-AS1 |
| hsa-miR-33b | UBE2Q1-AS1 |
| hsa-miR-34c | UBE2Q1-AS1 |
| hsa-miR-152 | AP001065.2.1 |
| hsa-miR-155 | AP001065.2.1 |
| hsa-miR-424 | AP001065.2.1 |
| hsa-miR-183 | AP001065.2.1 |
| hsa-miR-22 | AP001065.2.1 |
| hsa-miR-338 | AP001065.2.1 |
| hsa-miR-34c | AP001065.2.1 |
| hsa-miR-34c | AP001065.2.1 |
| hsa-miR-383 | AP001065.2.1 |
| hsa-miR-10b | AP001065.2.1 |
| hsa-miR-10b | AP001065.2.1 |
| hsa-miR-301a | LINC00443 |
| hsa-miR-454 | LINC00443 |
| hsa-miR-135b | LINC00443 |
| hsa-miR-135b | LINC00443 |
| hsa-miR-141 | LINC00443 |
| hsa-miR-200a | LINC00443 |
| hsa-miR-144 | LINC00443 |
| hsa-miR-152 | LINC00443 |
| hsa-miR-183 | LINC00443 |
| hsa-miR-211 | LINC00443 |
| hsa-miR-23a | LINC00443 |
| hsa-miR-92b | LINC00443 |
| hsa-miR-27a | LINC00443 |
| hsa-miR-33b | LINC00443 |
| hsa-miR-96 | DSCR9 |
| hsa-miR-182 | DSCR9 |
| hsa-miR-22 | DSCR9 |
| hsa-miR-122 | DSCR9 |
| hsa-miR-338 | DSCR9 |
| hsa-miR-10b | DSCR9 |
| hsa-miR-301a | THAP7-AS1 |
| hsa-miR-454 | THAP7-AS1 |
| hsa-miR-152 | THAP7-AS1 |
| hsa-miR-155 | THAP7-AS1 |
| hsa-miR-187 | THAP7-AS1 |
| hsa-miR-187 | THAP7-AS1 |
| hsa-miR-200b | THAP7-AS1 |
| hsa-miR-200c | THAP7-AS1 |
| hsa-miR-429 | THAP7-AS1 |
| hsa-miR-205 | THAP7-AS1 |
| hsa-miR-22 | THAP7-AS1 |
| hsa-miR-92b | THAP7-AS1 |
| hsa-miR-31 | THAP7-AS1 |
| hsa-miR-338 | THAP7-AS1 |
| hsa-miR-34c | THAP7-AS1 |
| hsa-miR-34c | THAP7-AS1 |
| hsa-miR-338 | ZNF32-AS2 |
| hsa-miR-10b | ZNF32-AS2 |
| hsa-miR-424 | AC114763.1.1 |
| hsa-miR-21 | AC114763.1.1 |
| hsa-miR-383 | AC114763.1.1 |
| hsa-miR-152 | AC093609.1.1 |
| hsa-miR-424 | AC093609.1.1 |
| hsa-miR-181c | AC093609.1.1 |
| hsa-miR-181d | AC093609.1.1 |
| hsa-miR-22 | AC093609.1.1 |
| hsa-miR-23a | AC093609.1.1 |
| hsa-miR-34c | AC093609.1.1 |
| hsa-miR-424 | HM13-AS1 |
| hsa-miR-181c | HM13-AS1 |
| hsa-miR-181d | HM13-AS1 |
| hsa-miR-187 | HM13-AS1 |
| hsa-miR-31 | HM13-AS1 |
| hsa-miR-31 | HM13-AS1 |
| hsa-miR-338 | HM13-AS1 |
| hsa-miR-27a | USP12-AS2 |
| hsa-miR-96 | AC092171.4.1 |
| hsa-miR-424 | AC092171.4.1 |
| hsa-miR-424 | AC092171.4.1 |
| hsa-miR-181c | AC092171.4.1 |
| hsa-miR-181d | AC092171.4.1 |
| hsa-miR-182 | AC092171.4.1 |
| hsa-miR-183 | AC092171.4.1 |
| hsa-miR-211 | AC092171.4.1 |
| hsa-miR-22 | AC092171.4.1 |
| hsa-miR-22 | AC092171.4.1 |
| hsa-miR-22 | AC092171.4.1 |
| hsa-miR-221 | AC092171.4.1 |
| hsa-miR-222 | AC092171.4.1 |
| hsa-miR-122 | AC092171.4.1 |
| hsa-miR-338 | AC092171.4.1 |
| hsa-miR-34c | AC092171.4.1 |
| hsa-miR-383 | AC092171.4.1 |
| hsa-miR-10b | AC092171.4.1 |
| hsa-miR-152 | AC023115.1.1 |
| hsa-miR-183 | AC023115.1.1 |
| hsa-miR-301a | HCG18 |
| hsa-miR-454 | HCG18 |
| hsa-miR-301a | HCG18 |
| hsa-miR-454 | HCG18 |
| hsa-miR-135b | HCG18 |
| hsa-miR-137 | HCG18 |
| hsa-miR-152 | HCG18 |
| hsa-miR-152 | HCG18 |
| hsa-miR-152 | HCG18 |
| hsa-miR-155 | HCG18 |
| hsa-miR-424 | HCG18 |
| hsa-miR-424 | HCG18 |
| hsa-miR-424 | HCG18 |
| hsa-miR-187 | HCG18 |
| hsa-miR-196b | HCG18 |
| hsa-miR-196b | HCG18 |
| hsa-miR-196b | HCG18 |
| hsa-miR-196b | HCG18 |
| hsa-miR-205 | HCG18 |
| hsa-miR-205 | HCG18 |
| hsa-miR-22 | HCG18 |
| hsa-miR-122 | HCG18 |
| hsa-miR-23a | HCG18 |
| hsa-miR-23a | HCG18 |
| hsa-miR-23a | HCG18 |
| hsa-miR-23a | HCG18 |
| hsa-miR-92b | HCG18 |
| hsa-miR-27a | HCG18 |
| hsa-miR-27a | HCG18 |
| hsa-miR-30a | HCG18 |
| hsa-miR-30b | HCG18 |
| hsa-miR-30d | HCG18 |
| hsa-miR-338 | HCG18 |
| hsa-miR-338 | HCG18 |
| hsa-miR-338 | HCG18 |
| hsa-miR-33b | HCG18 |
| hsa-miR-34c | HCG18 |
| hsa-miR-34c | HCG18 |
| hsa-miR-338 | WASIR2 |
| hsa-miR-27a | LINC00441 |
| hsa-miR-33b | LINC00441 |
| hsa-miR-33b | LINC00441 |
| hsa-miR-34c | LINC00441 |
| hsa-miR-152 | DLG3-AS1 |
| hsa-miR-205 | DLG3-AS1 |
| hsa-miR-22 | DLG3-AS1 |
| hsa-miR-221 | DLG3-AS1 |
| hsa-miR-222 | DLG3-AS1 |
| hsa-miR-99a | DLX6-AS1 |
| hsa-miR-99b | DLX6-AS1 |
| hsa-miR-135b | DLX6-AS1 |
| hsa-miR-135b | DLX6-AS1 |
| hsa-miR-135b | DLX6-AS1 |
| hsa-miR-141 | DLX6-AS1 |
| hsa-miR-200a | DLX6-AS1 |
| hsa-miR-144 | DLX6-AS1 |
| hsa-miR-152 | DLX6-AS1 |
| hsa-miR-155 | DLX6-AS1 |
| hsa-miR-424 | DLX6-AS1 |
| hsa-miR-181c | DLX6-AS1 |
| hsa-miR-181d | DLX6-AS1 |
| hsa-miR-192 | DLX6-AS1 |
| hsa-miR-192 | DLX6-AS1 |
| hsa-miR-196b | DLX6-AS1 |
| hsa-miR-200b | DLX6-AS1 |
| hsa-miR-200c | DLX6-AS1 |
| hsa-miR-429 | DLX6-AS1 |
| hsa-miR-200b | DLX6-AS1 |
| hsa-miR-200c | DLX6-AS1 |
| hsa-miR-429 | DLX6-AS1 |
| hsa-miR-211 | DLX6-AS1 |
| hsa-miR-22 | DLX6-AS1 |
| hsa-miR-122 | DLX6-AS1 |
| hsa-miR-23a | DLX6-AS1 |
| hsa-miR-27a | DLX6-AS1 |
| hsa-miR-31 | DLX6-AS1 |
| hsa-miR-338 | DLX6-AS1 |
| hsa-miR-383 | DLX6-AS1 |
| hsa-miR-10b | DLX6-AS1 |
| hsa-miR-135b | F10-AS1 |
| hsa-miR-182 | F10-AS1 |
| hsa-miR-196b | F10-AS1 |
| hsa-miR-92b | F10-AS1 |
| hsa-miR-152 | SLC6A1-AS1 |
| hsa-miR-424 | SLC6A1-AS1 |
| hsa-miR-22 | SLC6A1-AS1 |
| hsa-miR-27a | SLC6A1-AS1 |
| hsa-miR-27a | SLC6A1-AS1 |
| hsa-miR-338 | SLC6A1-AS1 |
| hsa-miR-338 | SLC6A1-AS1 |
| hsa-miR-383 | SLC6A1-AS1 |
| hsa-miR-205 | FAM215B |
| hsa-miR-27a | FAM215B |
| hsa-miR-27a | FAM215B |
| hsa-miR-31 | FAM215B |
| hsa-miR-31 | FAM215B |
| hsa-miR-22 | AC093627.7.1 |
| hsa-miR-27a | AC093627.7.1 |
| hsa-miR-30a | AC093627.7.1 |
| hsa-miR-30b | AC093627.7.1 |
| hsa-miR-30d | AC093627.7.1 |
| hsa-miR-31 | AC093627.7.1 |
| hsa-miR-424 | AC009495.3.1 |
| hsa-miR-181c | AC092296.1.1 |
| hsa-miR-181d | AC092296.1.1 |
| hsa-miR-183 | AC092296.1.1 |
| hsa-miR-187 | AC092296.1.1 |
| hsa-miR-205 | AC092296.1.1 |
| hsa-miR-31 | AC092296.1.1 |
| hsa-miR-152 | COL4A2-AS1 |
| hsa-miR-92b | DPYD-AS1 |
| hsa-miR-141 | LINC00342 |
| hsa-miR-200a | LINC00342 |
| hsa-miR-141 | LINC00342 |
| hsa-miR-200a | LINC00342 |
| hsa-miR-144 | LINC00342 |
| hsa-miR-424 | LINC00342 |
| hsa-miR-182 | LINC00342 |
| hsa-miR-183 | LINC00342 |
| hsa-miR-192 | LINC00342 |
| hsa-miR-211 | LINC00342 |
| hsa-miR-211 | LINC00342 |
| hsa-miR-205 | LINC00342 |
| hsa-miR-221 | LINC00342 |
| hsa-miR-222 | LINC00342 |
| hsa-miR-221 | LINC00342 |
| hsa-miR-222 | LINC00342 |
| hsa-miR-27a | LINC00342 |
| hsa-miR-31 | LINC00342 |
| hsa-miR-338 | LINC00342 |
| hsa-miR-33b | LINC00342 |
| hsa-miR-152 | SNHG7 |
| hsa-miR-181c | SNHG7 |
| hsa-miR-181d | SNHG7 |
| hsa-miR-182 | SNHG7 |
| hsa-miR-211 | SNHG7 |
| hsa-miR-22 | SNHG7 |
| hsa-miR-122 | SNHG7 |
| hsa-miR-122 | SNHG7 |
| hsa-miR-122 | SNHG7 |
| hsa-miR-338 | SNHG7 |
| hsa-miR-338 | SNHG7 |
| hsa-miR-33b | SNHG7 |
| hsa-miR-34c | SNHG7 |
| hsa-miR-34c | SNHG7 |
| hsa-miR-34c | SNHG7 |
| hsa-miR-10b | SNHG7 |
| hsa-miR-141 | LINC00472 |
| hsa-miR-200a | LINC00472 |
| hsa-miR-155 | LINC00472 |
| hsa-miR-155 | LINC00472 |
| hsa-miR-424 | LINC00472 |
| hsa-miR-196b | LINC00472 |
| hsa-miR-211 | LINC00472 |
| hsa-miR-211 | LINC00472 |
| hsa-miR-22 | LINC00472 |
| hsa-miR-23a | LINC00472 |
| hsa-miR-383 | LINC00472 |
| hsa-miR-96 | AC007743.1.1 |
| hsa-miR-135b | AC007743.1.1 |
| hsa-miR-155 | AC007743.1.1 |
| hsa-miR-182 | AC007743.1.1 |
| hsa-miR-211 | AC007743.1.1 |
| hsa-miR-211 | AC007743.1.1 |
| hsa-miR-21 | AC007743.1.1 |
| hsa-miR-22 | AC007743.1.1 |
| hsa-miR-122 | AC007743.1.1 |
| hsa-miR-92b | AC007743.1.1 |
| hsa-miR-92b | AC007743.1.1 |
| hsa-miR-27a | AC007743.1.1 |
| hsa-miR-31 | AC007743.1.1 |
| hsa-miR-33b | AC007743.1.1 |
| hsa-miR-137 | OSTN-AS1 |
| hsa-miR-27a | OSTN-AS1 |
| hsa-miR-181c | ZNF197-AS1 |
| hsa-miR-181d | ZNF197-AS1 |
| hsa-miR-23a | ZNF197-AS1 |
| hsa-miR-23a | ZNF197-AS1 |
| hsa-miR-27a | ZNF197-AS1 |
| hsa-miR-200b | LINC00460 |
| hsa-miR-200c | LINC00460 |
| hsa-miR-429 | LINC00460 |
| hsa-miR-221 | LINC00460 |
| hsa-miR-222 | LINC00460 |
| hsa-miR-23a | LINC00460 |
| hsa-miR-338 | LINC00460 |
| hsa-miR-338 | LINC00460 |
| hsa-miR-152 | AC007365.1.1 |
| hsa-miR-152 | AC007365.1.1 |
| hsa-miR-424 | AC007365.1.1 |
| hsa-miR-200b | AC007365.1.1 |
| hsa-miR-200c | AC007365.1.1 |
| hsa-miR-429 | AC007365.1.1 |
| hsa-miR-205 | AC007365.1.1 |
| hsa-miR-205 | AC007365.1.1 |
| hsa-miR-205 | AC007365.1.1 |
| hsa-miR-205 | AC007365.1.1 |
| hsa-miR-27a | AC007365.1.1 |
| hsa-miR-27a | AC007365.1.1 |
| hsa-miR-31 | AC007365.1.1 |
| hsa-miR-338 | AC139887.4.1 |
| hsa-miR-135b | AC004012.1.1 |
| hsa-miR-192 | AC004012.1.1 |
| hsa-miR-338 | AC004012.1.1 |
| hsa-miR-135b | TPRG1-AS1 |
| hsa-miR-182 | TPRG1-AS1 |
| hsa-miR-92b | TPRG1-AS1 |
| hsa-miR-338 | TPRG1-AS1 |
| hsa-miR-137 | AC009264.1.1 |
| hsa-miR-137 | AC009264.1.1 |
| hsa-miR-181c | AC009264.1.1 |
| hsa-miR-181d | AC009264.1.1 |
| hsa-miR-211 | AC009264.1.1 |
| hsa-miR-23a | AC009264.1.1 |
| hsa-miR-23a | AC009264.1.1 |
| hsa-miR-92b | AC009264.1.1 |
| hsa-miR-92b | AC009264.1.1 |
| hsa-miR-27a | AC147651.1.1 |
| hsa-miR-96 | AP000253.1.1 |
| hsa-miR-141 | AP000253.1.1 |
| hsa-miR-200a | AP000253.1.1 |
| hsa-miR-141 | AP000253.1.1 |
| hsa-miR-200a | AP000253.1.1 |
| hsa-miR-141 | AP000253.1.1 |
| hsa-miR-200a | AP000253.1.1 |
| hsa-miR-182 | AP000253.1.1 |
| hsa-miR-182 | AP000253.1.1 |
| hsa-miR-187 | AP000253.1.1 |
| hsa-miR-22 | AP000253.1.1 |
| hsa-miR-30a | AP000253.1.1 |
| hsa-miR-30b | AP000253.1.1 |
| hsa-miR-30d | AP000253.1.1 |
| hsa-miR-31 | AP000253.1.1 |
| hsa-miR-31 | AP000253.1.1 |
| hsa-miR-34c | AP000253.1.1 |
| hsa-miR-10b | AP000253.1.1 |
| hsa-miR-141 | SNRK-AS1 |
| hsa-miR-200a | SNRK-AS1 |
| hsa-miR-211 | SNRK-AS1 |
| hsa-miR-92b | SNRK-AS1 |
| hsa-miR-10b | SNRK-AS1 |
| hsa-miR-301a | HMGA1P4 |
| hsa-miR-454 | HMGA1P4 |
| hsa-miR-192 | HMGA1P4 |
| hsa-miR-96 | GAS5 |
| hsa-miR-135b | GAS5 |
| hsa-miR-135b | GAS5 |
| hsa-miR-137 | GAS5 |
| hsa-miR-144 | GAS5 |
| hsa-miR-152 | GAS5 |
| hsa-miR-155 | GAS5 |
| hsa-miR-182 | GAS5 |
| hsa-miR-196b | GAS5 |
| hsa-miR-200b | GAS5 |
| hsa-miR-200c | GAS5 |
| hsa-miR-429 | GAS5 |
| hsa-miR-205 | GAS5 |
| hsa-miR-205 | GAS5 |
| hsa-miR-21 | GAS5 |
| hsa-miR-221 | GAS5 |
| hsa-miR-222 | GAS5 |
| hsa-miR-23a | GAS5 |
| hsa-miR-23a | GAS5 |
| hsa-miR-31 | GAS5 |
| hsa-miR-31 | GAS5 |
| hsa-miR-10b | GAS5 |
| hsa-miR-205 | ZRANB2-AS1 |
| hsa-miR-383 | ZRANB2-AS1 |
| hsa-miR-205 | AC012506.4.1 |
| hsa-miR-221 | AC012506.4.1 |
| hsa-miR-222 | AC012506.4.1 |
| hsa-miR-92b | AC012506.4.1 |
| hsa-miR-27a | AC012506.4.1 |
| hsa-miR-27a | AC012506.4.1 |
| hsa-miR-135b | LINC00402 |
| hsa-miR-137 | LINC00402 |
| hsa-miR-141 | LINC00402 |
| hsa-miR-200a | LINC00402 |
| hsa-miR-155 | LINC00402 |
| hsa-miR-181c | LINC00402 |
| hsa-miR-181d | LINC00402 |
| hsa-miR-181c | LINC00402 |
| hsa-miR-181d | LINC00402 |
| hsa-miR-182 | LINC00402 |
| hsa-miR-200b | LINC00402 |
| hsa-miR-200c | LINC00402 |
| hsa-miR-429 | LINC00402 |
| hsa-miR-211 | LINC00402 |
| hsa-miR-22 | LINC00402 |
| hsa-miR-27a | LINC00402 |
| hsa-miR-27a | LINC00402 |
| hsa-miR-338 | LINC00402 |
| hsa-miR-338 | LINC00402 |
| hsa-miR-33b | LINC00402 |
| hsa-miR-383 | LINC00402 |
| hsa-miR-383 | LINC00402 |
| hsa-miR-181c | LINC00494 |
| hsa-miR-181d | LINC00494 |
| hsa-miR-182 | LINC00494 |
| hsa-miR-182 | LINC00494 |
| hsa-miR-221 | LINC00494 |
| hsa-miR-222 | LINC00494 |
| hsa-miR-23a | LINC00494 |
| hsa-miR-27a | LINC00494 |
| hsa-miR-30a | LINC00494 |
| hsa-miR-30b | LINC00494 |
| hsa-miR-30d | LINC00494 |
| hsa-miR-31 | LINC00494 |
| hsa-miR-31 | LINC00494 |
| hsa-miR-31 | LINC00494 |
| hsa-miR-383 | LINC00494 |
| hsa-miR-10b | LINC00494 |
| hsa-miR-99a | SAPCD1-AS1 |
| hsa-miR-99b | SAPCD1-AS1 |
| hsa-miR-187 | SAPCD1-AS1 |
| hsa-miR-196b | SAPCD1-AS1 |
| hsa-miR-211 | SAPCD1-AS1 |
| hsa-miR-141 | TM4SF19-AS1 |
| hsa-miR-200a | TM4SF19-AS1 |
| hsa-miR-211 | TM4SF19-AS1 |
| hsa-miR-205 | TM4SF19-AS1 |
| hsa-miR-23a | TM4SF19-AS1 |
| hsa-miR-30a | TM4SF19-AS1 |
| hsa-miR-30b | TM4SF19-AS1 |
| hsa-miR-30d | TM4SF19-AS1 |
| hsa-miR-34c | TM4SF19-AS1 |
| hsa-miR-22 | CPB2-AS1 |
| hsa-miR-152 | NEXN-AS1 |
| hsa-miR-211 | NEXN-AS1 |
| hsa-miR-92b | NEXN-AS1 |
| hsa-miR-135b | EGOT |
| hsa-miR-141 | EGOT |
| hsa-miR-200a | EGOT |
| hsa-miR-424 | EGOT |
| hsa-miR-183 | EGOT |
| hsa-miR-205 | EGOT |
| hsa-miR-21 | EGOT |
| hsa-miR-23a | EGOT |
| hsa-miR-23a | EGOT |
| hsa-miR-33b | EGOT |
| hsa-miR-33b | EGOT |
| hsa-miR-10b | EGOT |
| hsa-miR-96 | AC092687.3.1 |
| hsa-miR-21 | AC092687.3.1 |
| hsa-miR-23a | AC092687.3.1 |
| hsa-miR-31 | AC092687.3.1 |
| hsa-miR-33b | AC092687.3.1 |
| hsa-miR-33b | AC092687.3.1 |
| hsa-miR-96 | LINC00479 |
| hsa-miR-141 | LINC00479 |
| hsa-miR-200a | LINC00479 |
| hsa-miR-141 | LINC00479 |
| hsa-miR-200a | LINC00479 |
| hsa-miR-424 | LINC00479 |
| hsa-miR-181c | LINC00479 |
| hsa-miR-181d | LINC00479 |
| hsa-miR-182 | LINC00479 |
| hsa-miR-211 | LINC00479 |
| hsa-miR-205 | LINC00479 |
| hsa-miR-23a | LINC00479 |
| hsa-miR-33b | LINC00479 |
| hsa-miR-34c | LINC00479 |
| hsa-miR-10b | LINC00479 |
| hsa-miR-144 | ZEB1-AS1 |
| hsa-miR-211 | ZEB1-AS1 |
| hsa-miR-205 | ZEB1-AS1 |
| hsa-miR-92b | ZEB1-AS1 |
| hsa-miR-31 | ZEB1-AS1 |
| hsa-miR-144 | PRMT5-AS1 |
| hsa-miR-196b | PRMT5-AS1 |
| hsa-miR-205 | PRMT5-AS1 |
| hsa-miR-205 | PRMT5-AS1 |
| hsa-miR-221 | PRMT5-AS1 |
| hsa-miR-222 | PRMT5-AS1 |
| hsa-miR-221 | PRMT5-AS1 |
| hsa-miR-222 | PRMT5-AS1 |
| hsa-miR-31 | PRMT5-AS1 |
| hsa-miR-338 | PRMT5-AS1 |
| hsa-miR-137 | AC005550.3.1 |
| hsa-miR-155 | AC005550.3.1 |
| hsa-miR-196b | AC005550.3.1 |
| hsa-miR-196b | AC005550.3.1 |
| hsa-miR-221 | AC005550.3.1 |
| hsa-miR-222 | AC005550.3.1 |
| hsa-miR-92b | AC005550.3.1 |
| hsa-miR-92b | AC005550.3.1 |
| hsa-miR-27a | AC005550.3.1 |
| hsa-miR-27a | AC005550.3.1 |
| hsa-miR-31 | AC005550.3.1 |
| hsa-miR-338 | AC005550.3.1 |
| hsa-miR-34c | AC005550.3.1 |
| hsa-miR-383 | AC005550.3.1 |
| hsa-miR-21 | GS1-124K5.4.1 |
| hsa-miR-27a | FTCD-AS1 |
| hsa-miR-27a | FTCD-AS1 |
| hsa-miR-338 | FTCD-AS1 |
| hsa-miR-137 | AC007386.2.1 |
| hsa-miR-141 | AC007386.2.1 |
| hsa-miR-200a | AC007386.2.1 |
| hsa-miR-144 | AC007386.2.1 |
| hsa-miR-182 | AC007386.2.1 |
| hsa-miR-205 | AC007386.2.1 |
| hsa-miR-122 | AC007386.2.1 |
| hsa-miR-122 | AC007386.2.1 |
| hsa-miR-23a | AC007386.2.1 |
| hsa-miR-23a | AC007386.2.1 |
| hsa-miR-27a | AC007386.2.1 |
| hsa-miR-31 | AC007386.2.1 |
| hsa-miR-187 | LINC00316 |
| hsa-miR-122 | LINC00316 |
| hsa-miR-338 | LINC00316 |
| hsa-miR-33b | LINC00316 |
| hsa-miR-424 | LINC00398 |
| hsa-miR-205 | LINC00398 |
| hsa-miR-338 | LINC00398 |
| hsa-miR-383 | LINC00398 |
| hsa-miR-135b | DGUOK-AS1 |
| hsa-miR-424 | DGUOK-AS1 |
| hsa-miR-211 | DGUOK-AS1 |
| hsa-miR-211 | DGUOK-AS1 |
| hsa-miR-96 | AC002480.2.1 |
| hsa-miR-96 | AC002480.2.1 |
| hsa-miR-144 | AC002480.2.1 |
| hsa-miR-181c | AC002480.2.1 |
| hsa-miR-181d | AC002480.2.1 |
| hsa-miR-182 | AC002480.2.1 |
| hsa-miR-221 | AC002480.2.1 |
| hsa-miR-222 | AC002480.2.1 |
| hsa-miR-92b | AC002480.2.1 |
| hsa-miR-383 | AC002480.2.1 |
| hsa-miR-135b | AC068196.1.1 |
| hsa-miR-137 | CLRN1-AS1 |
| hsa-miR-424 | CLRN1-AS1 |
| hsa-miR-181c | CLRN1-AS1 |
| hsa-miR-181d | CLRN1-AS1 |
| hsa-miR-200b | CLRN1-AS1 |
| hsa-miR-200c | CLRN1-AS1 |
| hsa-miR-429 | CLRN1-AS1 |
| hsa-miR-211 | CLRN1-AS1 |
| hsa-miR-205 | CLRN1-AS1 |
| hsa-miR-205 | CLRN1-AS1 |
| hsa-miR-22 | CLRN1-AS1 |
| hsa-miR-22 | CLRN1-AS1 |
| hsa-miR-221 | CLRN1-AS1 |
| hsa-miR-222 | CLRN1-AS1 |
| hsa-miR-221 | CLRN1-AS1 |
| hsa-miR-222 | CLRN1-AS1 |
| hsa-miR-27a | CLRN1-AS1 |
| hsa-miR-27a | CLRN1-AS1 |
| hsa-miR-338 | CLRN1-AS1 |
| hsa-miR-33b | CLRN1-AS1 |
| hsa-miR-338 | ST3GAL6-AS1 |
| hsa-miR-141 | MYLK-AS1 |
| hsa-miR-200a | MYLK-AS1 |
| hsa-miR-424 | MYLK-AS1 |
| hsa-miR-182 | MYLK-AS1 |
| hsa-miR-192 | MYLK-AS1 |
| hsa-miR-205 | MYLK-AS1 |
| hsa-miR-31 | MYLK-AS1 |
| hsa-miR-34c | MYLK-AS1 |
| hsa-miR-424 | ITIH4-AS1 |
| hsa-miR-211 | ITIH4-AS1 |
| hsa-miR-122 | ITIH4-AS1 |
| hsa-miR-141 | TM4SF1-AS1 |
| hsa-miR-200a | TM4SF1-AS1 |
| hsa-miR-30a | TM4SF1-AS1 |
| hsa-miR-30b | TM4SF1-AS1 |
| hsa-miR-30d | TM4SF1-AS1 |
| hsa-miR-34c | TM4SF1-AS1 |
| hsa-miR-137 | ARHGAP31-AS1 |
| hsa-miR-122 | ARHGAP31-AS1 |
| hsa-miR-135b | WWTR1-AS1 |
| hsa-miR-152 | WWTR1-AS1 |
| hsa-miR-196b | WWTR1-AS1 |
| hsa-miR-211 | WWTR1-AS1 |
| hsa-miR-21 | WWTR1-AS1 |
| hsa-miR-92b | WWTR1-AS1 |
| hsa-miR-338 | WWTR1-AS1 |
| hsa-miR-338 | WWTR1-AS1 |
| hsa-miR-10b | WWTR1-AS1 |
| hsa-miR-96 | KLHL6-AS1 |
| hsa-miR-135b | KLHL6-AS1 |
| hsa-miR-137 | KLHL6-AS1 |
| hsa-miR-144 | KLHL6-AS1 |
| hsa-miR-92b | KLHL6-AS1 |
| hsa-miR-96 | AC007620.3.1 |
| hsa-miR-182 | AC007620.3.1 |
| hsa-miR-122 | AC007620.3.1 |
| hsa-miR-92b | AC007620.3.1 |
| hsa-miR-181c | ZBTB20-AS4 |
| hsa-miR-181d | ZBTB20-AS4 |
| hsa-miR-200b | ZBTB20-AS4 |
| hsa-miR-200c | ZBTB20-AS4 |
| hsa-miR-429 | ZBTB20-AS4 |
| hsa-miR-205 | ZBTB20-AS4 |
| hsa-miR-33b | ZBTB20-AS4 |
| hsa-miR-200b | GK-AS1 |
| hsa-miR-200c | GK-AS1 |
| hsa-miR-429 | GK-AS1 |
| hsa-miR-34c | GK-AS1 |
| hsa-miR-135b | ARHGEF26-AS1 |
| hsa-miR-141 | ARHGEF26-AS1 |
| hsa-miR-200a | ARHGEF26-AS1 |
| hsa-miR-155 | ARHGEF26-AS1 |
| hsa-miR-187 | ARHGEF26-AS1 |
| hsa-miR-192 | ARHGEF26-AS1 |
| hsa-miR-211 | ARHGEF26-AS1 |
| hsa-miR-211 | ARHGEF26-AS1 |
| hsa-miR-205 | ARHGEF26-AS1 |
| hsa-miR-21 | ARHGEF26-AS1 |
| hsa-miR-23a | ARHGEF26-AS1 |
| hsa-miR-92b | ARHGEF26-AS1 |
| hsa-miR-27a | ARHGEF26-AS1 |
| hsa-miR-33b | ARHGEF26-AS1 |
| hsa-miR-10b | ARHGEF26-AS1 |
| hsa-miR-96 | MCCC1-AS1 |
| hsa-miR-152 | MCCC1-AS1 |
| hsa-miR-182 | MCCC1-AS1 |
| hsa-miR-183 | MCCC1-AS1 |
| hsa-miR-192 | MCCC1-AS1 |
| hsa-miR-92b | MCCC1-AS1 |
| hsa-miR-301a | HOTTIP |
| hsa-miR-454 | HOTTIP |
| hsa-miR-137 | HOTTIP |
| hsa-miR-141 | HOTTIP |
| hsa-miR-200a | HOTTIP |
| hsa-miR-152 | HOTTIP |
| hsa-miR-424 | HOTTIP |
| hsa-miR-424 | HOTTIP |
| hsa-miR-181c | HOTTIP |
| hsa-miR-181d | HOTTIP |
| hsa-miR-187 | HOTTIP |
| hsa-miR-192 | HOTTIP |
| hsa-miR-211 | HOTTIP |
| hsa-miR-205 | HOTTIP |
| hsa-miR-27a | HOTTIP |
| hsa-miR-30a | HOTTIP |
| hsa-miR-30b | HOTTIP |
| hsa-miR-30d | HOTTIP |
| hsa-miR-31 | HOTTIP |
| hsa-miR-338 | HOTTIP |
| hsa-miR-338 | HOTTIP |
| hsa-miR-33b | HOTTIP |
| hsa-miR-34c | HOTTIP |
| hsa-miR-338 | SIAH2-AS1 |
| hsa-miR-22 | WNT5A-AS1 |
| hsa-miR-135b | CRNDE |
| hsa-miR-144 | CRNDE |
| hsa-miR-144 | CRNDE |
| hsa-miR-155 | CRNDE |
| hsa-miR-181c | CRNDE |
| hsa-miR-181d | CRNDE |
| hsa-miR-183 | CRNDE |
| hsa-miR-205 | CRNDE |
| hsa-miR-22 | CRNDE |
| hsa-miR-221 | CRNDE |
| hsa-miR-222 | CRNDE |
| hsa-miR-23a | CRNDE |
| hsa-miR-92b | CRNDE |
| hsa-miR-27a | CRNDE |
| hsa-miR-27a | CRNDE |
| hsa-miR-31 | CRNDE |
| hsa-miR-338 | CRNDE |
| hsa-miR-135b | GS1-24F4.2.1 |
| hsa-miR-183 | GS1-24F4.2.1 |
| hsa-miR-211 | GS1-24F4.2.1 |
| hsa-miR-211 | GS1-24F4.2.1 |
| hsa-miR-33b | GS1-24F4.2.1 |
| hsa-miR-135b | SNHG6 |
| hsa-miR-137 | SNHG6 |
| hsa-miR-144 | SNHG6 |
| hsa-miR-181c | SNHG6 |
| hsa-miR-181d | SNHG6 |
| hsa-miR-200b | SNHG6 |
| hsa-miR-200c | SNHG6 |
| hsa-miR-429 | SNHG6 |
| hsa-miR-211 | SNHG6 |
| hsa-miR-205 | SNHG6 |
| hsa-miR-22 | SNHG6 |
| hsa-miR-27a | SNHG6 |
| hsa-miR-30a | SNHG6 |
| hsa-miR-30b | SNHG6 |
| hsa-miR-30d | SNHG6 |
| hsa-miR-34c | SNHG6 |
| hsa-miR-301a | ALDH1L1-AS2 |
| hsa-miR-454 | ALDH1L1-AS2 |
| hsa-miR-424 | ALDH1L1-AS2 |
| hsa-miR-23a | ALDH1L1-AS2 |
| hsa-miR-96 | AC226118.1.1 |
| hsa-miR-99a | AC226118.1.1 |
| hsa-miR-99b | AC226118.1.1 |
| hsa-miR-152 | AC226118.1.1 |
| hsa-miR-187 | AC226118.1.1 |
| hsa-miR-211 | AC226118.1.1 |
| hsa-miR-211 | AC226118.1.1 |
| hsa-miR-211 | AC226118.1.1 |
| hsa-miR-22 | AC226118.1.1 |
| hsa-miR-122 | AC226118.1.1 |
| hsa-miR-338 | AC226118.1.1 |
| hsa-miR-338 | AC226118.1.1 |
| hsa-miR-338 | AC226118.1.1 |
| hsa-miR-33b | AC226118.1.1 |
| hsa-miR-187 | EAF1-AS1 |
| hsa-miR-196b | EAF1-AS1 |
| hsa-miR-34c | EAF1-AS1 |
| hsa-miR-152 | PVT1 |
| hsa-miR-424 | PVT1 |
| hsa-miR-181c | PVT1 |
| hsa-miR-181d | PVT1 |
| hsa-miR-181c | PVT1 |
| hsa-miR-181d | PVT1 |
| hsa-miR-183 | PVT1 |
| hsa-miR-183 | PVT1 |
| hsa-miR-187 | PVT1 |
| hsa-miR-187 | PVT1 |
| hsa-miR-205 | PVT1 |
| hsa-miR-205 | PVT1 |
| hsa-miR-21 | PVT1 |
| hsa-miR-221 | PVT1 |
| hsa-miR-222 | PVT1 |
| hsa-miR-23a | PVT1 |
| hsa-miR-23a | PVT1 |
| hsa-miR-27a | PVT1 |
| hsa-miR-30a | PVT1 |
| hsa-miR-30b | PVT1 |
| hsa-miR-30d | PVT1 |
| hsa-miR-30a | PVT1 |
| hsa-miR-30b | PVT1 |
| hsa-miR-30d | PVT1 |
| hsa-miR-30a | PVT1 |
| hsa-miR-30b | PVT1 |
| hsa-miR-30d | PVT1 |
| hsa-miR-31 | PVT1 |
| hsa-miR-34c | PVT1 |
| hsa-miR-383 | PVT1 |
| hsa-miR-383 | PVT1 |
| hsa-miR-152 | ALDH1L1-AS1 |
| hsa-miR-192 | ALDH1L1-AS1 |
| hsa-miR-22 | ALDH1L1-AS1 |
| hsa-miR-301a | AC141928.1.1 |
| hsa-miR-454 | AC141928.1.1 |
| hsa-miR-96 | AC141928.1.1 |
| hsa-miR-135b | AC141928.1.1 |
| hsa-miR-152 | AC141928.1.1 |
| hsa-miR-152 | AC141928.1.1 |
| hsa-miR-152 | AC141928.1.1 |
| hsa-miR-424 | AC141928.1.1 |
| hsa-miR-182 | AC141928.1.1 |
| hsa-miR-211 | AC141928.1.1 |
| hsa-miR-211 | AC141928.1.1 |
| hsa-miR-205 | AC141928.1.1 |
| hsa-miR-122 | AC141928.1.1 |
| hsa-miR-27a | AC141928.1.1 |
| hsa-miR-34c | AC141928.1.1 |
| hsa-miR-34c | AC141928.1.1 |
| hsa-miR-10b | AC141928.1.1 |
| hsa-miR-137 | HULC |
| hsa-miR-155 | HULC |
| hsa-miR-211 | HULC |
| hsa-miR-27a | HULC |
| hsa-miR-383 | HULC |
| hsa-miR-301a | AC008592.4.1 |
| hsa-miR-454 | AC008592.4.1 |
| hsa-miR-152 | AC008592.4.1 |
| hsa-miR-152 | AC008592.4.1 |
| hsa-miR-23a | AC008592.4.1 |
| hsa-miR-96 | AC012613.2.1 |
| hsa-miR-96 | AC012613.2.1 |
| hsa-miR-182 | AC012613.2.1 |
| hsa-miR-182 | AC012613.2.1 |
| hsa-miR-183 | AC012613.2.1 |
| hsa-miR-192 | AC012613.2.1 |
| hsa-miR-192 | AC012613.2.1 |
| hsa-miR-200b | AC012613.2.1 |
| hsa-miR-200c | AC012613.2.1 |
| hsa-miR-429 | AC012613.2.1 |
| hsa-miR-211 | AC012613.2.1 |
| hsa-miR-205 | AC012613.2.1 |
| hsa-miR-21 | AC012613.2.1 |
| hsa-miR-221 | AC012613.2.1 |
| hsa-miR-222 | AC012613.2.1 |
| hsa-miR-122 | AC012613.2.1 |
| hsa-miR-122 | AC012613.2.1 |
| hsa-miR-122 | AC012613.2.1 |
| hsa-miR-122 | AC012613.2.1 |
| hsa-miR-27a | AC012613.2.1 |
| hsa-miR-31 | AC012613.2.1 |
| hsa-miR-33b | AC012613.2.1 |
| hsa-miR-34c | AC012613.2.1 |
| hsa-miR-34c | AC012613.2.1 |
| hsa-miR-424 | CTB-178M22.2.1 |
| hsa-miR-30a | CTB-178M22.2.1 |
| hsa-miR-30b | CTB-178M22.2.1 |
| hsa-miR-30d | CTB-178M22.2.1 |
| hsa-miR-211 | AC008691.1.1 |
| hsa-miR-301a | NAV2-AS4 |
| hsa-miR-454 | NAV2-AS4 |
| hsa-miR-205 | NAV2-AS4 |
| hsa-miR-23a | NAV2-AS4 |
| hsa-miR-27a | NAV2-AS4 |
| hsa-miR-10b | NAV2-AS4 |
| hsa-miR-424 | AC068858.1.1 |
| hsa-miR-211 | AC068858.1.1 |
| hsa-miR-211 | AC068858.1.1 |
| hsa-miR-211 | AC068858.1.1 |
| hsa-miR-205 | AC068858.1.1 |
| hsa-miR-122 | AC068858.1.1 |
| hsa-miR-92b | AC068858.1.1 |
| hsa-miR-27a | AC068858.1.1 |
| hsa-miR-27a | AC068858.1.1 |
| hsa-miR-338 | AC068858.1.1 |
| hsa-miR-10b | AC068858.1.1 |
| hsa-miR-424 | AC022182.1.1 |
| hsa-miR-424 | AC022182.1.1 |
| hsa-miR-183 | AC022182.1.1 |
| hsa-miR-211 | AC022182.1.1 |
| hsa-miR-21 | AC022182.1.1 |
| hsa-miR-23a | AC022182.1.1 |
| hsa-miR-23a | AC022182.1.1 |
| hsa-miR-338 | AC022182.1.1 |
| hsa-miR-383 | AC022182.1.1 |
| hsa-miR-141 | FAM66D |
| hsa-miR-200a | FAM66D |
| hsa-miR-181c | FAM66D |
| hsa-miR-181d | FAM66D |
| hsa-miR-187 | FAM66D |
| hsa-miR-205 | FAM66D |
| hsa-miR-221 | FAM66D |
| hsa-miR-222 | FAM66D |
| hsa-miR-23a | FAM66D |
| hsa-miR-27a | FAM66D |
| hsa-miR-96 | GRM5-AS1 |
| hsa-miR-96 | GRM5-AS1 |
| hsa-miR-144 | GRM5-AS1 |
| hsa-miR-155 | GRM5-AS1 |
| hsa-miR-181c | GRM5-AS1 |
| hsa-miR-181d | GRM5-AS1 |
| hsa-miR-182 | GRM5-AS1 |
| hsa-miR-182 | GRM5-AS1 |
| hsa-miR-182 | GRM5-AS1 |
| hsa-miR-196b | GRM5-AS1 |
| hsa-miR-200b | GRM5-AS1 |
| hsa-miR-200c | GRM5-AS1 |
| hsa-miR-429 | GRM5-AS1 |
| hsa-miR-200b | GRM5-AS1 |
| hsa-miR-200c | GRM5-AS1 |
| hsa-miR-429 | GRM5-AS1 |
| hsa-miR-205 | GRM5-AS1 |
| hsa-miR-21 | GRM5-AS1 |
| hsa-miR-23a | GRM5-AS1 |
| hsa-miR-23a | GRM5-AS1 |
| hsa-miR-23a | GRM5-AS1 |
| hsa-miR-31 | GRM5-AS1 |
| hsa-miR-338 | GRM5-AS1 |
| hsa-miR-338 | GRM5-AS1 |
| hsa-miR-33b | GRM5-AS1 |
| hsa-miR-33b | GRM5-AS1 |
| hsa-miR-33b | GRM5-AS1 |
| hsa-miR-383 | GRM5-AS1 |
| hsa-miR-137 | SNHG1 |
| hsa-miR-141 | SNHG1 |
| hsa-miR-200a | SNHG1 |
| hsa-miR-144 | SNHG1 |
| hsa-miR-424 | SNHG1 |
| hsa-miR-424 | SNHG1 |
| hsa-miR-181c | SNHG1 |
| hsa-miR-181d | SNHG1 |
| hsa-miR-182 | SNHG1 |
| hsa-miR-211 | SNHG1 |
| hsa-miR-205 | SNHG1 |
| hsa-miR-205 | SNHG1 |
| hsa-miR-21 | SNHG1 |
| hsa-miR-21 | SNHG1 |
| hsa-miR-122 | SNHG1 |
| hsa-miR-122 | SNHG1 |
| hsa-miR-23a | SNHG1 |
| hsa-miR-92b | SNHG1 |
| hsa-miR-383 | SNHG1 |
| hsa-miR-205 | AP000439.3.1 |
| hsa-miR-22 | AP000439.3.1 |
| hsa-miR-23a | AP000439.3.1 |
| hsa-miR-301a | KCNQ1OT1 |
| hsa-miR-454 | KCNQ1OT1 |
| hsa-miR-96 | KCNQ1OT1 |
| hsa-miR-96 | KCNQ1OT1 |
| hsa-miR-96 | KCNQ1OT1 |
| hsa-miR-135b | KCNQ1OT1 |
| hsa-miR-135b | KCNQ1OT1 |
| hsa-miR-137 | KCNQ1OT1 |
| hsa-miR-137 | KCNQ1OT1 |
| hsa-miR-137 | KCNQ1OT1 |
| hsa-miR-141 | KCNQ1OT1 |
| hsa-miR-200a | KCNQ1OT1 |
| hsa-miR-141 | KCNQ1OT1 |
| hsa-miR-200a | KCNQ1OT1 |
| hsa-miR-141 | KCNQ1OT1 |
| hsa-miR-200a | KCNQ1OT1 |
| hsa-miR-141 | KCNQ1OT1 |
| hsa-miR-200a | KCNQ1OT1 |
| hsa-miR-141 | KCNQ1OT1 |
| hsa-miR-200a | KCNQ1OT1 |
| hsa-miR-152 | KCNQ1OT1 |
| hsa-miR-152 | KCNQ1OT1 |
| hsa-miR-152 | KCNQ1OT1 |
| hsa-miR-152 | KCNQ1OT1 |
| hsa-miR-155 | KCNQ1OT1 |
| hsa-miR-155 | KCNQ1OT1 |
| hsa-miR-155 | KCNQ1OT1 |
| hsa-miR-155 | KCNQ1OT1 |
| hsa-miR-424 | KCNQ1OT1 |
| hsa-miR-424 | KCNQ1OT1 |
| hsa-miR-424 | KCNQ1OT1 |
| hsa-miR-181c | KCNQ1OT1 |
| hsa-miR-181d | KCNQ1OT1 |
| hsa-miR-181c | KCNQ1OT1 |
| hsa-miR-181d | KCNQ1OT1 |
| hsa-miR-181c | KCNQ1OT1 |
| hsa-miR-181d | KCNQ1OT1 |
| hsa-miR-181c | KCNQ1OT1 |
| hsa-miR-181d | KCNQ1OT1 |
| hsa-miR-182 | KCNQ1OT1 |
| hsa-miR-182 | KCNQ1OT1 |
| hsa-miR-182 | KCNQ1OT1 |
| hsa-miR-182 | KCNQ1OT1 |
| hsa-miR-182 | KCNQ1OT1 |
| hsa-miR-183 | KCNQ1OT1 |
| hsa-miR-183 | KCNQ1OT1 |
| hsa-miR-183 | KCNQ1OT1 |
| hsa-miR-183 | KCNQ1OT1 |
| hsa-miR-183 | KCNQ1OT1 |
| hsa-miR-187 | KCNQ1OT1 |
| hsa-miR-187 | KCNQ1OT1 |
| hsa-miR-192 | KCNQ1OT1 |
| hsa-miR-196b | KCNQ1OT1 |
| hsa-miR-196b | KCNQ1OT1 |
| hsa-miR-196b | KCNQ1OT1 |
| hsa-miR-200b | KCNQ1OT1 |
| hsa-miR-200c | KCNQ1OT1 |
| hsa-miR-429 | KCNQ1OT1 |
| hsa-miR-200b | KCNQ1OT1 |
| hsa-miR-200c | KCNQ1OT1 |
| hsa-miR-429 | KCNQ1OT1 |
| hsa-miR-211 | KCNQ1OT1 |
| hsa-miR-211 | KCNQ1OT1 |
| hsa-miR-211 | KCNQ1OT1 |
| hsa-miR-211 | KCNQ1OT1 |
| hsa-miR-211 | KCNQ1OT1 |
| hsa-miR-205 | KCNQ1OT1 |
| hsa-miR-205 | KCNQ1OT1 |
| hsa-miR-205 | KCNQ1OT1 |
| hsa-miR-22 | KCNQ1OT1 |
| hsa-miR-22 | KCNQ1OT1 |
| hsa-miR-22 | KCNQ1OT1 |
| hsa-miR-22 | KCNQ1OT1 |
| hsa-miR-221 | KCNQ1OT1 |
| hsa-miR-222 | KCNQ1OT1 |
| hsa-miR-221 | KCNQ1OT1 |
| hsa-miR-222 | KCNQ1OT1 |
| hsa-miR-221 | KCNQ1OT1 |
| hsa-miR-222 | KCNQ1OT1 |
| hsa-miR-221 | KCNQ1OT1 |
| hsa-miR-222 | KCNQ1OT1 |
| hsa-miR-122 | KCNQ1OT1 |
| hsa-miR-122 | KCNQ1OT1 |
| hsa-miR-122 | KCNQ1OT1 |
| hsa-miR-23a | KCNQ1OT1 |
| hsa-miR-23a | KCNQ1OT1 |
| hsa-miR-23a | KCNQ1OT1 |
| hsa-miR-23a | KCNQ1OT1 |
| hsa-miR-23a | KCNQ1OT1 |
| hsa-miR-92b | KCNQ1OT1 |
| hsa-miR-92b | KCNQ1OT1 |
| hsa-miR-92b | KCNQ1OT1 |
| hsa-miR-92b | KCNQ1OT1 |
| hsa-miR-92b | KCNQ1OT1 |
| hsa-miR-27a | KCNQ1OT1 |
| hsa-miR-27a | KCNQ1OT1 |
| hsa-miR-27a | KCNQ1OT1 |
| hsa-miR-27a | KCNQ1OT1 |
| hsa-miR-30a | KCNQ1OT1 |
| hsa-miR-30b | KCNQ1OT1 |
| hsa-miR-30d | KCNQ1OT1 |
| hsa-miR-338 | KCNQ1OT1 |
| hsa-miR-338 | KCNQ1OT1 |
| hsa-miR-338 | KCNQ1OT1 |
| hsa-miR-338 | KCNQ1OT1 |
| hsa-miR-338 | KCNQ1OT1 |
| hsa-miR-338 | KCNQ1OT1 |
| hsa-miR-338 | KCNQ1OT1 |
| hsa-miR-33b | KCNQ1OT1 |
| hsa-miR-33b | KCNQ1OT1 |
| hsa-miR-33b | KCNQ1OT1 |
| hsa-miR-33b | KCNQ1OT1 |
| hsa-miR-33b | KCNQ1OT1 |
| hsa-miR-34c | KCNQ1OT1 |
| hsa-miR-34c | KCNQ1OT1 |
| hsa-miR-10b | KCNQ1OT1 |
| hsa-miR-10b | KCNQ1OT1 |
| hsa-miR-22 | LINC00519 |
| hsa-miR-34c | LINC00519 |
| hsa-miR-137 | LINC00517 |
| hsa-miR-141 | LINC00517 |
| hsa-miR-200a | LINC00517 |
| hsa-miR-141 | LINC00517 |
| hsa-miR-200a | LINC00517 |
| hsa-miR-141 | LINC00517 |
| hsa-miR-200a | LINC00517 |
| hsa-miR-182 | LINC00517 |
| hsa-miR-183 | LINC00517 |
| hsa-miR-192 | LINC00517 |
| hsa-miR-192 | LINC00517 |
| hsa-miR-196b | LINC00517 |
| hsa-miR-200b | LINC00517 |
| hsa-miR-200c | LINC00517 |
| hsa-miR-429 | LINC00517 |
| hsa-miR-221 | LINC00517 |
| hsa-miR-222 | LINC00517 |
| hsa-miR-221 | LINC00517 |
| hsa-miR-222 | LINC00517 |
| hsa-miR-221 | LINC00517 |
| hsa-miR-222 | LINC00517 |
| hsa-miR-122 | LINC00517 |
| hsa-miR-23a | LINC00517 |
| hsa-miR-31 | LINC00517 |
| hsa-miR-338 | LINC00517 |
| hsa-miR-33b | LINC00517 |
| hsa-miR-383 | LINC00517 |
| hsa-miR-383 | LINC00517 |
| hsa-miR-10b | LINC00517 |
| hsa-miR-135b | AC009120.6.1 |
| hsa-miR-182 | AC009120.6.1 |
| hsa-miR-211 | AC009120.6.1 |
| hsa-miR-211 | AC009120.6.1 |
| hsa-miR-22 | AC009120.6.1 |
| hsa-miR-22 | AC009120.6.1 |
| hsa-miR-31 | AC009120.6.1 |
| hsa-miR-10b | AC009120.6.1 |
| hsa-miR-122 | AP001063.1.1 |
| hsa-miR-135b | FBXL19-AS1 |
| hsa-miR-141 | FBXL19-AS1 |
| hsa-miR-200a | FBXL19-AS1 |
| hsa-miR-141 | FBXL19-AS1 |
| hsa-miR-200a | FBXL19-AS1 |
| hsa-miR-152 | FBXL19-AS1 |
| hsa-miR-196b | FBXL19-AS1 |
| hsa-miR-22 | FBXL19-AS1 |
| hsa-miR-122 | FBXL19-AS1 |
| hsa-miR-122 | FBXL19-AS1 |
| hsa-miR-23a | FBXL19-AS1 |
| hsa-miR-135b | AC009120.4.1 |
| hsa-miR-182 | AC009120.4.1 |
| hsa-miR-192 | AC009120.4.1 |
| hsa-miR-27a | AC009120.4.1 |
| hsa-miR-31 | AC009120.4.1 |
| hsa-miR-10b | AC009120.4.1 |

| **Table S2. Details of DEmRNAs-DEmiRNAs pairs.** | |
| --- | --- |
| miRNA | mRNA |
| hsa-miR-10b | ABHD11 |
| hsa-miR-10b | ACAD10 |
| hsa-miR-10b | ACAD11 |
| hsa-miR-10b | ADCY7 |
| hsa-miR-10b | AFAP1L2 |
| hsa-miR-10b | AFMID |
| hsa-miR-10b | ALDH1L1 |
| hsa-miR-10b | ALPL |
| hsa-miR-10b | ANK1 |
| hsa-miR-10b | ANKRD29 |
| hsa-miR-10b | ANXA13 |
| hsa-miR-10b | ANXA4 |
| hsa-miR-10b | BDH1 |
| hsa-miR-10b | C15orf39 |
| hsa-miR-10b | CADM1 |
| hsa-miR-10b | CCDC40 |
| hsa-miR-10b | CHST4 |
| hsa-miR-10b | CNOT6 |
| hsa-miR-10b | COL24A1 |
| hsa-miR-10b | CS |
| hsa-miR-10b | CTBS |
| hsa-miR-10b | CYP24A1 |
| hsa-miR-10b | CYP2S1 |
| hsa-miR-10b | DNAL4 |
| hsa-miR-10b | ECHDC2 |
| hsa-miR-10b | ELAVL2 |
| hsa-miR-10b | FAM57B |
| hsa-miR-10b | FHAD1 |
| hsa-miR-10b | FHL3 |
| hsa-miR-10b | FNDC3A |
| hsa-miR-10b | GAS2 |
| hsa-miR-10b | GDA |
| hsa-miR-10b | GEMIN8 |
| hsa-miR-10b | GINS3 |
| hsa-miR-10b | GRIN2A |
| hsa-miR-10b | HS6ST2 |
| hsa-miR-10b | IFT172 |
| hsa-miR-10b | IL1RAP |
| hsa-miR-10b | IQCE |
| hsa-miR-10b | ITSN1 |
| hsa-miR-10b | LRP1 |
| hsa-miR-10b | MAP2 |
| hsa-miR-10b | MAPKBP1 |
| hsa-miR-10b | MAPRE1 |
| hsa-miR-10b | MCCC2 |
| hsa-miR-10b | MCM5 |
| hsa-miR-10b | MTM1 |
| hsa-miR-10b | NDE1 |
| hsa-miR-10b | NEDD4 |
| hsa-miR-10b | OBSCN |
| hsa-miR-10b | PDE4A |
| hsa-miR-10b | PEA15 |
| hsa-miR-10b | PLCD3 |
| hsa-miR-10b | PLCXD2 |
| hsa-miR-10b | PLXNB3 |
| hsa-miR-10b | POMT2 |
| hsa-miR-10b | POPDC2 |
| hsa-miR-10b | PRKACA |
| hsa-miR-10b | PRKRIP1 |
| hsa-miR-10b | PRLR |
| hsa-miR-10b | PTK7 |
| hsa-miR-10b | RAB37 |
| hsa-miR-10b | RER1 |
| hsa-miR-10b | SAMD13 |
| hsa-miR-10b | SEL1L |
| hsa-miR-10b | 3-Sep |
| hsa-miR-10b | SERPINE1 |
| hsa-miR-10b | SLCO1A2 |
| hsa-miR-10b | SLCO2B1 |
| hsa-miR-10b | SMYD2 |
| hsa-miR-10b | SOCS7 |
| hsa-miR-10b | STK32B |
| hsa-miR-10b | STXBP4 |
| hsa-miR-10b | SYNE1 |
| hsa-miR-10b | TBC1D22B |
| hsa-miR-10b | TFRC |
| hsa-miR-10b | TINAGL1 |
| hsa-miR-10b | TMED3 |
| hsa-miR-10b | TMEM119 |
| hsa-miR-10b | TMEM97 |
| hsa-miR-10b | TMPRSS13 |
| hsa-miR-10b | TRIM36 |
| hsa-miR-10b | TTF2 |
| hsa-miR-10b | TXNRD2 |
| hsa-miR-10b | UGT3A1 |
| hsa-miR-10b | VASH2 |
| hsa-miR-10b | VDR |
| hsa-miR-10b | VNN1 |
| hsa-miR-10b | WISP3 |
| hsa-miR-10b | ZDHHC14 |
| hsa-miR-10b | ZFAND5 |
| hsa-miR-10b | ZNF365 |
| hsa-miR-10b | ZNF83 |
| hsa-miR-10b | ZWINT |
| hsa-miR-122 | ABHD11 |
| hsa-miR-122 | ABR |
| hsa-miR-122 | ADAM10 |
| hsa-miR-122 | ADAMTS10 |
| hsa-miR-122 | ADCY6 |
| hsa-miR-122 | ALDOA |
| hsa-miR-122 | ANKRD13B |
| hsa-miR-122 | ANTXR1 |
| hsa-miR-122 | APBA2 |
| hsa-miR-122 | ARHGAP22 |
| hsa-miR-122 | ARNT2 |
| hsa-miR-122 | ATP13A2 |
| hsa-miR-122 | BCAN |
| hsa-miR-122 | BPHL |
| hsa-miR-122 | C4orf32 |
| hsa-miR-122 | CACNB2 |
| hsa-miR-122 | CASP2 |
| hsa-miR-122 | CCNJL |
| hsa-miR-122 | CDA |
| hsa-miR-122 | CEP55 |
| hsa-miR-122 | CES2 |
| hsa-miR-122 | CHRD |
| hsa-miR-122 | CRHR1 |
| hsa-miR-122 | CUBN |
| hsa-miR-122 | CYB561D1 |
| hsa-miR-122 | DCTN5 |
| hsa-miR-122 | DDX11 |
| hsa-miR-122 | DMRT2 |
| hsa-miR-122 | DNAJC12 |
| hsa-miR-122 | DNAJC18 |
| hsa-miR-122 | DUSP4 |
| hsa-miR-122 | DYSF |
| hsa-miR-122 | EGLN3 |
| hsa-miR-122 | ELMO1 |
| hsa-miR-122 | ERLIN2 |
| hsa-miR-122 | FAH |
| hsa-miR-122 | FCAMR |
| hsa-miR-122 | FKBP5 |
| hsa-miR-122 | FOSL2 |
| hsa-miR-122 | FSCN1 |
| hsa-miR-122 | G6PC3 |
| hsa-miR-122 | GALNT10 |
| hsa-miR-122 | GALNT12 |
| hsa-miR-122 | GIPC2 |
| hsa-miR-122 | GIT1 |
| hsa-miR-122 | GJB3 |
| hsa-miR-122 | GPAM |
| hsa-miR-122 | GPD1L |
| hsa-miR-122 | GPD2 |
| hsa-miR-122 | GPLD1 |
| hsa-miR-122 | GPM6B |
| hsa-miR-122 | GPRC5C |
| hsa-miR-122 | GRHL2 |
| hsa-miR-122 | GTSE1 |
| hsa-miR-122 | HADH |
| hsa-miR-122 | HHIP |
| hsa-miR-122 | HIP1R |
| hsa-miR-122 | HSF4 |
| hsa-miR-122 | HTR3A |
| hsa-miR-122 | IL22RA2 |
| hsa-miR-122 | ITGA11 |
| hsa-miR-122 | KALRN |
| hsa-miR-122 | KIF11 |
| hsa-miR-122 | KIFC2 |
| hsa-miR-122 | LDHD |
| hsa-miR-122 | LGR6 |
| hsa-miR-122 | LOXL4 |
| hsa-miR-122 | LRP10 |
| hsa-miR-122 | LYPD6 |
| hsa-miR-122 | MAP3K12 |
| hsa-miR-122 | MARK1 |
| hsa-miR-122 | MASP1 |
| hsa-miR-122 | MAST2 |
| hsa-miR-122 | MCOLN2 |
| hsa-miR-122 | MCTP2 |
| hsa-miR-122 | MDM4 |
| hsa-miR-122 | MEIS2 |
| hsa-miR-122 | METTL9 |
| hsa-miR-122 | MTM1 |
| hsa-miR-122 | NEB |
| hsa-miR-122 | NLGN3 |
| hsa-miR-122 | NPC1L1 |
| hsa-miR-122 | NT5E |
| hsa-miR-122 | OLR1 |
| hsa-miR-122 | OSBPL10 |
| hsa-miR-122 | PHF19 |
| hsa-miR-122 | PHYHD1 |
| hsa-miR-122 | PIGS |
| hsa-miR-122 | PLEKHB1 |
| hsa-miR-122 | POLR1E |
| hsa-miR-122 | POMT2 |
| hsa-miR-122 | PPFIBP2 |
| hsa-miR-122 | PRMT2 |
| hsa-miR-122 | PRTFDC1 |
| hsa-miR-122 | PYCARD |
| hsa-miR-122 | RASGEF1A |
| hsa-miR-122 | RBP5 |
| hsa-miR-122 | RCC2 |
| hsa-miR-122 | RCE1 |
| hsa-miR-122 | SAMD13 |
| hsa-miR-122 | SARM1 |
| hsa-miR-122 | SATB1 |
| hsa-miR-122 | SECTM1 |
| hsa-miR-122 | SELENBP1 |
| hsa-miR-122 | SEMA4A |
| hsa-miR-122 | 2-Sep |
| hsa-miR-122 | SLC10A3 |
| hsa-miR-122 | SLC13A5 |
| hsa-miR-122 | SLC41A1 |
| hsa-miR-122 | SLC9A1 |
| hsa-miR-122 | SMTN |
| hsa-miR-122 | SNTG2 |
| hsa-miR-122 | SOCS7 |
| hsa-miR-122 | SSH3 |
| hsa-miR-122 | ST6GALNAC4 |
| hsa-miR-122 | STX6 |
| hsa-miR-122 | STXBP2 |
| hsa-miR-122 | TCOF1 |
| hsa-miR-122 | TJP2 |
| hsa-miR-122 | TPD52L2 |
| hsa-miR-122 | TPST2 |
| hsa-miR-122 | TRAPPC4 |
| hsa-miR-122 | TRIM29 |
| hsa-miR-122 | TRPM1 |
| hsa-miR-122 | TTLL6 |
| hsa-miR-122 | UGT8 |
| hsa-miR-122 | UPK2 |
| hsa-miR-122 | VAV3 |
| hsa-miR-122 | VPS45 |
| hsa-miR-122 | WDR7 |
| hsa-miR-122 | WNT2B |
| hsa-miR-122 | YKT6 |
| hsa-miR-122 | ZNF365 |
| hsa-miR-122 | ZNF415 |
| hsa-miR-122 | ZNF589 |
| hsa-miR-122 | ZNF668 |
| hsa-miR-135b | ABCA1 |
| hsa-miR-135b | ABCA5 |
| hsa-miR-135b | ABCD3 |
| hsa-miR-135b | ABCG5 |
| hsa-miR-135b | ACAA2 |
| hsa-miR-135b | ADAMTS14 |
| hsa-miR-135b | ADARB2 |
| hsa-miR-135b | ADSS |
| hsa-miR-135b | AKR1A1 |
| hsa-miR-135b | ALDH1B1 |
| hsa-miR-135b | ANK1 |
| hsa-miR-135b | ANKRD46 |
| hsa-miR-135b | ANTXR1 |
| hsa-miR-135b | AP1G2 |
| hsa-miR-135b | ARHGAP20 |
| hsa-miR-135b | ARHGEF3 |
| hsa-miR-135b | ASL |
| hsa-miR-135b | ATP10B |
| hsa-miR-135b | ATP11C |
| hsa-miR-135b | B4GALNT1 |
| hsa-miR-135b | BACE1 |
| hsa-miR-135b | C17orf53 |
| hsa-miR-135b | CACNB1 |
| hsa-miR-135b | CD36 |
| hsa-miR-135b | CEP170 |
| hsa-miR-135b | CLSTN1 |
| hsa-miR-135b | CMBL |
| hsa-miR-135b | COL5A1 |
| hsa-miR-135b | DBF4B |
| hsa-miR-135b | DNAH5 |
| hsa-miR-135b | DNAJC16 |
| hsa-miR-135b | DOK1 |
| hsa-miR-135b | DOLPP1 |
| hsa-miR-135b | DRP2 |
| hsa-miR-135b | EDA |
| hsa-miR-135b | EDA2R |
| hsa-miR-135b | EDIL3 |
| hsa-miR-135b | EMILIN2 |
| hsa-miR-135b | F10 |
| hsa-miR-135b | FAH |
| hsa-miR-135b | FBLIM1 |
| hsa-miR-135b | FMNL1 |
| hsa-miR-135b | FMNL2 |
| hsa-miR-135b | FOSL2 |
| hsa-miR-135b | FPGS |
| hsa-miR-135b | FRMPD2 |
| hsa-miR-135b | FUT2 |
| hsa-miR-135b | GABPB2 |
| hsa-miR-135b | GCLC |
| hsa-miR-135b | GMNN |
| hsa-miR-135b | GPC6 |
| hsa-miR-135b | GPLD1 |
| hsa-miR-135b | GPR161 |
| hsa-miR-135b | GRIA4 |
| hsa-miR-135b | GRIP1 |
| hsa-miR-135b | HAPLN1 |
| hsa-miR-135b | HERC5 |
| hsa-miR-135b | HKDC1 |
| hsa-miR-135b | HPS5 |
| hsa-miR-135b | HSD17B12 |
| hsa-miR-135b | IDH1 |
| hsa-miR-135b | IQGAP3 |
| hsa-miR-135b | ITGB8 |
| hsa-miR-135b | KALRN |
| hsa-miR-135b | KCNAB3 |
| hsa-miR-135b | KCNS1 |
| hsa-miR-135b | KCTD10 |
| hsa-miR-135b | KIF21B |
| hsa-miR-135b | KLHL32 |
| hsa-miR-135b | LIMK2 |
| hsa-miR-135b | MAP2 |
| hsa-miR-135b | MARK1 |
| hsa-miR-135b | MASP1 |
| hsa-miR-135b | MCM5 |
| hsa-miR-135b | MCM8 |
| hsa-miR-135b | MDM4 |
| hsa-miR-135b | MEIS2 |
| hsa-miR-135b | MON1A |
| hsa-miR-135b | MTHFD2L |
| hsa-miR-135b | MTSS1 |
| hsa-miR-135b | MUT |
| hsa-miR-135b | MYO1C |
| hsa-miR-135b | NAGS |
| hsa-miR-135b | NDRG4 |
| hsa-miR-135b | NR3C2 |
| hsa-miR-135b | OLFML1 |
| hsa-miR-135b | PANK1 |
| hsa-miR-135b | PCYOX1L |
| hsa-miR-135b | PCYT1B |
| hsa-miR-135b | PDE2A |
| hsa-miR-135b | PHF19 |
| hsa-miR-135b | PHF21A |
| hsa-miR-135b | PNKD |
| hsa-miR-135b | POLD3 |
| hsa-miR-135b | PPFIBP2 |
| hsa-miR-135b | PRRX1 |
| hsa-miR-135b | RIC8B |
| hsa-miR-135b | RRBP1 |
| hsa-miR-135b | RUSC1 |
| hsa-miR-135b | SAMD4A |
| hsa-miR-135b | SCUBE3 |
| hsa-miR-135b | 8-Sep |
| hsa-miR-135b | SETD3 |
| hsa-miR-135b | SLC13A4 |
| hsa-miR-135b | SLC17A4 |
| hsa-miR-135b | SLC22A2 |
| hsa-miR-135b | SLC25A18 |
| hsa-miR-135b | SLC26A2 |
| hsa-miR-135b | SLC31A2 |
| hsa-miR-135b | SLC39A13 |
| hsa-miR-135b | SLC4A10 |
| hsa-miR-135b | SNRK |
| hsa-miR-135b | SNURF |
| hsa-miR-135b | SPATA18 |
| hsa-miR-135b | SPIRE1 |
| hsa-miR-135b | ST3GAL3 |
| hsa-miR-135b | STAB2 |
| hsa-miR-135b | SYK |
| hsa-miR-135b | SYNE1 |
| hsa-miR-135b | SYNGAP1 |
| hsa-miR-135b | TBX19 |
| hsa-miR-135b | THBS2 |
| hsa-miR-135b | THRB |
| hsa-miR-135b | TMED5 |
| hsa-miR-135b | TMEM163 |
| hsa-miR-135b | TP53I11 |
| hsa-miR-135b | TRMT11 |
| hsa-miR-135b | TRPC6 |
| hsa-miR-135b | TRPM4 |
| hsa-miR-135b | TSC22D2 |
| hsa-miR-135b | TTC21A |
| hsa-miR-135b | VNN1 |
| hsa-miR-135b | VPS45 |
| hsa-miR-135b | WDR7 |
| hsa-miR-135b | ZDHHC13 |
| hsa-miR-135b | ZFAND5 |
| hsa-miR-135b | ZFP1 |
| hsa-miR-135b | ZHX1 |
| hsa-miR-135b | ZNF135 |
| hsa-miR-135b | ZNF275 |
| hsa-miR-135b | ZNF365 |
| hsa-miR-135b | ZNF501 |
| hsa-miR-135b | ZNF607 |
| hsa-miR-137 | AACS |
| hsa-miR-137 | ABCC9 |
| hsa-miR-137 | ABHD5 |
| hsa-miR-137 | ABHD6 |
| hsa-miR-137 | ACTN2 |
| hsa-miR-137 | ADAM23 |
| hsa-miR-137 | AHR |
| hsa-miR-137 | ALDH8A1 |
| hsa-miR-137 | ANGPTL3 |
| hsa-miR-137 | ANKRD28 |
| hsa-miR-137 | ANO4 |
| hsa-miR-137 | APLN |
| hsa-miR-137 | APPL2 |
| hsa-miR-137 | ARHGAP42 |
| hsa-miR-137 | ARHGEF18 |
| hsa-miR-137 | ASH1L |
| hsa-miR-137 | ATP10A |
| hsa-miR-137 | ATP1B1 |
| hsa-miR-137 | ATP2B2 |
| hsa-miR-137 | ATPAF1 |
| hsa-miR-137 | B3GALNT2 |
| hsa-miR-137 | B3GALT2 |
| hsa-miR-137 | BAZ2A |
| hsa-miR-137 | BEND3 |
| hsa-miR-137 | BEND6 |
| hsa-miR-137 | C21orf91 |
| hsa-miR-137 | C2CD4C |
| hsa-miR-137 | C7orf31 |
| hsa-miR-137 | CACNA1D |
| hsa-miR-137 | CACNB2 |
| hsa-miR-137 | CADPS |
| hsa-miR-137 | CAPN2 |
| hsa-miR-137 | CCDC153 |
| hsa-miR-137 | CCNG2 |
| hsa-miR-137 | CDC37L1 |
| hsa-miR-137 | CDH11 |
| hsa-miR-137 | CDH20 |
| hsa-miR-137 | CHIC1 |
| hsa-miR-137 | CHST1 |
| hsa-miR-137 | CHST10 |
| hsa-miR-137 | CNTN3 |
| hsa-miR-137 | COL5A1 |
| hsa-miR-137 | CREM |
| hsa-miR-137 | CTNNA3 |
| hsa-miR-137 | CTSF |
| hsa-miR-137 | CTTNBP2 |
| hsa-miR-137 | CTTNBP2NL |
| hsa-miR-137 | CXCL12 |
| hsa-miR-137 | DBN1 |
| hsa-miR-137 | DCDC2 |
| hsa-miR-137 | DEXI |
| hsa-miR-137 | DGKG |
| hsa-miR-137 | DIRAS2 |
| hsa-miR-137 | DMRT2 |
| hsa-miR-137 | DUSP4 |
| hsa-miR-137 | E2F7 |
| hsa-miR-137 | EDIL3 |
| hsa-miR-137 | EFHC2 |
| hsa-miR-137 | EFNA3 |
| hsa-miR-137 | EHBP1L1 |
| hsa-miR-137 | ELFN2 |
| hsa-miR-137 | ELOVL1 |
| hsa-miR-137 | ELOVL2 |
| hsa-miR-137 | EPHA7 |
| hsa-miR-137 | ETFB |
| hsa-miR-137 | EXO1 |
| hsa-miR-137 | EZH2 |
| hsa-miR-137 | FAM117B |
| hsa-miR-137 | FAM135B |
| hsa-miR-137 | FAM160B2 |
| hsa-miR-137 | FAM167A |
| hsa-miR-137 | FAM81A |
| hsa-miR-137 | FBXL4 |
| hsa-miR-137 | FGB |
| hsa-miR-137 | FGF11 |
| hsa-miR-137 | FGL2 |
| hsa-miR-137 | FMNL2 |
| hsa-miR-137 | FNBP1L |
| hsa-miR-137 | FNDC5 |
| hsa-miR-137 | FURIN |
| hsa-miR-137 | GCA |
| hsa-miR-137 | GJC1 |
| hsa-miR-137 | GLIS2 |
| hsa-miR-137 | GNAT1 |
| hsa-miR-137 | GPAM |
| hsa-miR-137 | GPD2 |
| hsa-miR-137 | GPR88 |
| hsa-miR-137 | GPRC5A |
| hsa-miR-137 | GREB1 |
| hsa-miR-137 | GREM2 |
| hsa-miR-137 | GRIN2A |
| hsa-miR-137 | GRIP1 |
| hsa-miR-137 | GRM5 |
| hsa-miR-137 | GULP1 |
| hsa-miR-137 | HCFC2 |
| hsa-miR-137 | HERC3 |
| hsa-miR-137 | HEY2 |
| hsa-miR-137 | HK3 |
| hsa-miR-137 | HLF |
| hsa-miR-137 | HMGCLL1 |
| hsa-miR-137 | HOXC4 |
| hsa-miR-137 | HOXC8 |
| hsa-miR-137 | IDH1 |
| hsa-miR-137 | IGFBP5 |
| hsa-miR-137 | IMPA2 |
| hsa-miR-137 | ITGA9 |
| hsa-miR-137 | JAG1 |
| hsa-miR-137 | JAKMIP2 |
| hsa-miR-137 | JDP2 |
| hsa-miR-137 | KANK4 |
| hsa-miR-137 | KAT2B |
| hsa-miR-137 | KCND1 |
| hsa-miR-137 | KCND2 |
| hsa-miR-137 | KDELR3 |
| hsa-miR-137 | KDM5B |
| hsa-miR-137 | KIAA0907 |
| hsa-miR-137 | KIAA1024 |
| hsa-miR-137 | KLF11 |
| hsa-miR-137 | KLF15 |
| hsa-miR-137 | LAPTM4B |
| hsa-miR-137 | LIMCH1 |
| hsa-miR-137 | LONRF3 |
| hsa-miR-137 | LRP12 |
| hsa-miR-137 | LRP6 |
| hsa-miR-137 | LRRC10B |
| hsa-miR-137 | LRRC4 |
| hsa-miR-137 | LRRN3 |
| hsa-miR-137 | MAF |
| hsa-miR-137 | MAP3K1 |
| hsa-miR-137 | MARK1 |
| hsa-miR-137 | MBNL2 |
| hsa-miR-137 | MBOAT2 |
| hsa-miR-137 | MEGF9 |
| hsa-miR-137 | METTL9 |
| hsa-miR-137 | MGAT5B |
| hsa-miR-137 | MIA3 |
| hsa-miR-137 | MITF |
| hsa-miR-137 | MPP1 |
| hsa-miR-137 | MSRB3 |
| hsa-miR-137 | MTFR1 |
| hsa-miR-137 | MTMR4 |
| hsa-miR-137 | MTSS1 |
| hsa-miR-137 | MYO1C |
| hsa-miR-137 | NBEA |
| hsa-miR-137 | NCKAP5 |
| hsa-miR-137 | NDUFS1 |
| hsa-miR-137 | NETO1 |
| hsa-miR-137 | NFATC1 |
| hsa-miR-137 | NKAIN1 |
| hsa-miR-137 | NLGN4X |
| hsa-miR-137 | NOVA1 |
| hsa-miR-137 | NR1H4 |
| hsa-miR-137 | NT5DC2 |
| hsa-miR-137 | OSBP2 |
| hsa-miR-137 | OSBPL3 |
| hsa-miR-137 | PALM2-AKAP2 |
| hsa-miR-137 | PDE10A |
| hsa-miR-137 | PDE4A |
| hsa-miR-137 | PDE7A |
| hsa-miR-137 | PDHB |
| hsa-miR-137 | PEX3 |
| hsa-miR-137 | PIP5KL1 |
| hsa-miR-137 | PLCB1 |
| hsa-miR-137 | PPARGC1A |
| hsa-miR-137 | PRR16 |
| hsa-miR-137 | PTGFRN |
| hsa-miR-137 | PTPN5 |
| hsa-miR-137 | RAP2C |
| hsa-miR-137 | RASAL2 |
| hsa-miR-137 | RASGRF1 |
| hsa-miR-137 | RASSF8 |
| hsa-miR-137 | RAVER2 |
| hsa-miR-137 | RCOR2 |
| hsa-miR-137 | RELL2 |
| hsa-miR-137 | RFTN1 |
| hsa-miR-137 | RIC8B |
| hsa-miR-137 | RIMKLB |
| hsa-miR-137 | RIMS3 |
| hsa-miR-137 | RNF150 |
| hsa-miR-137 | RNF152 |
| hsa-miR-137 | RNF157 |
| hsa-miR-137 | RNF165 |
| hsa-miR-137 | RNF39 |
| hsa-miR-137 | RORA |
| hsa-miR-137 | RPGRIP1L |
| hsa-miR-137 | RYR2 |
| hsa-miR-137 | SAR1B |
| hsa-miR-137 | SCRT1 |
| hsa-miR-137 | SEMA4D |
| hsa-miR-137 | 3-Sep |
| hsa-miR-137 | SERPINA3 |
| hsa-miR-137 | SEZ6L2 |
| hsa-miR-137 | SH3BP5 |
| hsa-miR-137 | SHISA9 |
| hsa-miR-137 | SIK1 |
| hsa-miR-137 | SIK2 |
| hsa-miR-137 | SIPA1L2 |
| hsa-miR-137 | SIPA1L3 |
| hsa-miR-137 | SIRT6 |
| hsa-miR-137 | SLAIN1 |
| hsa-miR-137 | SLC12A2 |
| hsa-miR-137 | SLC1A5 |
| hsa-miR-137 | SLC24A3 |
| hsa-miR-137 | SLC30A4 |
| hsa-miR-137 | SLC39A13 |
| hsa-miR-137 | SLC43A2 |
| hsa-miR-137 | SLC46A3 |
| hsa-miR-137 | SLC6A1 |
| hsa-miR-137 | SLC6A8 |
| hsa-miR-137 | SLC6A9 |
| hsa-miR-137 | SLC9A3R2 |
| hsa-miR-137 | SNAP91 |
| hsa-miR-137 | SNRK |
| hsa-miR-137 | SOCS6 |
| hsa-miR-137 | SOX11 |
| hsa-miR-137 | SPATS2 |
| hsa-miR-137 | SRC |
| hsa-miR-137 | SSFA2 |
| hsa-miR-137 | ST13 |
| hsa-miR-137 | ST3GAL3 |
| hsa-miR-137 | STC1 |
| hsa-miR-137 | STC2 |
| hsa-miR-137 | SULF1 |
| hsa-miR-137 | SV2A |
| hsa-miR-137 | TBC1D19 |
| hsa-miR-137 | TBX15 |
| hsa-miR-137 | TCF3 |
| hsa-miR-137 | TESK2 |
| hsa-miR-137 | TFAP2A |
| hsa-miR-137 | TFAP2C |
| hsa-miR-137 | TGFA |
| hsa-miR-137 | TMED5 |
| hsa-miR-137 | TMEM132B |
| hsa-miR-137 | TMEM229B |
| hsa-miR-137 | TMEM55A |
| hsa-miR-137 | TMEM56 |
| hsa-miR-137 | TMEM87B |
| hsa-miR-137 | TMSB15B |
| hsa-miR-137 | TNC |
| hsa-miR-137 | TNXB |
| hsa-miR-137 | TRMT6 |
| hsa-miR-137 | TSC22D2 |
| hsa-miR-137 | TTC26 |
| hsa-miR-137 | TTC6 |
| hsa-miR-137 | TWIST1 |
| hsa-miR-137 | UGP2 |
| hsa-miR-137 | USP30 |
| hsa-miR-137 | USP38 |
| hsa-miR-137 | VASH2 |
| hsa-miR-137 | VKORC1 |
| hsa-miR-137 | WIF1 |
| hsa-miR-137 | WNT7A |
| hsa-miR-137 | ZBTB4 |
| hsa-miR-137 | ZCCHC2 |
| hsa-miR-137 | ZMAT4 |
| hsa-miR-137 | ZNF385B |
| hsa-miR-137 | ZNF654 |
| hsa-miR-137 | ZNF770 |
| hsa-miR-137 | ZNF793 |
| hsa-miR-137 | ZSWIM4 |
| hsa-miR-141 | ABCD4 |
| hsa-miR-141 | ABCG2 |
| hsa-miR-141 | ABHD10 |
| hsa-miR-141 | ACAD11 |
| hsa-miR-141 | ACAD8 |
| hsa-miR-141 | ACP2 |
| hsa-miR-141 | ADD2 |
| hsa-miR-141 | AGT |
| hsa-miR-141 | ALB |
| hsa-miR-141 | ALDH1L1 |
| hsa-miR-141 | ALDH1L2 |
| hsa-miR-141 | ANTXR1 |
| hsa-miR-141 | ARHGEF18 |
| hsa-miR-141 | ASB13 |
| hsa-miR-141 | ATP11C |
| hsa-miR-141 | BAG2 |
| hsa-miR-141 | BEST3 |
| hsa-miR-141 | BRSK2 |
| hsa-miR-141 | C11orf54 |
| hsa-miR-141 | C19orf44 |
| hsa-miR-141 | C1RL |
| hsa-miR-141 | C20orf96 |
| hsa-miR-141 | C9orf72 |
| hsa-miR-141 | CD200 |
| hsa-miR-141 | CD80 |
| hsa-miR-141 | CDS1 |
| hsa-miR-141 | CLPB |
| hsa-miR-141 | CNBP |
| hsa-miR-141 | COL24A1 |
| hsa-miR-141 | CPEB4 |
| hsa-miR-141 | CRIP2 |
| hsa-miR-141 | CRMP1 |
| hsa-miR-141 | CSNK1E |
| hsa-miR-141 | CTBP2 |
| hsa-miR-141 | CTNND2 |
| hsa-miR-141 | CTTNBP2 |
| hsa-miR-141 | CYP3A43 |
| hsa-miR-141 | DLG3 |
| hsa-miR-141 | DMWD |
| hsa-miR-141 | DNAJC6 |
| hsa-miR-141 | DOLPP1 |
| hsa-miR-141 | DTL |
| hsa-miR-141 | DYNLRB2 |
| hsa-miR-141 | DYRK2 |
| hsa-miR-141 | E2F3 |
| hsa-miR-141 | ELAVL2 |
| hsa-miR-141 | EME1 |
| hsa-miR-141 | ENPP5 |
| hsa-miR-141 | FBLN5 |
| hsa-miR-141 | FGB |
| hsa-miR-141 | FOSB |
| hsa-miR-141 | FRMD4A |
| hsa-miR-141 | FST |
| hsa-miR-141 | GLRX |
| hsa-miR-141 | GLYAT |
| hsa-miR-141 | GON4L |
| hsa-miR-141 | GPAM |
| hsa-miR-141 | GPHN |
| hsa-miR-141 | GPM6B |
| hsa-miR-141 | GPR160 |
| hsa-miR-141 | GRHL1 |
| hsa-miR-141 | HCFC2 |
| hsa-miR-141 | HIP1R |
| hsa-miR-141 | HNMT |
| hsa-miR-141 | HPS5 |
| hsa-miR-141 | IGSF11 |
| hsa-miR-141 | IL20RA |
| hsa-miR-141 | ISLR2 |
| hsa-miR-141 | ITGA11 |
| hsa-miR-141 | ITGA3 |
| hsa-miR-141 | ITGA6 |
| hsa-miR-141 | KCNH8 |
| hsa-miR-141 | KCNIP2 |
| hsa-miR-141 | KCNQ5 |
| hsa-miR-141 | KCNS1 |
| hsa-miR-141 | KDR |
| hsa-miR-141 | KIAA1841 |
| hsa-miR-141 | KLHDC2 |
| hsa-miR-141 | LAMC2 |
| hsa-miR-141 | LGR4 |
| hsa-miR-141 | LLGL1 |
| hsa-miR-141 | LPIN1 |
| hsa-miR-141 | LRIG1 |
| hsa-miR-141 | LRP12 |
| hsa-miR-141 | LYPD6 |
| hsa-miR-141 | MAK |
| hsa-miR-141 | MAN1C1 |
| hsa-miR-141 | MAPK8IP3 |
| hsa-miR-141 | MARK1 |
| hsa-miR-141 | MCM3 |
| hsa-miR-141 | MDM4 |
| hsa-miR-141 | METTL9 |
| hsa-miR-141 | MFAP3L |
| hsa-miR-141 | MPZL1 |
| hsa-miR-141 | MTMR10 |
| hsa-miR-141 | MTO1 |
| hsa-miR-141 | MTSS1 |
| hsa-miR-141 | MVP |
| hsa-miR-141 | MYRIP |
| hsa-miR-141 | NADK |
| hsa-miR-141 | NCAN |
| hsa-miR-141 | NEK11 |
| hsa-miR-141 | NOVA1 |
| hsa-miR-141 | NRCAM |
| hsa-miR-141 | NRSN2 |
| hsa-miR-141 | NUDT12 |
| hsa-miR-141 | OBSCN |
| hsa-miR-141 | OR51E1 |
| hsa-miR-141 | PAK6 |
| hsa-miR-141 | PAM |
| hsa-miR-141 | PAQR5 |
| hsa-miR-141 | PCK1 |
| hsa-miR-141 | PDGFD |
| hsa-miR-141 | PIAS3 |
| hsa-miR-141 | PKP2 |
| hsa-miR-141 | PLCB1 |
| hsa-miR-141 | PLEKHH2 |
| hsa-miR-141 | PMAIP1 |
| hsa-miR-141 | POGK |
| hsa-miR-141 | PON1 |
| hsa-miR-141 | PRDM4 |
| hsa-miR-141 | PRKCE |
| hsa-miR-141 | PTPN5 |
| hsa-miR-141 | RAB27B |
| hsa-miR-141 | RAB38 |
| hsa-miR-141 | RAD23B |
| hsa-miR-141 | RALGPS1 |
| hsa-miR-141 | RAP2C |
| hsa-miR-141 | RBPMS |
| hsa-miR-141 | RER1 |
| hsa-miR-141 | RIMS2 |
| hsa-miR-141 | SACM1L |
| hsa-miR-141 | SCARA5 |
| hsa-miR-141 | SCD5 |
| hsa-miR-141 | SCIN |
| hsa-miR-141 | SDC2 |
| hsa-miR-141 | SEMA4A |
| hsa-miR-141 | 2-Sep |
| hsa-miR-141 | 8-Sep |
| hsa-miR-141 | SH3BP5 |
| hsa-miR-141 | SH3TC2 |
| hsa-miR-141 | SIGLEC1 |
| hsa-miR-141 | SIPA1L2 |
| hsa-miR-141 | SLC13A3 |
| hsa-miR-141 | SLC16A3 |
| hsa-miR-141 | SLC19A2 |
| hsa-miR-141 | SLC25A16 |
| hsa-miR-141 | SLC30A10 |
| hsa-miR-141 | SLC31A1 |
| hsa-miR-141 | SLC38A2 |
| hsa-miR-141 | SLC38A4 |
| hsa-miR-141 | SLC6A12 |
| hsa-miR-141 | SMYD2 |
| hsa-miR-141 | SNRK |
| hsa-miR-141 | SNRPD1 |
| hsa-miR-141 | SNX4 |
| hsa-miR-141 | SPATA20 |
| hsa-miR-141 | SPATS2 |
| hsa-miR-141 | ST3GAL3 |
| hsa-miR-141 | ST3GAL6 |
| hsa-miR-141 | STK32B |
| hsa-miR-141 | SYT6 |
| hsa-miR-141 | SYT9 |
| hsa-miR-141 | SYTL4 |
| hsa-miR-141 | SYTL5 |
| hsa-miR-141 | TACC3 |
| hsa-miR-141 | TCERG1 |
| hsa-miR-141 | TDRD6 |
| hsa-miR-141 | TF |
| hsa-miR-141 | TFRC |
| hsa-miR-141 | THSD4 |
| hsa-miR-141 | TM4SF1 |
| hsa-miR-141 | TMCC2 |
| hsa-miR-141 | TMEM100 |
| hsa-miR-141 | TMEM130 |
| hsa-miR-141 | TMEM26 |
| hsa-miR-141 | TP73 |
| hsa-miR-141 | TPM1 |
| hsa-miR-141 | TTC7B |
| hsa-miR-141 | TTLL2 |
| hsa-miR-141 | TTR |
| hsa-miR-141 | TXNIP |
| hsa-miR-141 | UBE2Q2 |
| hsa-miR-141 | UGP2 |
| hsa-miR-141 | UGT8 |
| hsa-miR-141 | VASH2 |
| hsa-miR-141 | VAV3 |
| hsa-miR-141 | VCAN |
| hsa-miR-141 | VLDLR |
| hsa-miR-141 | VSX1 |
| hsa-miR-141 | ZFP1 |
| hsa-miR-141 | ZNF528 |
| hsa-miR-141 | ZNF608 |
| hsa-miR-141 | ZNF618 |
| hsa-miR-141 | ZNF793 |
| hsa-miR-141 | ZSCAN23 |
| hsa-miR-144 | ABCA5 |
| hsa-miR-144 | ABCD3 |
| hsa-miR-144 | CD160 |
| hsa-miR-144 | CD302 |
| hsa-miR-144 | DCLRE1C |
| hsa-miR-144 | KCNT2 |
| hsa-miR-144 | KCTD10 |
| hsa-miR-144 | MTSS1 |
| hsa-miR-144 | PLOD2 |
| hsa-miR-144 | RAB27A |
| hsa-miR-144 | SACS |
| hsa-miR-144 | SLC22A3 |
| hsa-miR-144 | SMARCA2 |
| hsa-miR-144 | SPIRE1 |
| hsa-miR-144 | SYNE1 |
| hsa-miR-144 | THNSL1 |
| hsa-miR-144 | TJP2 |
| hsa-miR-144 | WDR7 |
| hsa-miR-144 | ZNF528 |
| hsa-miR-152 | ABCB5 |
| hsa-miR-152 | ABCC5 |
| hsa-miR-152 | ADARB2 |
| hsa-miR-152 | AGBL2 |
| hsa-miR-152 | ANKDD1A |
| hsa-miR-152 | APBB1 |
| hsa-miR-152 | C16orf70 |
| hsa-miR-152 | C5orf30 |
| hsa-miR-152 | CAPN7 |
| hsa-miR-152 | CD4 |
| hsa-miR-152 | CD58 |
| hsa-miR-152 | CETN2 |
| hsa-miR-152 | CKAP2 |
| hsa-miR-152 | CNTNAP5 |
| hsa-miR-152 | COL4A1 |
| hsa-miR-152 | CRY2 |
| hsa-miR-152 | DENND2C |
| hsa-miR-152 | DGCR2 |
| hsa-miR-152 | DKK2 |
| hsa-miR-152 | DKK3 |
| hsa-miR-152 | DNAH9 |
| hsa-miR-152 | DNMT1 |
| hsa-miR-152 | DNMT3B |
| hsa-miR-152 | DRP2 |
| hsa-miR-152 | DYRK2 |
| hsa-miR-152 | ECEL1 |
| hsa-miR-152 | ELMO1 |
| hsa-miR-152 | EPM2A |
| hsa-miR-152 | FAH |
| hsa-miR-152 | FAM81A |
| hsa-miR-152 | FZD6 |
| hsa-miR-152 | GCH1 |
| hsa-miR-152 | GOLT1A |
| hsa-miR-152 | GPHN |
| hsa-miR-152 | GPM6A |
| hsa-miR-152 | GPRC5B |
| hsa-miR-152 | GRAMD1C |
| hsa-miR-152 | HLF |
| hsa-miR-152 | INTS6 |
| hsa-miR-152 | ITGA5 |
| hsa-miR-152 | ITGA6 |
| hsa-miR-152 | LAMA3 |
| hsa-miR-152 | LRP12 |
| hsa-miR-152 | MIF4GD |
| hsa-miR-152 | MMAA |
| hsa-miR-152 | MPDZ |
| hsa-miR-152 | MRPS25 |
| hsa-miR-152 | MTA3 |
| hsa-miR-152 | MTMR4 |
| hsa-miR-152 | MYO5C |
| hsa-miR-152 | NID1 |
| hsa-miR-152 | NPEPL1 |
| hsa-miR-152 | NR1H4 |
| hsa-miR-152 | NUDT12 |
| hsa-miR-152 | ORAOV1 |
| hsa-miR-152 | PAPPA2 |
| hsa-miR-152 | PAQR8 |
| hsa-miR-152 | PCYT1B |
| hsa-miR-152 | PDE1C |
| hsa-miR-152 | PDE2A |
| hsa-miR-152 | PDLIM5 |
| hsa-miR-152 | PGRMC1 |
| hsa-miR-152 | PKHD1 |
| hsa-miR-152 | PLA2G4F |
| hsa-miR-152 | PPARG |
| hsa-miR-152 | PPP2R1B |
| hsa-miR-152 | PRKG2 |
| hsa-miR-152 | PTPRN |
| hsa-miR-152 | RAD51 |
| hsa-miR-152 | RASSF8 |
| hsa-miR-152 | RHBDF2 |
| hsa-miR-152 | RNF152 |
| hsa-miR-152 | RUFY2 |
| hsa-miR-152 | SACS |
| hsa-miR-152 | SH3TC2 |
| hsa-miR-152 | SLC13A4 |
| hsa-miR-152 | SLC22A5 |
| hsa-miR-152 | SLC25A29 |
| hsa-miR-152 | SLC4A10 |
| hsa-miR-152 | SLCO2B1 |
| hsa-miR-152 | SNRK |
| hsa-miR-152 | SPATS2 |
| hsa-miR-152 | SPIRE1 |
| hsa-miR-152 | SSFA2 |
| hsa-miR-152 | STX6 |
| hsa-miR-152 | SULT1C2 |
| hsa-miR-152 | SYT10 |
| hsa-miR-152 | SYTL5 |
| hsa-miR-152 | TACC2 |
| hsa-miR-152 | TINAGL1 |
| hsa-miR-152 | TMEM123 |
| hsa-miR-152 | TMEM25 |
| hsa-miR-152 | TMEM57 |
| hsa-miR-152 | TMPRSS4 |
| hsa-miR-152 | TNFRSF1B |
| hsa-miR-152 | TPBG |
| hsa-miR-152 | TRAPPC4 |
| hsa-miR-152 | TRIOBP |
| hsa-miR-152 | TTF2 |
| hsa-miR-152 | TTLL3 |
| hsa-miR-152 | TUBE1 |
| hsa-miR-152 | UQCRQ |
| hsa-miR-152 | USP30 |
| hsa-miR-152 | VNN1 |
| hsa-miR-152 | WDR47 |
| hsa-miR-152 | WDR7 |
| hsa-miR-152 | ZDHHC7 |
| hsa-miR-152 | ZNF488 |
| hsa-miR-152 | ZNF496 |
| hsa-miR-152 | ZNF605 |
| hsa-miR-155 | ABCA1 |
| hsa-miR-155 | ACRV1 |
| hsa-miR-155 | AGTRAP |
| hsa-miR-155 | ANXA2 |
| hsa-miR-155 | AP3M2 |
| hsa-miR-155 | ARRDC2 |
| hsa-miR-155 | BACE1 |
| hsa-miR-155 | BIRC3 |
| hsa-miR-155 | BZW2 |
| hsa-miR-155 | C7orf31 |
| hsa-miR-155 | CCNJL |
| hsa-miR-155 | CD58 |
| hsa-miR-155 | CLCN5 |
| hsa-miR-155 | CMTM1 |
| hsa-miR-155 | DACH2 |
| hsa-miR-155 | DMRT2 |
| hsa-miR-155 | DNAJC10 |
| hsa-miR-155 | DNAJC18 |
| hsa-miR-155 | DRP2 |
| hsa-miR-155 | DUSP14 |
| hsa-miR-155 | EPYC |
| hsa-miR-155 | ERBB3 |
| hsa-miR-155 | FAM126A |
| hsa-miR-155 | FMNL2 |
| hsa-miR-155 | FNDC3A |
| hsa-miR-155 | GOLPH3L |
| hsa-miR-155 | GRIA3 |
| hsa-miR-155 | HDAC6 |
| hsa-miR-155 | IL13 |
| hsa-miR-155 | MARK1 |
| hsa-miR-155 | MPDZ |
| hsa-miR-155 | NEK11 |
| hsa-miR-155 | NT5E |
| hsa-miR-155 | ORAOV1 |
| hsa-miR-155 | PLEKHH2 |
| hsa-miR-155 | PTPRR |
| hsa-miR-155 | RAPH1 |
| hsa-miR-155 | STARD8 |
| hsa-miR-155 | TCERG1 |
| hsa-miR-155 | TRIM36 |
| hsa-miR-155 | VAV3 |
| hsa-miR-155 | VDR |
| hsa-miR-155 | WDR73 |
| hsa-miR-155 | WWC1 |
| hsa-miR-155 | ZNF300 |
| hsa-miR-155 | ZNF528 |
| hsa-miR-155 | ZNF608 |
| hsa-miR-181c | AASS |
| hsa-miR-181c | ACAD8 |
| hsa-miR-181c | ACSL1 |
| hsa-miR-181c | ACTN2 |
| hsa-miR-181c | ADAM15 |
| hsa-miR-181c | ALDH3A2 |
| hsa-miR-181c | ALDH9A1 |
| hsa-miR-181c | ANKRD10 |
| hsa-miR-181c | ANXA4 |
| hsa-miR-181c | ARHGEF3 |
| hsa-miR-181c | ATP11A |
| hsa-miR-181c | ATP11C |
| hsa-miR-181c | B3GALT5 |
| hsa-miR-181c | BACE2 |
| hsa-miR-181c | BTBD3 |
| hsa-miR-181c | BZW2 |
| hsa-miR-181c | CDADC1 |
| hsa-miR-181c | CDCA4 |
| hsa-miR-181c | CEP72 |
| hsa-miR-181c | CFL2 |
| hsa-miR-181c | CKAP2 |
| hsa-miR-181c | COL6A3 |
| hsa-miR-181c | CPOX |
| hsa-miR-181c | CSNK1G1 |
| hsa-miR-181c | CUBN |
| hsa-miR-181c | CYP26A1 |
| hsa-miR-181c | DGKH |
| hsa-miR-181c | DNAH5 |
| hsa-miR-181c | DTNA |
| hsa-miR-181c | E2F7 |
| hsa-miR-181c | ECHDC2 |
| hsa-miR-181c | ENPP1 |
| hsa-miR-181c | F11 |
| hsa-miR-181c | FDX1 |
| hsa-miR-181c | FNDC3A |
| hsa-miR-181c | FOS |
| hsa-miR-181c | GALNT3 |
| hsa-miR-181c | GCLC |
| hsa-miR-181c | GCNT2 |
| hsa-miR-181c | GLYATL1 |
| hsa-miR-181c | GPM6A |
| hsa-miR-181c | GRIA4 |
| hsa-miR-181c | GRID1 |
| hsa-miR-181c | GRM5 |
| hsa-miR-181c | HIP1R |
| hsa-miR-181c | HR |
| hsa-miR-181c | ITGA3 |
| hsa-miR-181c | ITGB1BP1 |
| hsa-miR-181c | KALRN |
| hsa-miR-181c | LAMA3 |
| hsa-miR-181c | LDHC |
| hsa-miR-181c | LDLR |
| hsa-miR-181c | LIN7C |
| hsa-miR-181c | MAPT |
| hsa-miR-181c | MARK1 |
| hsa-miR-181c | MDH1B |
| hsa-miR-181c | MTMR10 |
| hsa-miR-181c | NBEA |
| hsa-miR-181c | NFATC4 |
| hsa-miR-181c | NOTUM |
| hsa-miR-181c | NSMAF |
| hsa-miR-181c | PANK3 |
| hsa-miR-181c | PCSK1 |
| hsa-miR-181c | PCSK6 |
| hsa-miR-181c | PER3 |
| hsa-miR-181c | PNRC1 |
| hsa-miR-181c | PPARA |
| hsa-miR-181c | PPARG |
| hsa-miR-181c | PRKAG2 |
| hsa-miR-181c | PROM2 |
| hsa-miR-181c | RAB37 |
| hsa-miR-181c | RAB38 |
| hsa-miR-181c | RASSF6 |
| hsa-miR-181c | SEL1L |
| hsa-miR-181c | SEMA3B |
| hsa-miR-181c | SERPINE1 |
| hsa-miR-181c | SIGLEC1 |
| hsa-miR-181c | SIPA1L2 |
| hsa-miR-181c | SLC19A2 |
| hsa-miR-181c | SLC20A1 |
| hsa-miR-181c | SLC43A1 |
| hsa-miR-181c | SLC6A6 |
| hsa-miR-181c | SLIT2 |
| hsa-miR-181c | SNX4 |
| hsa-miR-181c | SORCS1 |
| hsa-miR-181c | SPATA20 |
| hsa-miR-181c | SPATS2 |
| hsa-miR-181c | SPP1 |
| hsa-miR-181c | ST6GAL1 |
| hsa-miR-181c | STAT1 |
| hsa-miR-181c | STK32C |
| hsa-miR-181c | STXBP4 |
| hsa-miR-181c | TBC1D22B |
| hsa-miR-181c | TCERG1 |
| hsa-miR-181c | TGFBRAP1 |
| hsa-miR-181c | TIAM1 |
| hsa-miR-181c | TMCO3 |
| hsa-miR-181c | TMEM135 |
| hsa-miR-181c | TMEM26 |
| hsa-miR-181c | TMEM30A |
| hsa-miR-181c | TRPC6 |
| hsa-miR-181c | TSC22D2 |
| hsa-miR-181c | VCAN |
| hsa-miR-181c | ZNF124 |
| hsa-miR-181c | ZNF382 |
| hsa-miR-181c | ZNF514 |
| hsa-miR-181c | ZNF563 |
| hsa-miR-181c | ZSCAN23 |
| hsa-miR-181d | AASS |
| hsa-miR-181d | ACACB |
| hsa-miR-181d | ACLY |
| hsa-miR-181d | ACTN2 |
| hsa-miR-181d | ADAM12 |
| hsa-miR-181d | ALDH9A1 |
| hsa-miR-181d | ANTXR1 |
| hsa-miR-181d | ATP11C |
| hsa-miR-181d | ATP1B2 |
| hsa-miR-181d | B3GALT5 |
| hsa-miR-181d | BCHE |
| hsa-miR-181d | BEX2 |
| hsa-miR-181d | CCDC42 |
| hsa-miR-181d | CCNE1 |
| hsa-miR-181d | CD4 |
| hsa-miR-181d | CDADC1 |
| hsa-miR-181d | CDH23 |
| hsa-miR-181d | CDKN3 |
| hsa-miR-181d | CHL1 |
| hsa-miR-181d | CISH |
| hsa-miR-181d | CIT |
| hsa-miR-181d | CKAP2 |
| hsa-miR-181d | COL6A3 |
| hsa-miR-181d | CPOX |
| hsa-miR-181d | CSNK1G1 |
| hsa-miR-181d | CTTNBP2NL |
| hsa-miR-181d | CUBN |
| hsa-miR-181d | DCLK1 |
| hsa-miR-181d | DCLK2 |
| hsa-miR-181d | DDO |
| hsa-miR-181d | DIRAS3 |
| hsa-miR-181d | DLG5 |
| hsa-miR-181d | DNAJA4 |
| hsa-miR-181d | DYRK2 |
| hsa-miR-181d | E2F7 |
| hsa-miR-181d | ECHDC2 |
| hsa-miR-181d | ENTPD6 |
| hsa-miR-181d | FAIM |
| hsa-miR-181d | FAM96A |
| hsa-miR-181d | FEZ1 |
| hsa-miR-181d | GALNT3 |
| hsa-miR-181d | GATM |
| hsa-miR-181d | GLYATL1 |
| hsa-miR-181d | GOT2 |
| hsa-miR-181d | GPD2 |
| hsa-miR-181d | GREM1 |
| hsa-miR-181d | GRM5 |
| hsa-miR-181d | HIP1R |
| hsa-miR-181d | HPS5 |
| hsa-miR-181d | HR |
| hsa-miR-181d | ITGB1BP1 |
| hsa-miR-181d | ITGB8 |
| hsa-miR-181d | KCNE2 |
| hsa-miR-181d | KCNQ5 |
| hsa-miR-181d | KLK13 |
| hsa-miR-181d | KLRF1 |
| hsa-miR-181d | LAMA3 |
| hsa-miR-181d | LAPTM4B |
| hsa-miR-181d | LDHC |
| hsa-miR-181d | LIN7C |
| hsa-miR-181d | LRIG1 |
| hsa-miR-181d | MAP1A |
| hsa-miR-181d | MCM4 |
| hsa-miR-181d | MCTP2 |
| hsa-miR-181d | MDM4 |
| hsa-miR-181d | MERTK |
| hsa-miR-181d | MX2 |
| hsa-miR-181d | NFATC4 |
| hsa-miR-181d | NLE1 |
| hsa-miR-181d | NOTUM |
| hsa-miR-181d | NR1D2 |
| hsa-miR-181d | NRCAM |
| hsa-miR-181d | NUDT12 |
| hsa-miR-181d | OSBPL6 |
| hsa-miR-181d | PAM |
| hsa-miR-181d | PDGFC |
| hsa-miR-181d | PER3 |
| hsa-miR-181d | PEX13 |
| hsa-miR-181d | PFKFB2 |
| hsa-miR-181d | PLA2G4C |
| hsa-miR-181d | PPARG |
| hsa-miR-181d | PQLC1 |
| hsa-miR-181d | PRDM4 |
| hsa-miR-181d | PRDX3 |
| hsa-miR-181d | PRKAG2 |
| hsa-miR-181d | PROCR |
| hsa-miR-181d | PTPRR |
| hsa-miR-181d | RAB37 |
| hsa-miR-181d | RAD54B |
| hsa-miR-181d | RASSF6 |
| hsa-miR-181d | RSPO2 |
| hsa-miR-181d | RUFY2 |
| hsa-miR-181d | SACM1L |
| hsa-miR-181d | SACS |
| hsa-miR-181d | SAMD4A |
| hsa-miR-181d | SEC24D |
| hsa-miR-181d | SEMA3C |
| hsa-miR-181d | 3-Sep |
| hsa-miR-181d | SERPINE1 |
| hsa-miR-181d | SFXN2 |
| hsa-miR-181d | SIPA1L2 |
| hsa-miR-181d | SLC19A2 |
| hsa-miR-181d | SLC20A1 |
| hsa-miR-181d | SLC22A2 |
| hsa-miR-181d | SLC25A12 |
| hsa-miR-181d | SLC25A25 |
| hsa-miR-181d | SLC25A4 |
| hsa-miR-181d | SLIT2 |
| hsa-miR-181d | SMARCD1 |
| hsa-miR-181d | SNAP25 |
| hsa-miR-181d | SPATA18 |
| hsa-miR-181d | STXBP4 |
| hsa-miR-181d | SYT7 |
| hsa-miR-181d | TFRC |
| hsa-miR-181d | TGDS |
| hsa-miR-181d | TGFBRAP1 |
| hsa-miR-181d | TIAM1 |
| hsa-miR-181d | TMCO3 |
| hsa-miR-181d | TMEM144 |
| hsa-miR-181d | TMEM26 |
| hsa-miR-181d | TMEM30A |
| hsa-miR-181d | VASH2 |
| hsa-miR-181d | YWHAZ |
| hsa-miR-181d | ZDHHC7 |
| hsa-miR-181d | ZFP3 |
| hsa-miR-181d | ZNF124 |
| hsa-miR-181d | ZNF283 |
| hsa-miR-181d | ZNF287 |
| hsa-miR-181d | ZNF365 |
| hsa-miR-181d | ZNF382 |
| hsa-miR-181d | ZNF514 |
| hsa-miR-181d | ZNF528 |
| hsa-miR-181d | ZNF547 |
| hsa-miR-181d | ZNF563 |
| hsa-miR-181d | ZNF605 |
| hsa-miR-181d | ZNF793 |
| hsa-miR-181d | ZNF83 |
| hsa-miR-181d | ZSCAN23 |
| hsa-miR-182 | ABCA1 |
| hsa-miR-182 | ACACB |
| hsa-miR-182 | ACAD10 |
| hsa-miR-182 | ACSS2 |
| hsa-miR-182 | ADAMTS13 |
| hsa-miR-182 | ADCY6 |
| hsa-miR-182 | ADSS |
| hsa-miR-182 | AK5 |
| hsa-miR-182 | ALB |
| hsa-miR-182 | ALDOA |
| hsa-miR-182 | ANG |
| hsa-miR-182 | ANXA11 |
| hsa-miR-182 | ARHGEF3 |
| hsa-miR-182 | ATP11C |
| hsa-miR-182 | ATP1B3 |
| hsa-miR-182 | ATP8B3 |
| hsa-miR-182 | AVPI1 |
| hsa-miR-182 | BDKRB2 |
| hsa-miR-182 | BDNF |
| hsa-miR-182 | BFSP1 |
| hsa-miR-182 | CADM1 |
| hsa-miR-182 | CADM2 |
| hsa-miR-182 | CHRNA4 |
| hsa-miR-182 | CLIP4 |
| hsa-miR-182 | CNKSR1 |
| hsa-miR-182 | CRIM1 |
| hsa-miR-182 | CXorf36 |
| hsa-miR-182 | CYP51A1 |
| hsa-miR-182 | DCTN5 |
| hsa-miR-182 | DENND2C |
| hsa-miR-182 | DGKH |
| hsa-miR-182 | DKK2 |
| hsa-miR-182 | DNAJC10 |
| hsa-miR-182 | DTNA |
| hsa-miR-182 | DYRK2 |
| hsa-miR-182 | EGLN3 |
| hsa-miR-182 | ELF3 |
| hsa-miR-182 | ELMO1 |
| hsa-miR-182 | ENTPD5 |
| hsa-miR-182 | EPM2A |
| hsa-miR-182 | FAH |
| hsa-miR-182 | FHL1 |
| hsa-miR-182 | GALNT3 |
| hsa-miR-182 | GNAO1 |
| hsa-miR-182 | GNB1L |
| hsa-miR-182 | GPAM |
| hsa-miR-182 | GPATCH2 |
| hsa-miR-182 | GPR180 |
| hsa-miR-182 | HIF1A |
| hsa-miR-182 | HK1 |
| hsa-miR-182 | HMGCLL1 |
| hsa-miR-182 | HYAL1 |
| hsa-miR-182 | ITIH5 |
| hsa-miR-182 | IVNS1ABP |
| hsa-miR-182 | KCNIP3 |
| hsa-miR-182 | KCNJ10 |
| hsa-miR-182 | KCTD2 |
| hsa-miR-182 | KHDRBS3 |
| hsa-miR-182 | KIAA0907 |
| hsa-miR-182 | LPIN1 |
| hsa-miR-182 | LRP1 |
| hsa-miR-182 | MAP4K3 |
| hsa-miR-182 | MAPK10 |
| hsa-miR-182 | MCCC2 |
| hsa-miR-182 | MCM4 |
| hsa-miR-182 | MDN1 |
| hsa-miR-182 | MEOX1 |
| hsa-miR-182 | MGST1 |
| hsa-miR-182 | MTSS1 |
| hsa-miR-182 | MVP |
| hsa-miR-182 | MYB |
| hsa-miR-182 | NCAPD2 |
| hsa-miR-182 | NCOA4 |
| hsa-miR-182 | NEDD4 |
| hsa-miR-182 | NQO1 |
| hsa-miR-182 | OIT3 |
| hsa-miR-182 | PDGFD |
| hsa-miR-182 | PDPN |
| hsa-miR-182 | PHF21A |
| hsa-miR-182 | PLCH1 |
| hsa-miR-182 | PML |
| hsa-miR-182 | PPARG |
| hsa-miR-182 | PPFIBP2 |
| hsa-miR-182 | PPM1A |
| hsa-miR-182 | PRDM4 |
| hsa-miR-182 | PRDM5 |
| hsa-miR-182 | PTPRB |
| hsa-miR-182 | RAB27A |
| hsa-miR-182 | RBKS |
| hsa-miR-182 | RFWD3 |
| hsa-miR-182 | RGS20 |
| hsa-miR-182 | SCUBE1 |
| hsa-miR-182 | SH3BP5L |
| hsa-miR-182 | SH3TC2 |
| hsa-miR-182 | SIM2 |
| hsa-miR-182 | SLC13A5 |
| hsa-miR-182 | SLC22A15 |
| hsa-miR-182 | SLC23A2 |
| hsa-miR-182 | SLC26A8 |
| hsa-miR-182 | SLC43A2 |
| hsa-miR-182 | SLCO2B1 |
| hsa-miR-182 | SOAT2 |
| hsa-miR-182 | SPATA18 |
| hsa-miR-182 | SPATS2 |
| hsa-miR-182 | ST8SIA1 |
| hsa-miR-182 | SYDE1 |
| hsa-miR-182 | TGDS |
| hsa-miR-182 | TIAM1 |
| hsa-miR-182 | TMEM26 |
| hsa-miR-182 | TPST2 |
| hsa-miR-182 | TRIM36 |
| hsa-miR-182 | TRMT11 |
| hsa-miR-182 | TTC21A |
| hsa-miR-182 | VASH2 |
| hsa-miR-182 | VEPH1 |
| hsa-miR-182 | WDR47 |
| hsa-miR-182 | XPR1 |
| hsa-miR-182 | ZFAND5 |
| hsa-miR-182 | ZFP1 |
| hsa-miR-182 | ZFP36 |
| hsa-miR-182 | ZNF488 |
| hsa-miR-182 | ZNF607 |
| hsa-miR-182 | ZYG11B |
| hsa-miR-183 | ABCC8 |
| hsa-miR-183 | ABCG5 |
| hsa-miR-183 | ACACB |
| hsa-miR-183 | ACAD8 |
| hsa-miR-183 | ACADVL |
| hsa-miR-183 | ACAN |
| hsa-miR-183 | ACSL1 |
| hsa-miR-183 | ADHFE1 |
| hsa-miR-183 | ADSS |
| hsa-miR-183 | AIG1 |
| hsa-miR-183 | ALAD |
| hsa-miR-183 | ALDH5A1 |
| hsa-miR-183 | ANKS6 |
| hsa-miR-183 | AP3M2 |
| hsa-miR-183 | ARHGEF10L |
| hsa-miR-183 | ASTN1 |
| hsa-miR-183 | ATPIF1 |
| hsa-miR-183 | B3GALNT2 |
| hsa-miR-183 | C20orf96 |
| hsa-miR-183 | C8B |
| hsa-miR-183 | CELSR3 |
| hsa-miR-183 | CLCN2 |
| hsa-miR-183 | CRYGS |
| hsa-miR-183 | DBI |
| hsa-miR-183 | DCLK2 |
| hsa-miR-183 | DENND2C |
| hsa-miR-183 | DGKA |
| hsa-miR-183 | DHCR24 |
| hsa-miR-183 | DIAPH3 |
| hsa-miR-183 | EEF1E1 |
| hsa-miR-183 | EI24 |
| hsa-miR-183 | EPHA2 |
| hsa-miR-183 | EXO1 |
| hsa-miR-183 | FAIM |
| hsa-miR-183 | FBLN1 |
| hsa-miR-183 | FNDC3A |
| hsa-miR-183 | GCA |
| hsa-miR-183 | GP2 |
| hsa-miR-183 | GPR180 |
| hsa-miR-183 | GPR3 |
| hsa-miR-183 | IL17RC |
| hsa-miR-183 | IL1RL1 |
| hsa-miR-183 | IQGAP3 |
| hsa-miR-183 | ITGAV |
| hsa-miR-183 | KIF2A |
| hsa-miR-183 | KLHL32 |
| hsa-miR-183 | LGR6 |
| hsa-miR-183 | MBNL2 |
| hsa-miR-183 | MCM5 |
| hsa-miR-183 | MFN2 |
| hsa-miR-183 | MTMR10 |
| hsa-miR-183 | MUC1 |
| hsa-miR-183 | NCAN |
| hsa-miR-183 | NOVA1 |
| hsa-miR-183 | NPC1L1 |
| hsa-miR-183 | NRCAM |
| hsa-miR-183 | NUP93 |
| hsa-miR-183 | OSBPL10 |
| hsa-miR-183 | OXNAD1 |
| hsa-miR-183 | P4HA2 |
| hsa-miR-183 | PAM |
| hsa-miR-183 | PAQR4 |
| hsa-miR-183 | PCGF6 |
| hsa-miR-183 | PHKA2 |
| hsa-miR-183 | PKHD1 |
| hsa-miR-183 | PRDM5 |
| hsa-miR-183 | RDH11 |
| hsa-miR-183 | RGMA |
| hsa-miR-183 | RGS20 |
| hsa-miR-183 | RRP12 |
| hsa-miR-183 | SCP2 |
| hsa-miR-183 | SEL1L |
| hsa-miR-183 | SFXN1 |
| hsa-miR-183 | SIDT2 |
| hsa-miR-183 | SLC16A2 |
| hsa-miR-183 | SLC43A1 |
| hsa-miR-183 | SLCO3A1 |
| hsa-miR-183 | SOCS2 |
| hsa-miR-183 | SPATS2 |
| hsa-miR-183 | STX6 |
| hsa-miR-183 | STXBP4 |
| hsa-miR-183 | SVIL |
| hsa-miR-183 | TINAG |
| hsa-miR-183 | TMEM119 |
| hsa-miR-183 | TMEM25 |
| hsa-miR-183 | TMEM26 |
| hsa-miR-183 | TMEM52 |
| hsa-miR-183 | TNFRSF21 |
| hsa-miR-183 | TTC7B |
| hsa-miR-183 | TUFT1 |
| hsa-miR-183 | UBE2D4 |
| hsa-miR-183 | ZNF273 |
| hsa-miR-183 | ZNF605 |
| hsa-miR-187 | ACOX3 |
| hsa-miR-187 | ANGPTL7 |
| hsa-miR-187 | C1S |
| hsa-miR-187 | CARD14 |
| hsa-miR-187 | CBS |
| hsa-miR-187 | CCDC40 |
| hsa-miR-187 | CRMP1 |
| hsa-miR-187 | DAB2 |
| hsa-miR-187 | DKK3 |
| hsa-miR-187 | DLG5 |
| hsa-miR-187 | DPP6 |
| hsa-miR-187 | EML2 |
| hsa-miR-187 | FAM19A3 |
| hsa-miR-187 | FHAD1 |
| hsa-miR-187 | GPRC5B |
| hsa-miR-187 | GTSE1 |
| hsa-miR-187 | HFE2 |
| hsa-miR-187 | IQCC |
| hsa-miR-187 | KCTD1 |
| hsa-miR-187 | LRP1 |
| hsa-miR-187 | LRRC36 |
| hsa-miR-187 | MKI67 |
| hsa-miR-187 | MMAA |
| hsa-miR-187 | MYD88 |
| hsa-miR-187 | NR4A2 |
| hsa-miR-187 | NT5M |
| hsa-miR-187 | OCEL1 |
| hsa-miR-187 | PEX7 |
| hsa-miR-187 | PLA2G12A |
| hsa-miR-187 | PLEKHH2 |
| hsa-miR-187 | S100A4 |
| hsa-miR-187 | SCNM1 |
| hsa-miR-187 | SORCS1 |
| hsa-miR-187 | SPAG6 |
| hsa-miR-187 | TBX3 |
| hsa-miR-187 | THSD4 |
| hsa-miR-187 | TREM2 |
| hsa-miR-187 | TRIOBP |
| hsa-miR-187 | UCHL1 |
| hsa-miR-187 | VGLL1 |
| hsa-miR-192 | ABCB5 |
| hsa-miR-192 | ANKRD46 |
| hsa-miR-192 | ATF3 |
| hsa-miR-192 | B3GALNT1 |
| hsa-miR-192 | CARD14 |
| hsa-miR-192 | CAV1 |
| hsa-miR-192 | CCM2 |
| hsa-miR-192 | CDC7 |
| hsa-miR-192 | COL3A1 |
| hsa-miR-192 | CTTNBP2NL |
| hsa-miR-192 | DHTKD1 |
| hsa-miR-192 | DLG3 |
| hsa-miR-192 | DNAH9 |
| hsa-miR-192 | DNAJC10 |
| hsa-miR-192 | FGFR1 |
| hsa-miR-192 | FURIN |
| hsa-miR-192 | GAD1 |
| hsa-miR-192 | GINS1 |
| hsa-miR-192 | GINS4 |
| hsa-miR-192 | HBS1L |
| hsa-miR-192 | HIBADH |
| hsa-miR-192 | KCNQ5 |
| hsa-miR-192 | KDR |
| hsa-miR-192 | KLHL32 |
| hsa-miR-192 | LOXL2 |
| hsa-miR-192 | MCM4 |
| hsa-miR-192 | NID1 |
| hsa-miR-192 | NRIP3 |
| hsa-miR-192 | NSD1 |
| hsa-miR-192 | PAPPA2 |
| hsa-miR-192 | PPM1A |
| hsa-miR-192 | RFWD3 |
| hsa-miR-192 | SAMD4A |
| hsa-miR-192 | SCARA5 |
| hsa-miR-192 | SLC22A15 |
| hsa-miR-192 | SLC39A6 |
| hsa-miR-192 | TMTC1 |
| hsa-miR-192 | TRPM1 |
| hsa-miR-192 | VSX1 |
| hsa-miR-192 | ZNF169 |
| hsa-miR-192 | ZSCAN20 |
| hsa-miR-196b | ABCB9 |
| hsa-miR-196b | ABHD10 |
| hsa-miR-196b | ACAT1 |
| hsa-miR-196b | ADHFE1 |
| hsa-miR-196b | AGR2 |
| hsa-miR-196b | ARHGAP20 |
| hsa-miR-196b | ATF7IP2 |
| hsa-miR-196b | ATP11C |
| hsa-miR-196b | BACE2 |
| hsa-miR-196b | C12orf49 |
| hsa-miR-196b | CACNB2 |
| hsa-miR-196b | CEP250 |
| hsa-miR-196b | COL24A1 |
| hsa-miR-196b | COL3A1 |
| hsa-miR-196b | CS |
| hsa-miR-196b | CYP4B1 |
| hsa-miR-196b | DLG2 |
| hsa-miR-196b | EME1 |
| hsa-miR-196b | EPHA7 |
| hsa-miR-196b | FNDC3A |
| hsa-miR-196b | FOXN1 |
| hsa-miR-196b | FOXRED1 |
| hsa-miR-196b | GALNT10 |
| hsa-miR-196b | GATM |
| hsa-miR-196b | GCLC |
| hsa-miR-196b | GDPD4 |
| hsa-miR-196b | GIPR |
| hsa-miR-196b | GLG1 |
| hsa-miR-196b | GNB3 |
| hsa-miR-196b | GPD1L |
| hsa-miR-196b | GPHN |
| hsa-miR-196b | GULP1 |
| hsa-miR-196b | HADH |
| hsa-miR-196b | ISG20 |
| hsa-miR-196b | ITGAV |
| hsa-miR-196b | KCNQ5 |
| hsa-miR-196b | LAMA4 |
| hsa-miR-196b | MBNL2 |
| hsa-miR-196b | MIF4GD |
| hsa-miR-196b | MRPL34 |
| hsa-miR-196b | NADK |
| hsa-miR-196b | NCDN |
| hsa-miR-196b | NDUFB6 |
| hsa-miR-196b | NEO1 |
| hsa-miR-196b | NOX4 |
| hsa-miR-196b | NPL |
| hsa-miR-196b | OLFML1 |
| hsa-miR-196b | PCTP |
| hsa-miR-196b | PDE2A |
| hsa-miR-196b | PFKFB3 |
| hsa-miR-196b | PIK3C2B |
| hsa-miR-196b | PLCH1 |
| hsa-miR-196b | POMT2 |
| hsa-miR-196b | PRDM5 |
| hsa-miR-196b | PRMT1 |
| hsa-miR-196b | PTPRB |
| hsa-miR-196b | RALGPS1 |
| hsa-miR-196b | RCC2 |
| hsa-miR-196b | RET |
| hsa-miR-196b | RFWD3 |
| hsa-miR-196b | SERPINE1 |
| hsa-miR-196b | SH3TC2 |
| hsa-miR-196b | SIGLEC1 |
| hsa-miR-196b | SLC12A2 |
| hsa-miR-196b | SLC20A1 |
| hsa-miR-196b | SLC25A20 |
| hsa-miR-196b | SLC26A8 |
| hsa-miR-196b | SLC38A4 |
| hsa-miR-196b | SLCO1A2 |
| hsa-miR-196b | SNRK |
| hsa-miR-196b | SOCS2 |
| hsa-miR-196b | SYP |
| hsa-miR-196b | SYT9 |
| hsa-miR-196b | TMEM143 |
| hsa-miR-196b | TMEM144 |
| hsa-miR-196b | TRPM8 |
| hsa-miR-196b | TTC21A |
| hsa-miR-196b | TTF2 |
| hsa-miR-196b | TULP3 |
| hsa-miR-196b | TYRP1 |
| hsa-miR-196b | WNT2B |
| hsa-miR-196b | XRCC4 |
| hsa-miR-196b | ZNF605 |
| hsa-miR-200a | ABCC5 |
| hsa-miR-200a | ABCG2 |
| hsa-miR-200a | ACAD8 |
| hsa-miR-200a | ADHFE1 |
| hsa-miR-200a | ADORA1 |
| hsa-miR-200a | ALDH1L2 |
| hsa-miR-200a | ARHGEF10 |
| hsa-miR-200a | ASB13 |
| hsa-miR-200a | ATP11C |
| hsa-miR-200a | BCL9 |
| hsa-miR-200a | BEST3 |
| hsa-miR-200a | BIRC5 |
| hsa-miR-200a | BRSK2 |
| hsa-miR-200a | C1RL |
| hsa-miR-200a | CD80 |
| hsa-miR-200a | CDADC1 |
| hsa-miR-200a | CHN2 |
| hsa-miR-200a | CLPB |
| hsa-miR-200a | COL24A1 |
| hsa-miR-200a | CTBP2 |
| hsa-miR-200a | DOLPP1 |
| hsa-miR-200a | DTL |
| hsa-miR-200a | DYRK2 |
| hsa-miR-200a | E2F3 |
| hsa-miR-200a | EI24 |
| hsa-miR-200a | EME1 |
| hsa-miR-200a | FBLN5 |
| hsa-miR-200a | FGB |
| hsa-miR-200a | FMO3 |
| hsa-miR-200a | GLYAT |
| hsa-miR-200a | GPAM |
| hsa-miR-200a | GRHL1 |
| hsa-miR-200a | HECTD1 |
| hsa-miR-200a | HPS5 |
| hsa-miR-200a | IGSF11 |
| hsa-miR-200a | ITGA11 |
| hsa-miR-200a | ITGA6 |
| hsa-miR-200a | JAG1 |
| hsa-miR-200a | KDR |
| hsa-miR-200a | KIAA1841 |
| hsa-miR-200a | KLHDC2 |
| hsa-miR-200a | LGR4 |
| hsa-miR-200a | LRP12 |
| hsa-miR-200a | LYPD6 |
| hsa-miR-200a | MAK |
| hsa-miR-200a | MANEA |
| hsa-miR-200a | MPZL1 |
| hsa-miR-200a | MTMR10 |
| hsa-miR-200a | NDP |
| hsa-miR-200a | NOVA1 |
| hsa-miR-200a | OR51E1 |
| hsa-miR-200a | PAOX |
| hsa-miR-200a | PAQR5 |
| hsa-miR-200a | PCK1 |
| hsa-miR-200a | PDGFD |
| hsa-miR-200a | PECR |
| hsa-miR-200a | PFKM |
| hsa-miR-200a | PKP2 |
| hsa-miR-200a | PLCB1 |
| hsa-miR-200a | PMAIP1 |
| hsa-miR-200a | PRDM4 |
| hsa-miR-200a | PRKCE |
| hsa-miR-200a | RAP2C |
| hsa-miR-200a | RAPGEF2 |
| hsa-miR-200a | RER1 |
| hsa-miR-200a | RIMS2 |
| hsa-miR-200a | SACM1L |
| hsa-miR-200a | 2-Sep |
| hsa-miR-200a | SERPINE2 |
| hsa-miR-200a | SH3TC2 |
| hsa-miR-200a | SIGLEC1 |
| hsa-miR-200a | SIPA1L2 |
| hsa-miR-200a | SLC16A3 |
| hsa-miR-200a | SLC19A2 |
| hsa-miR-200a | SLC22A15 |
| hsa-miR-200a | SLC25A16 |
| hsa-miR-200a | SLC30A10 |
| hsa-miR-200a | SNRK |
| hsa-miR-200a | SPATA20 |
| hsa-miR-200a | ST3GAL3 |
| hsa-miR-200a | ST3GAL6 |
| hsa-miR-200a | STK32B |
| hsa-miR-200a | STMN1 |
| hsa-miR-200a | STX6 |
| hsa-miR-200a | SVIL |
| hsa-miR-200a | SYT6 |
| hsa-miR-200a | SYT9 |
| hsa-miR-200a | SYTL5 |
| hsa-miR-200a | TCERG1 |
| hsa-miR-200a | TDRD6 |
| hsa-miR-200a | TF |
| hsa-miR-200a | TFRC |
| hsa-miR-200a | THSD4 |
| hsa-miR-200a | TM4SF1 |
| hsa-miR-200a | TMEM100 |
| hsa-miR-200a | TMEM70 |
| hsa-miR-200a | TMPRSS13 |
| hsa-miR-200a | TPK1 |
| hsa-miR-200a | TTR |
| hsa-miR-200a | UBE2Z |
| hsa-miR-200a | UGP2 |
| hsa-miR-200a | UGT8 |
| hsa-miR-200a | VASH2 |
| hsa-miR-200a | VLDLR |
| hsa-miR-200a | VSX1 |
| hsa-miR-200a | ZFP1 |
| hsa-miR-200a | ZNF740 |
| hsa-miR-200a | ZSCAN23 |
| hsa-miR-200b | ABCD3 |
| hsa-miR-200b | ADAM10 |
| hsa-miR-200b | ANKRD37 |
| hsa-miR-200b | ARHGAP20 |
| hsa-miR-200b | ARHGEF10 |
| hsa-miR-200b | ARHGEF3 |
| hsa-miR-200b | C21orf91 |
| hsa-miR-200b | CCNJL |
| hsa-miR-200b | DTNA |
| hsa-miR-200b | FHL1 |
| hsa-miR-200b | FZD6 |
| hsa-miR-200b | GDA |
| hsa-miR-200b | GPM6A |
| hsa-miR-200b | GPR107 |
| hsa-miR-200b | GRIA3 |
| hsa-miR-200b | HMGCLL1 |
| hsa-miR-200b | IGSF11 |
| hsa-miR-200b | JAG1 |
| hsa-miR-200b | KCNK2 |
| hsa-miR-200b | LETM2 |
| hsa-miR-200b | LPIN1 |
| hsa-miR-200b | LRRC31 |
| hsa-miR-200b | MAP4K3 |
| hsa-miR-200b | MAPRE1 |
| hsa-miR-200b | MMAA |
| hsa-miR-200b | MMD |
| hsa-miR-200b | MYLK |
| hsa-miR-200b | NOVA1 |
| hsa-miR-200b | NRG1 |
| hsa-miR-200b | NSMAF |
| hsa-miR-200b | NUDT12 |
| hsa-miR-200b | NXF3 |
| hsa-miR-200b | OBSCN |
| hsa-miR-200b | PPP2R1B |
| hsa-miR-200b | RAPGEF2 |
| hsa-miR-200b | RASSF6 |
| hsa-miR-200b | RASSF8 |
| hsa-miR-200b | RIMS2 |
| hsa-miR-200b | SCML2 |
| hsa-miR-200b | SEC14L2 |
| hsa-miR-200b | SLAIN1 |
| hsa-miR-200b | SLC13A3 |
| hsa-miR-200b | SLC30A10 |
| hsa-miR-200b | SLC35F2 |
| hsa-miR-200b | SLC39A4 |
| hsa-miR-200b | SMARCD1 |
| hsa-miR-200b | SSFA2 |
| hsa-miR-200b | SYT10 |
| hsa-miR-200b | TJP2 |
| hsa-miR-200b | TMEM165 |
| hsa-miR-200b | TMEM57 |
| hsa-miR-200b | TUFT1 |
| hsa-miR-200b | TULP3 |
| hsa-miR-200b | USP18 |
| hsa-miR-200b | ZNF365 |
| hsa-miR-200b | ZNF692 |
| hsa-miR-200c | ABCB5 |
| hsa-miR-200c | ABCD3 |
| hsa-miR-200c | ANKRD37 |
| hsa-miR-200c | ARHGEF1 |
| hsa-miR-200c | ARHGEF3 |
| hsa-miR-200c | BBS12 |
| hsa-miR-200c | BCHE |
| hsa-miR-200c | C21orf91 |
| hsa-miR-200c | CD80 |
| hsa-miR-200c | CFL2 |
| hsa-miR-200c | CIT |
| hsa-miR-200c | CPEB4 |
| hsa-miR-200c | CTNND2 |
| hsa-miR-200c | DLC1 |
| hsa-miR-200c | DMRT2 |
| hsa-miR-200c | DNAJC10 |
| hsa-miR-200c | ERC2 |
| hsa-miR-200c | ERRFI1 |
| hsa-miR-200c | FHL1 |
| hsa-miR-200c | FHOD1 |
| hsa-miR-200c | FZD6 |
| hsa-miR-200c | GDA |
| hsa-miR-200c | GPM6A |
| hsa-miR-200c | GPR107 |
| hsa-miR-200c | GPR180 |
| hsa-miR-200c | GRIA3 |
| hsa-miR-200c | GULP1 |
| hsa-miR-200c | HAL |
| hsa-miR-200c | HCFC2 |
| hsa-miR-200c | HMGCLL1 |
| hsa-miR-200c | HPS5 |
| hsa-miR-200c | HS3ST1 |
| hsa-miR-200c | JAG1 |
| hsa-miR-200c | KCNK2 |
| hsa-miR-200c | KDR |
| hsa-miR-200c | KRT80 |
| hsa-miR-200c | LPIN1 |
| hsa-miR-200c | MAP4K3 |
| hsa-miR-200c | MAPK13 |
| hsa-miR-200c | MARK1 |
| hsa-miR-200c | MMD |
| hsa-miR-200c | NEDD4 |
| hsa-miR-200c | NOVA1 |
| hsa-miR-200c | NRCAM |
| hsa-miR-200c | NXF3 |
| hsa-miR-200c | PAM |
| hsa-miR-200c | PCSK6 |
| hsa-miR-200c | PIGV |
| hsa-miR-200c | PKP1 |
| hsa-miR-200c | PPP2R1B |
| hsa-miR-200c | PRKAG2 |
| hsa-miR-200c | RAPGEF2 |
| hsa-miR-200c | RASGEF1B |
| hsa-miR-200c | RASSF6 |
| hsa-miR-200c | RUFY2 |
| hsa-miR-200c | SBF1 |
| hsa-miR-200c | SETD3 |
| hsa-miR-200c | SLAIN1 |
| hsa-miR-200c | SLC13A3 |
| hsa-miR-200c | SLC25A36 |
| hsa-miR-200c | SLC30A10 |
| hsa-miR-200c | SLC35F2 |
| hsa-miR-200c | SLC4A10 |
| hsa-miR-200c | SMARCD1 |
| hsa-miR-200c | SNURF |
| hsa-miR-200c | SSFA2 |
| hsa-miR-200c | ST7 |
| hsa-miR-200c | SYT10 |
| hsa-miR-200c | TACC1 |
| hsa-miR-200c | TBX4 |
| hsa-miR-200c | TJP2 |
| hsa-miR-200c | TMEM25 |
| hsa-miR-200c | TMEM26 |
| hsa-miR-200c | TMTC1 |
| hsa-miR-200c | TPMT |
| hsa-miR-200c | TUFT1 |
| hsa-miR-200c | UGT8 |
| hsa-miR-200c | UXS1 |
| hsa-miR-200c | ZNF365 |
| hsa-miR-200c | ZNF692 |
| hsa-miR-200c | ZSCAN20 |
| hsa-miR-205 | ABCA1 |
| hsa-miR-205 | ABCC5 |
| hsa-miR-205 | ABI2 |
| hsa-miR-205 | ACSL1 |
| hsa-miR-205 | ADH6 |
| hsa-miR-205 | AIG1 |
| hsa-miR-205 | ALB |
| hsa-miR-205 | ANKS6 |
| hsa-miR-205 | ANTXR1 |
| hsa-miR-205 | ANXA4 |
| hsa-miR-205 | AP3M2 |
| hsa-miR-205 | ARL4D |
| hsa-miR-205 | ATP10A |
| hsa-miR-205 | ATP8B4 |
| hsa-miR-205 | ATPIF1 |
| hsa-miR-205 | B3GALT5 |
| hsa-miR-205 | BDNF |
| hsa-miR-205 | BMPER |
| hsa-miR-205 | BTBD3 |
| hsa-miR-205 | C11orf49 |
| hsa-miR-205 | C3orf52 |
| hsa-miR-205 | CAT |
| hsa-miR-205 | CCDC113 |
| hsa-miR-205 | CCDC78 |
| hsa-miR-205 | CCL13 |
| hsa-miR-205 | CD44 |
| hsa-miR-205 | CEP170 |
| hsa-miR-205 | CLEC4M |
| hsa-miR-205 | CLUL1 |
| hsa-miR-205 | CREM |
| hsa-miR-205 | CYP26A1 |
| hsa-miR-205 | CYP4B1 |
| hsa-miR-205 | DACH2 |
| hsa-miR-205 | DHCR24 |
| hsa-miR-205 | DKK2 |
| hsa-miR-205 | DLG2 |
| hsa-miR-205 | DLGAP2 |
| hsa-miR-205 | DNAH9 |
| hsa-miR-205 | DNAJC16 |
| hsa-miR-205 | DOK1 |
| hsa-miR-205 | DPYD |
| hsa-miR-205 | DSN1 |
| hsa-miR-205 | DYRK2 |
| hsa-miR-205 | EBF3 |
| hsa-miR-205 | EFCAB5 |
| hsa-miR-205 | ELF3 |
| hsa-miR-205 | ENPP5 |
| hsa-miR-205 | ERBB3 |
| hsa-miR-205 | ERC2 |
| hsa-miR-205 | ETV7 |
| hsa-miR-205 | F2 |
| hsa-miR-205 | FADS1 |
| hsa-miR-205 | FAM126A |
| hsa-miR-205 | FAM129B |
| hsa-miR-205 | FAS |
| hsa-miR-205 | FEZ1 |
| hsa-miR-205 | FHL1 |
| hsa-miR-205 | FNDC3A |
| hsa-miR-205 | FUBP1 |
| hsa-miR-205 | GABARAPL1 |
| hsa-miR-205 | GINS3 |
| hsa-miR-205 | GIPR |
| hsa-miR-205 | GLYATL1 |
| hsa-miR-205 | GPAM |
| hsa-miR-205 | GPC6 |
| hsa-miR-205 | GPX3 |
| hsa-miR-205 | GRAMD1C |
| hsa-miR-205 | GRHL1 |
| hsa-miR-205 | GRIA4 |
| hsa-miR-205 | GTF3A |
| hsa-miR-205 | HDAC9 |
| hsa-miR-205 | HMGCLL1 |
| hsa-miR-205 | HOOK2 |
| hsa-miR-205 | HSD17B12 |
| hsa-miR-205 | HSD17B6 |
| hsa-miR-205 | INTS6 |
| hsa-miR-205 | JPH1 |
| hsa-miR-205 | KCND3 |
| hsa-miR-205 | KCNJ16 |
| hsa-miR-205 | KNDC1 |
| hsa-miR-205 | LGI2 |
| hsa-miR-205 | LIMK1 |
| hsa-miR-205 | LIMS2 |
| hsa-miR-205 | LIN9 |
| hsa-miR-205 | LIPH |
| hsa-miR-205 | LRP1 |
| hsa-miR-205 | LRRFIP2 |
| hsa-miR-205 | LYPD6 |
| hsa-miR-205 | MAPK3 |
| hsa-miR-205 | MARK1 |
| hsa-miR-205 | MND1 |
| hsa-miR-205 | MOCS2 |
| hsa-miR-205 | MSH2 |
| hsa-miR-205 | MTMR11 |
| hsa-miR-205 | MYRIP |
| hsa-miR-205 | NCAN |
| hsa-miR-205 | NEK6 |
| hsa-miR-205 | NHS |
| hsa-miR-205 | NMD3 |
| hsa-miR-205 | NR3C2 |
| hsa-miR-205 | OSBPL10 |
| hsa-miR-205 | PAM |
| hsa-miR-205 | PAPPA2 |
| hsa-miR-205 | PCK1 |
| hsa-miR-205 | PCSK1 |
| hsa-miR-205 | PCSK6 |
| hsa-miR-205 | PDE11A |
| hsa-miR-205 | PDE1C |
| hsa-miR-205 | PDZD3 |
| hsa-miR-205 | PGM1 |
| hsa-miR-205 | PKD2L1 |
| hsa-miR-205 | PLCB1 |
| hsa-miR-205 | PODXL |
| hsa-miR-205 | PTK7 |
| hsa-miR-205 | RAB11FIP1 |
| hsa-miR-205 | RASGEF1B |
| hsa-miR-205 | RAVER2 |
| hsa-miR-205 | RIT1 |
| hsa-miR-205 | RND1 |
| hsa-miR-205 | SALL2 |
| hsa-miR-205 | SCMH1 |
| hsa-miR-205 | SCNN1A |
| hsa-miR-205 | SCRN2 |
| hsa-miR-205 | SCUBE1 |
| hsa-miR-205 | 2-Sep |
| hsa-miR-205 | 4-Sep |
| hsa-miR-205 | SFXN1 |
| hsa-miR-205 | SH3GL3 |
| hsa-miR-205 | SIGLEC7 |
| hsa-miR-205 | SIM2 |
| hsa-miR-205 | SIPA1L2 |
| hsa-miR-205 | SLC12A2 |
| hsa-miR-205 | SLC13A5 |
| hsa-miR-205 | SLC19A2 |
| hsa-miR-205 | SLC22A9 |
| hsa-miR-205 | SLC7A7 |
| hsa-miR-205 | SLCO2A1 |
| hsa-miR-205 | SLFN5 |
| hsa-miR-205 | SMTN |
| hsa-miR-205 | SP6 |
| hsa-miR-205 | SPATA18 |
| hsa-miR-205 | SSFA2 |
| hsa-miR-205 | ST3GAL6 |
| hsa-miR-205 | STAB2 |
| hsa-miR-205 | STYK1 |
| hsa-miR-205 | TEKT2 |
| hsa-miR-205 | TESK2 |
| hsa-miR-205 | TINAGL1 |
| hsa-miR-205 | TMC2 |
| hsa-miR-205 | TMEM144 |
| hsa-miR-205 | TMEM26 |
| hsa-miR-205 | TNC |
| hsa-miR-205 | TPM4 |
| hsa-miR-205 | TPST2 |
| hsa-miR-205 | TRIO |
| hsa-miR-205 | TRIP10 |
| hsa-miR-205 | TRPM8 |
| hsa-miR-205 | UAP1 |
| hsa-miR-205 | UBE2Z |
| hsa-miR-205 | UGT3A1 |
| hsa-miR-205 | UNC93A |
| hsa-miR-205 | VASH2 |
| hsa-miR-205 | VIM |
| hsa-miR-205 | WDR35 |
| hsa-miR-205 | WWC1 |
| hsa-miR-205 | ZBTB16 |
| hsa-miR-205 | ZIK1 |
| hsa-miR-205 | ZNF300 |
| hsa-miR-205 | ZNF605 |
| hsa-miR-205 | ZYG11B |
| hsa-miR-21 | ABAT |
| hsa-miR-21 | ABCC9 |
| hsa-miR-21 | ABCD3 |
| hsa-miR-21 | ACBD5 |
| hsa-miR-21 | ANXA4 |
| hsa-miR-21 | APOC1 |
| hsa-miR-21 | ATP11A |
| hsa-miR-21 | ATP13A4 |
| hsa-miR-21 | BBS12 |
| hsa-miR-21 | BEST3 |
| hsa-miR-21 | CCR7 |
| hsa-miR-21 | CD160 |
| hsa-miR-21 | CISH |
| hsa-miR-21 | CLIP4 |
| hsa-miR-21 | CNOT6 |
| hsa-miR-21 | CRIM1 |
| hsa-miR-21 | CTSB |
| hsa-miR-21 | CUBN |
| hsa-miR-21 | DCUN1D4 |
| hsa-miR-21 | DLG2 |
| hsa-miR-21 | DNAJC12 |
| hsa-miR-21 | DNMT3B |
| hsa-miR-21 | DUSP10 |
| hsa-miR-21 | EDIL3 |
| hsa-miR-21 | EHBP1 |
| hsa-miR-21 | FUBP1 |
| hsa-miR-21 | GALNT12 |
| hsa-miR-21 | GLUL |
| hsa-miR-21 | GPAM |
| hsa-miR-21 | GPD1L |
| hsa-miR-21 | GPR180 |
| hsa-miR-21 | GULP1 |
| hsa-miR-21 | HBS1L |
| hsa-miR-21 | HDAC9 |
| hsa-miR-21 | ITGA2 |
| hsa-miR-21 | ITGAM |
| hsa-miR-21 | ITIH5 |
| hsa-miR-21 | JAG1 |
| hsa-miR-21 | KCNT2 |
| hsa-miR-21 | LAMA4 |
| hsa-miR-21 | LGR6 |
| hsa-miR-21 | MARK1 |
| hsa-miR-21 | MS4A6A |
| hsa-miR-21 | MSH2 |
| hsa-miR-21 | NCOA4 |
| hsa-miR-21 | NEB |
| hsa-miR-21 | NKIRAS1 |
| hsa-miR-21 | OXNAD1 |
| hsa-miR-21 | PAQR5 |
| hsa-miR-21 | PHLDB1 |
| hsa-miR-21 | PIK3R4 |
| hsa-miR-21 | PTGFR |
| hsa-miR-21 | RIT1 |
| hsa-miR-21 | RNF185 |
| hsa-miR-21 | SASS6 |
| hsa-miR-21 | 2-Sep |
| hsa-miR-21 | SH3GL3 |
| hsa-miR-21 | SLC13A3 |
| hsa-miR-21 | SLC26A2 |
| hsa-miR-21 | SLC4A10 |
| hsa-miR-21 | SMARCA2 |
| hsa-miR-21 | SOX5 |
| hsa-miR-21 | STYK1 |
| hsa-miR-21 | TIAM1 |
| hsa-miR-21 | TMEM55A |
| hsa-miR-21 | TPK1 |
| hsa-miR-21 | UAP1L1 |
| hsa-miR-21 | UGT3A1 |
| hsa-miR-21 | ZHX1 |
| hsa-miR-211 | AACS |
| hsa-miR-211 | ACSS2 |
| hsa-miR-211 | ADAMTS18 |
| hsa-miR-211 | ADCY6 |
| hsa-miR-211 | ADD2 |
| hsa-miR-211 | ADRA1A |
| hsa-miR-211 | AKAP1 |
| hsa-miR-211 | ANKRD10 |
| hsa-miR-211 | ANKS6 |
| hsa-miR-211 | ANXA11 |
| hsa-miR-211 | ANXA4 |
| hsa-miR-211 | ARHGAP10 |
| hsa-miR-211 | ASB13 |
| hsa-miR-211 | ASRGL1 |
| hsa-miR-211 | ATP10B |
| hsa-miR-211 | ATP6V1C2 |
| hsa-miR-211 | BCL9 |
| hsa-miR-211 | C12orf29 |
| hsa-miR-211 | CADM1 |
| hsa-miR-211 | CBFA2T2 |
| hsa-miR-211 | CCDC25 |
| hsa-miR-211 | CCDC40 |
| hsa-miR-211 | CD163 |
| hsa-miR-211 | CD80 |
| hsa-miR-211 | CDC7 |
| hsa-miR-211 | CENPL |
| hsa-miR-211 | CES2 |
| hsa-miR-211 | CHEK1 |
| hsa-miR-211 | CLIP4 |
| hsa-miR-211 | COL16A1 |
| hsa-miR-211 | COL17A1 |
| hsa-miR-211 | COL5A3 |
| hsa-miR-211 | COQ3 |
| hsa-miR-211 | CYP27A1 |
| hsa-miR-211 | CYP2J2 |
| hsa-miR-211 | DENND3 |
| hsa-miR-211 | DENND4B |
| hsa-miR-211 | DLG2 |
| hsa-miR-211 | DLG3 |
| hsa-miR-211 | DMGDH |
| hsa-miR-211 | DTNA |
| hsa-miR-211 | DUSP14 |
| hsa-miR-211 | DUSP4 |
| hsa-miR-211 | EEF1E1 |
| hsa-miR-211 | ELMO1 |
| hsa-miR-211 | EPHA7 |
| hsa-miR-211 | EPHB2 |
| hsa-miR-211 | ERC2 |
| hsa-miR-211 | FAH |
| hsa-miR-211 | FAIM |
| hsa-miR-211 | FAP |
| hsa-miR-211 | FEZ1 |
| hsa-miR-211 | FRMD4A |
| hsa-miR-211 | GBGT1 |
| hsa-miR-211 | GCNT2 |
| hsa-miR-211 | GHITM |
| hsa-miR-211 | GINS3 |
| hsa-miR-211 | GLRB |
| hsa-miR-211 | GNB3 |
| hsa-miR-211 | GP2 |
| hsa-miR-211 | GPD1L |
| hsa-miR-211 | GPR180 |
| hsa-miR-211 | GRAMD1C |
| hsa-miR-211 | GRHL2 |
| hsa-miR-211 | GRIA3 |
| hsa-miR-211 | HBS1L |
| hsa-miR-211 | HCFC2 |
| hsa-miR-211 | HDAC9 |
| hsa-miR-211 | HMGA1 |
| hsa-miR-211 | HMGN4 |
| hsa-miR-211 | HOXD1 |
| hsa-miR-211 | HPCA |
| hsa-miR-211 | IL1RL1 |
| hsa-miR-211 | ITGA11 |
| hsa-miR-211 | KALRN |
| hsa-miR-211 | KCNH8 |
| hsa-miR-211 | KCNK9 |
| hsa-miR-211 | KHDRBS3 |
| hsa-miR-211 | LGR6 |
| hsa-miR-211 | LOXL2 |
| hsa-miR-211 | MCOLN2 |
| hsa-miR-211 | MMAA |
| hsa-miR-211 | MOCS2 |
| hsa-miR-211 | MT1X |
| hsa-miR-211 | MTMR4 |
| hsa-miR-211 | MX1 |
| hsa-miR-211 | MYLK |
| hsa-miR-211 | MYO18B |
| hsa-miR-211 | NOL3 |
| hsa-miR-211 | NPL |
| hsa-miR-211 | NR3C2 |
| hsa-miR-211 | OFD1 |
| hsa-miR-211 | OXNAD1 |
| hsa-miR-211 | PAM |
| hsa-miR-211 | PANK1 |
| hsa-miR-211 | PAQR8 |
| hsa-miR-211 | PDE4A |
| hsa-miR-211 | PDHX |
| hsa-miR-211 | PEX19 |
| hsa-miR-211 | PIF1 |
| hsa-miR-211 | PITX2 |
| hsa-miR-211 | PJA1 |
| hsa-miR-211 | PLA2G12B |
| hsa-miR-211 | PLCH1 |
| hsa-miR-211 | PML |
| hsa-miR-211 | POGK |
| hsa-miR-211 | PPARGC1A |
| hsa-miR-211 | PPFIBP2 |
| hsa-miR-211 | PRAME |
| hsa-miR-211 | PROC |
| hsa-miR-211 | PRPF3 |
| hsa-miR-211 | RAB11FIP1 |
| hsa-miR-211 | RAB27B |
| hsa-miR-211 | RAD23B |
| hsa-miR-211 | RARG |
| hsa-miR-211 | RBP5 |
| hsa-miR-211 | RDH11 |
| hsa-miR-211 | REEP2 |
| hsa-miR-211 | RELB |
| hsa-miR-211 | RET |
| hsa-miR-211 | RETSAT |
| hsa-miR-211 | RGS16 |
| hsa-miR-211 | RIMS2 |
| hsa-miR-211 | RNF125 |
| hsa-miR-211 | RORA |
| hsa-miR-211 | SAMD13 |
| hsa-miR-211 | SASS6 |
| hsa-miR-211 | SAT1 |
| hsa-miR-211 | 4-Sep |
| hsa-miR-211 | SFXN2 |
| hsa-miR-211 | SFXN5 |
| hsa-miR-211 | SHE |
| hsa-miR-211 | SHOX2 |
| hsa-miR-211 | SLC13A4 |
| hsa-miR-211 | SLC19A2 |
| hsa-miR-211 | SLC25A20 |
| hsa-miR-211 | SLC25A4 |
| hsa-miR-211 | SLC26A3 |
| hsa-miR-211 | SLC2A6 |
| hsa-miR-211 | SLC31A2 |
| hsa-miR-211 | SLC39A4 |
| hsa-miR-211 | SLC43A1 |
| hsa-miR-211 | SLC44A2 |
| hsa-miR-211 | SLC6A12 |
| hsa-miR-211 | SMARCA1 |
| hsa-miR-211 | SMARCD1 |
| hsa-miR-211 | SNTG2 |
| hsa-miR-211 | SOCS7 |
| hsa-miR-211 | SPIRE1 |
| hsa-miR-211 | ST3GAL3 |
| hsa-miR-211 | ST7 |
| hsa-miR-211 | STYK1 |
| hsa-miR-211 | SYNE1 |
| hsa-miR-211 | TACC1 |
| hsa-miR-211 | TBX3 |
| hsa-miR-211 | THBS2 |
| hsa-miR-211 | THPO |
| hsa-miR-211 | THSD4 |
| hsa-miR-211 | TKTL1 |
| hsa-miR-211 | TMEM26 |
| hsa-miR-211 | TNFRSF19 |
| hsa-miR-211 | TPM3 |
| hsa-miR-211 | TTYH1 |
| hsa-miR-211 | TXNIP |
| hsa-miR-211 | UBAP2L |
| hsa-miR-211 | VASP |
| hsa-miR-211 | VCAN |
| hsa-miR-211 | WDR7 |
| hsa-miR-211 | WNK3 |
| hsa-miR-211 | XRCC4 |
| hsa-miR-211 | YWHAZ |
| hsa-miR-211 | ZCCHC6 |
| hsa-miR-211 | ZDHHC14 |
| hsa-miR-211 | ZNF286A |
| hsa-miR-211 | ZNF300 |
| hsa-miR-211 | ZNF365 |
| hsa-miR-22 | ABCC11 |
| hsa-miR-22 | ABI2 |
| hsa-miR-22 | ACAT1 |
| hsa-miR-22 | ACSS2 |
| hsa-miR-22 | ADAM12 |
| hsa-miR-22 | ADAMTSL2 |
| hsa-miR-22 | ADCY6 |
| hsa-miR-22 | ADCY7 |
| hsa-miR-22 | ADORA1 |
| hsa-miR-22 | ANKS6 |
| hsa-miR-22 | ANXA13 |
| hsa-miR-22 | ANXA3 |
| hsa-miR-22 | AP3M2 |
| hsa-miR-22 | ARHGAP4 |
| hsa-miR-22 | ARHGAP8 |
| hsa-miR-22 | ARHGEF10L |
| hsa-miR-22 | ARHGEF3 |
| hsa-miR-22 | ARNTL |
| hsa-miR-22 | ATPIF1 |
| hsa-miR-22 | B3GNTL1 |
| hsa-miR-22 | BCAS1 |
| hsa-miR-22 | BTBD3 |
| hsa-miR-22 | C11orf54 |
| hsa-miR-22 | C15orf39 |
| hsa-miR-22 | C17orf53 |
| hsa-miR-22 | CADM1 |
| hsa-miR-22 | CAPN1 |
| hsa-miR-22 | CARD14 |
| hsa-miR-22 | CCDC24 |
| hsa-miR-22 | CCDC40 |
| hsa-miR-22 | CCM2 |
| hsa-miR-22 | CCNJL |
| hsa-miR-22 | CD151 |
| hsa-miR-22 | CD1D |
| hsa-miR-22 | CDH16 |
| hsa-miR-22 | CECR2 |
| hsa-miR-22 | CHRD |
| hsa-miR-22 | CIDEC |
| hsa-miR-22 | CISH |
| hsa-miR-22 | CIT |
| hsa-miR-22 | CLPB |
| hsa-miR-22 | CNKSR1 |
| hsa-miR-22 | CPA6 |
| hsa-miR-22 | CPEB1 |
| hsa-miR-22 | CRHR1 |
| hsa-miR-22 | CRIM1 |
| hsa-miR-22 | CRIP2 |
| hsa-miR-22 | CRLS1 |
| hsa-miR-22 | CSMD2 |
| hsa-miR-22 | CTTNBP2NL |
| hsa-miR-22 | CYP2A7 |
| hsa-miR-22 | DMRT2 |
| hsa-miR-22 | DNAJC16 |
| hsa-miR-22 | DNAL4 |
| hsa-miR-22 | DOK3 |
| hsa-miR-22 | DOK7 |
| hsa-miR-22 | DRP2 |
| hsa-miR-22 | DTNA |
| hsa-miR-22 | DUSP26 |
| hsa-miR-22 | ECHDC2 |
| hsa-miR-22 | ECT2 |
| hsa-miR-22 | EPM2A |
| hsa-miR-22 | ERBB3 |
| hsa-miR-22 | FA2H |
| hsa-miR-22 | FABP1 |
| hsa-miR-22 | FAM117A |
| hsa-miR-22 | FAM49B |
| hsa-miR-22 | FAM96A |
| hsa-miR-22 | FBXL19 |
| hsa-miR-22 | FGFR1 |
| hsa-miR-22 | FIBCD1 |
| hsa-miR-22 | FMNL2 |
| hsa-miR-22 | FMOD |
| hsa-miR-22 | FOS |
| hsa-miR-22 | FTL |
| hsa-miR-22 | FUT8 |
| hsa-miR-22 | FZD6 |
| hsa-miR-22 | GATM |
| hsa-miR-22 | GGCX |
| hsa-miR-22 | GHRHR |
| hsa-miR-22 | GPR107 |
| hsa-miR-22 | GPT2 |
| hsa-miR-22 | GYG2 |
| hsa-miR-22 | HCN3 |
| hsa-miR-22 | HECTD1 |
| hsa-miR-22 | HOMER3 |
| hsa-miR-22 | HOOK2 |
| hsa-miR-22 | HPGD |
| hsa-miR-22 | HTR3A |
| hsa-miR-22 | IL1RL1 |
| hsa-miR-22 | INTS3 |
| hsa-miR-22 | INVS |
| hsa-miR-22 | IQGAP3 |
| hsa-miR-22 | ITGA3 |
| hsa-miR-22 | ITGA5 |
| hsa-miR-22 | ITSN1 |
| hsa-miR-22 | KALRN |
| hsa-miR-22 | KCNG1 |
| hsa-miR-22 | KIF18A |
| hsa-miR-22 | KLHDC8A |
| hsa-miR-22 | KRT80 |
| hsa-miR-22 | LDB1 |
| hsa-miR-22 | LGALS1 |
| hsa-miR-22 | LIMK1 |
| hsa-miR-22 | LOXL2 |
| hsa-miR-22 | LRRC1 |
| hsa-miR-22 | MAP1B |
| hsa-miR-22 | MAPK10 |
| hsa-miR-22 | MAPK12 |
| hsa-miR-22 | MAST2 |
| hsa-miR-22 | MEF2B |
| hsa-miR-22 | MEIS2 |
| hsa-miR-22 | MFGE8 |
| hsa-miR-22 | MINPP1 |
| hsa-miR-22 | MLKL |
| hsa-miR-22 | MOCS1 |
| hsa-miR-22 | MRPS22 |
| hsa-miR-22 | MTMR10 |
| hsa-miR-22 | MTMR11 |
| hsa-miR-22 | MTMR2 |
| hsa-miR-22 | MTMR4 |
| hsa-miR-22 | MYEF2 |
| hsa-miR-22 | NF2 |
| hsa-miR-22 | NPNT |
| hsa-miR-22 | NRSN2 |
| hsa-miR-22 | NSDHL |
| hsa-miR-22 | NT5E |
| hsa-miR-22 | OASL |
| hsa-miR-22 | OIT3 |
| hsa-miR-22 | OLFML1 |
| hsa-miR-22 | OXNAD1 |
| hsa-miR-22 | PANK1 |
| hsa-miR-22 | PCSK9 |
| hsa-miR-22 | PCTP |
| hsa-miR-22 | PDE2A |
| hsa-miR-22 | PHLDA3 |
| hsa-miR-22 | POGK |
| hsa-miR-22 | PPIF |
| hsa-miR-22 | PRDM4 |
| hsa-miR-22 | PRKCE |
| hsa-miR-22 | PTPRN |
| hsa-miR-22 | RAB26 |
| hsa-miR-22 | RCL1 |
| hsa-miR-22 | RET |
| hsa-miR-22 | RFXANK |
| hsa-miR-22 | RGS10 |
| hsa-miR-22 | RHBG |
| hsa-miR-22 | RIC8B |
| hsa-miR-22 | SCP2 |
| hsa-miR-22 | SEC24D |
| hsa-miR-22 | SEMA3B |
| hsa-miR-22 | SEMA4A |
| hsa-miR-22 | SERPINA5 |
| hsa-miR-22 | SETD3 |
| hsa-miR-22 | SFXN3 |
| hsa-miR-22 | SHF |
| hsa-miR-22 | SLC20A2 |
| hsa-miR-22 | SLC22A11 |
| hsa-miR-22 | SLC22A9 |
| hsa-miR-22 | SLC25A22 |
| hsa-miR-22 | SLC25A26 |
| hsa-miR-22 | SLC26A9 |
| hsa-miR-22 | SLC28A1 |
| hsa-miR-22 | SLC4A11 |
| hsa-miR-22 | SLC7A2 |
| hsa-miR-22 | SNRK |
| hsa-miR-22 | SOD2 |
| hsa-miR-22 | SORCS1 |
| hsa-miR-22 | SPATS2 |
| hsa-miR-22 | STX6 |
| hsa-miR-22 | SV2A |
| hsa-miR-22 | SYK |
| hsa-miR-22 | SYNE1 |
| hsa-miR-22 | TACC2 |
| hsa-miR-22 | TACC3 |
| hsa-miR-22 | TAX1BP3 |
| hsa-miR-22 | TESK2 |
| hsa-miR-22 | TIAM1 |
| hsa-miR-22 | TMCC2 |
| hsa-miR-22 | TMEM159 |
| hsa-miR-22 | TMEM44 |
| hsa-miR-22 | TP53 |
| hsa-miR-22 | TRUB2 |
| hsa-miR-22 | TSEN54 |
| hsa-miR-22 | TSPAN15 |
| hsa-miR-22 | TTLL6 |
| hsa-miR-22 | TTLL9 |
| hsa-miR-22 | TUBB6 |
| hsa-miR-22 | TULP3 |
| hsa-miR-22 | UBE2Z |
| hsa-miR-22 | UGT3A1 |
| hsa-miR-22 | VANGL1 |
| hsa-miR-22 | VASP |
| hsa-miR-22 | ZNF169 |
| hsa-miR-22 | ZNF707 |
| hsa-miR-22 | ZNF740 |
| hsa-miR-221 | ABCC9 |
| hsa-miR-221 | ABL2 |
| hsa-miR-221 | ADD2 |
| hsa-miR-221 | AKAP1 |
| hsa-miR-221 | ALDH1A1 |
| hsa-miR-221 | ANKRD10 |
| hsa-miR-221 | ARHGAP10 |
| hsa-miR-221 | ASTN1 |
| hsa-miR-221 | CABYR |
| hsa-miR-221 | CADM1 |
| hsa-miR-221 | CAPN9 |
| hsa-miR-221 | CCDC126 |
| hsa-miR-221 | CCNC |
| hsa-miR-221 | CD4 |
| hsa-miR-221 | CDH23 |
| hsa-miR-221 | CEP170 |
| hsa-miR-221 | CPEB3 |
| hsa-miR-221 | CTSE |
| hsa-miR-221 | DICER1 |
| hsa-miR-221 | DIRAS2 |
| hsa-miR-221 | DLG2 |
| hsa-miR-221 | DNAH5 |
| hsa-miR-221 | DNAJC5 |
| hsa-miR-221 | ECHDC2 |
| hsa-miR-221 | EHF |
| hsa-miR-221 | ELL2 |
| hsa-miR-221 | FAM46A |
| hsa-miR-221 | FAT2 |
| hsa-miR-221 | FBLN1 |
| hsa-miR-221 | FBXO7 |
| hsa-miR-221 | FNDC3A |
| hsa-miR-221 | FOS |
| hsa-miR-221 | FOXRED1 |
| hsa-miR-221 | GALNT7 |
| hsa-miR-221 | GBGT1 |
| hsa-miR-221 | GOLPH3L |
| hsa-miR-221 | GPAM |
| hsa-miR-221 | GPD2 |
| hsa-miR-221 | GPR107 |
| hsa-miR-221 | GRIA4 |
| hsa-miR-221 | GULP1 |
| hsa-miR-221 | GYPC |
| hsa-miR-221 | HOXD1 |
| hsa-miR-221 | IDH1 |
| hsa-miR-221 | ITGB8 |
| hsa-miR-221 | ITSN1 |
| hsa-miR-221 | KCNK2 |
| hsa-miR-221 | KDR |
| hsa-miR-221 | KIF18A |
| hsa-miR-221 | KLC4 |
| hsa-miR-221 | KPNA2 |
| hsa-miR-221 | LGI2 |
| hsa-miR-221 | LRP10 |
| hsa-miR-221 | MAST2 |
| hsa-miR-221 | MDM4 |
| hsa-miR-221 | MPZL1 |
| hsa-miR-221 | MS4A1 |
| hsa-miR-221 | NFATC4 |
| hsa-miR-221 | NRSN1 |
| hsa-miR-221 | NUF2 |
| hsa-miR-221 | PANK3 |
| hsa-miR-221 | PCSK9 |
| hsa-miR-221 | PLEKHH2 |
| hsa-miR-221 | PLSCR4 |
| hsa-miR-221 | POGZ |
| hsa-miR-221 | PROM2 |
| hsa-miR-221 | PRPS1 |
| hsa-miR-221 | RAD51 |
| hsa-miR-221 | RASGRP1 |
| hsa-miR-221 | RIBC1 |
| hsa-miR-221 | RTN1 |
| hsa-miR-221 | SEC24B |
| hsa-miR-221 | SFXN1 |
| hsa-miR-221 | SIM2 |
| hsa-miR-221 | SLC26A3 |
| hsa-miR-221 | SMYD2 |
| hsa-miR-221 | SPATA18 |
| hsa-miR-221 | ST6GAL1 |
| hsa-miR-221 | STAB2 |
| hsa-miR-221 | STYX |
| hsa-miR-221 | SULF1 |
| hsa-miR-221 | TACC1 |
| hsa-miR-221 | TBC1D22B |
| hsa-miR-221 | TFRC |
| hsa-miR-221 | THBS2 |
| hsa-miR-221 | TMED5 |
| hsa-miR-221 | TMEM165 |
| hsa-miR-221 | TPD52L1 |
| hsa-miR-221 | TTF2 |
| hsa-miR-221 | VASH2 |
| hsa-miR-221 | ZFAND5 |
| hsa-miR-221 | ZNF547 |
| hsa-miR-221 | ZSWIM4 |
| hsa-miR-222 | ALDH1A1 |
| hsa-miR-222 | ANKRD10 |
| hsa-miR-222 | ARHGAP10 |
| hsa-miR-222 | CABYR |
| hsa-miR-222 | CD4 |
| hsa-miR-222 | CEP170 |
| hsa-miR-222 | CEP55 |
| hsa-miR-222 | CIT |
| hsa-miR-222 | CLDN12 |
| hsa-miR-222 | CREM |
| hsa-miR-222 | CTSE |
| hsa-miR-222 | DNMT3B |
| hsa-miR-222 | ERBB3 |
| hsa-miR-222 | FAM46A |
| hsa-miR-222 | FOS |
| hsa-miR-222 | GBGT1 |
| hsa-miR-222 | GOLGA4 |
| hsa-miR-222 | GPD2 |
| hsa-miR-222 | GPM6A |
| hsa-miR-222 | GPR107 |
| hsa-miR-222 | GRIA4 |
| hsa-miR-222 | HGF |
| hsa-miR-222 | HMGCLL1 |
| hsa-miR-222 | HOXD1 |
| hsa-miR-222 | KCNK2 |
| hsa-miR-222 | KCTD2 |
| hsa-miR-222 | KIAA1841 |
| hsa-miR-222 | KLC4 |
| hsa-miR-222 | LRP10 |
| hsa-miR-222 | MAPK10 |
| hsa-miR-222 | MAST2 |
| hsa-miR-222 | MDM4 |
| hsa-miR-222 | MPZL1 |
| hsa-miR-222 | NOVA1 |
| hsa-miR-222 | NRSN1 |
| hsa-miR-222 | NUF2 |
| hsa-miR-222 | PANK3 |
| hsa-miR-222 | PCDH7 |
| hsa-miR-222 | PFKFB2 |
| hsa-miR-222 | PLOD2 |
| hsa-miR-222 | POGZ |
| hsa-miR-222 | PTPRR |
| hsa-miR-222 | RASGRP1 |
| hsa-miR-222 | RIBC1 |
| hsa-miR-222 | SFXN1 |
| hsa-miR-222 | SNX4 |
| hsa-miR-222 | SPATA18 |
| hsa-miR-222 | SSFA2 |
| hsa-miR-222 | ST6GAL1 |
| hsa-miR-222 | STAB2 |
| hsa-miR-222 | SULF1 |
| hsa-miR-222 | TIAM1 |
| hsa-miR-222 | TMEM165 |
| hsa-miR-222 | TRAF2 |
| hsa-miR-222 | TUB |
| hsa-miR-222 | WDR35 |
| hsa-miR-222 | WWTR1 |
| hsa-miR-222 | ZFAND5 |
| hsa-miR-222 | ZNF547 |
| hsa-miR-222 | ZSWIM4 |
| hsa-miR-23a | AACS |
| hsa-miR-23a | ABCA5 |
| hsa-miR-23a | ABCC4 |
| hsa-miR-23a | ABI2 |
| hsa-miR-23a | ACAA1 |
| hsa-miR-23a | ADAM12 |
| hsa-miR-23a | ADAM28 |
| hsa-miR-23a | ANKRD29 |
| hsa-miR-23a | ARPC1B |
| hsa-miR-23a | ATP8B4 |
| hsa-miR-23a | C12orf29 |
| hsa-miR-23a | C9orf72 |
| hsa-miR-23a | CA2 |
| hsa-miR-23a | CAPN2 |
| hsa-miR-23a | CAPN6 |
| hsa-miR-23a | CBFA2T2 |
| hsa-miR-23a | CCDC126 |
| hsa-miR-23a | CCM2 |
| hsa-miR-23a | CD163 |
| hsa-miR-23a | CDH6 |
| hsa-miR-23a | CEACAM1 |
| hsa-miR-23a | CELSR1 |
| hsa-miR-23a | CEP68 |
| hsa-miR-23a | CLIP4 |
| hsa-miR-23a | COL4A1 |
| hsa-miR-23a | CREM |
| hsa-miR-23a | CTAGE5 |
| hsa-miR-23a | CYP2C18 |
| hsa-miR-23a | DCLK1 |
| hsa-miR-23a | DCUN1D4 |
| hsa-miR-23a | DGKH |
| hsa-miR-23a | DLC1 |
| hsa-miR-23a | DLX1 |
| hsa-miR-23a | DNAJC12 |
| hsa-miR-23a | DPP4 |
| hsa-miR-23a | DPP6 |
| hsa-miR-23a | DTNA |
| hsa-miR-23a | DZIP1 |
| hsa-miR-23a | E2F8 |
| hsa-miR-23a | EBF3 |
| hsa-miR-23a | EDA2R |
| hsa-miR-23a | EIF4ENIF1 |
| hsa-miR-23a | ELMO1 |
| hsa-miR-23a | ENO2 |
| hsa-miR-23a | ERBB4 |
| hsa-miR-23a | EXTL3 |
| hsa-miR-23a | FAM3B |
| hsa-miR-23a | FAM46A |
| hsa-miR-23a | FAM46C |
| hsa-miR-23a | FBXO7 |
| hsa-miR-23a | FDX1 |
| hsa-miR-23a | FGB |
| hsa-miR-23a | FNDC3A |
| hsa-miR-23a | GABRP |
| hsa-miR-23a | GALNT12 |
| hsa-miR-23a | GCAT |
| hsa-miR-23a | GCNT2 |
| hsa-miR-23a | GHITM |
| hsa-miR-23a | GOLPH3L |
| hsa-miR-23a | GOT1 |
| hsa-miR-23a | GP2 |
| hsa-miR-23a | GRIN2A |
| hsa-miR-23a | GSTCD |
| hsa-miR-23a | GTSE1 |
| hsa-miR-23a | HHIP |
| hsa-miR-23a | HMGCL |
| hsa-miR-23a | HOOK2 |
| hsa-miR-23a | IDH1 |
| hsa-miR-23a | IFT172 |
| hsa-miR-23a | IL1RAP |
| hsa-miR-23a | IL21R |
| hsa-miR-23a | IL6R |
| hsa-miR-23a | ILF3 |
| hsa-miR-23a | JAG1 |
| hsa-miR-23a | KALRN |
| hsa-miR-23a | KCNC4 |
| hsa-miR-23a | KHK |
| hsa-miR-23a | KIAA1841 |
| hsa-miR-23a | KIF25 |
| hsa-miR-23a | KLHL32 |
| hsa-miR-23a | LAMC2 |
| hsa-miR-23a | LIN7C |
| hsa-miR-23a | LMNB1 |
| hsa-miR-23a | LRIG1 |
| hsa-miR-23a | LRP12 |
| hsa-miR-23a | LRRFIP2 |
| hsa-miR-23a | MAP4K3 |
| hsa-miR-23a | MARK1 |
| hsa-miR-23a | MC1R |
| hsa-miR-23a | MCM4 |
| hsa-miR-23a | MCTP2 |
| hsa-miR-23a | MDFI |
| hsa-miR-23a | MINPP1 |
| hsa-miR-23a | MMAA |
| hsa-miR-23a | MT1X |
| hsa-miR-23a | MTM1 |
| hsa-miR-23a | MTSS1 |
| hsa-miR-23a | MYO5C |
| hsa-miR-23a | MYRIP |
| hsa-miR-23a | NCOA4 |
| hsa-miR-23a | NDRG4 |
| hsa-miR-23a | NEB |
| hsa-miR-23a | NHS |
| hsa-miR-23a | NRCAM |
| hsa-miR-23a | NRG1 |
| hsa-miR-23a | OIT3 |
| hsa-miR-23a | PAK6 |
| hsa-miR-23a | PFKFB4 |
| hsa-miR-23a | PKHD1 |
| hsa-miR-23a | PKP2 |
| hsa-miR-23a | PLA2G4C |
| hsa-miR-23a | PLEKHH2 |
| hsa-miR-23a | PROM2 |
| hsa-miR-23a | PRTFDC1 |
| hsa-miR-23a | RAD23B |
| hsa-miR-23a | REPS1 |
| hsa-miR-23a | RIMS2 |
| hsa-miR-23a | SACS |
| hsa-miR-23a | SALL2 |
| hsa-miR-23a | SATB1 |
| hsa-miR-23a | SFXN2 |
| hsa-miR-23a | SFXN5 |
| hsa-miR-23a | SH2D3A |
| hsa-miR-23a | SIGLEC1 |
| hsa-miR-23a | SIM2 |
| hsa-miR-23a | SIX4 |
| hsa-miR-23a | SLC12A1 |
| hsa-miR-23a | SLC17A2 |
| hsa-miR-23a | SLC20A1 |
| hsa-miR-23a | SLC22A5 |
| hsa-miR-23a | SLC25A27 |
| hsa-miR-23a | SLC25A30 |
| hsa-miR-23a | SLC25A36 |
| hsa-miR-23a | SLC39A14 |
| hsa-miR-23a | SMARCA1 |
| hsa-miR-23a | SNRK |
| hsa-miR-23a | SOD1 |
| hsa-miR-23a | STX6 |
| hsa-miR-23a | SYNE1 |
| hsa-miR-23a | SYNGAP1 |
| hsa-miR-23a | TCERG1 |
| hsa-miR-23a | TFPI2 |
| hsa-miR-23a | TJP2 |
| hsa-miR-23a | TMEM144 |
| hsa-miR-23a | TMEM164 |
| hsa-miR-23a | TMEM26 |
| hsa-miR-23a | TNC |
| hsa-miR-23a | TRIB1 |
| hsa-miR-23a | TTC7B |
| hsa-miR-23a | TTLL3 |
| hsa-miR-23a | USP30 |
| hsa-miR-23a | VIPR1 |
| hsa-miR-23a | VLDLR |
| hsa-miR-23a | VNN1 |
| hsa-miR-23a | WDR7 |
| hsa-miR-23a | WNK3 |
| hsa-miR-23a | ZBTB26 |
| hsa-miR-23a | ZCCHC2 |
| hsa-miR-23a | ZFP3 |
| hsa-miR-23a | ZNF195 |
| hsa-miR-23a | ZNF430 |
| hsa-miR-23a | ZNF474 |
| hsa-miR-23a | ZNF496 |
| hsa-miR-23a | ZNF514 |
| hsa-miR-23a | ZNF608 |
| hsa-miR-23a | ZNF669 |
| hsa-miR-23a | ZNF793 |
| hsa-miR-23a | ZYG11B |
| hsa-miR-27a | ABCA1 |
| hsa-miR-27a | ABCA3 |
| hsa-miR-27a | ABCB5 |
| hsa-miR-27a | ABCB6 |
| hsa-miR-27a | ABCB9 |
| hsa-miR-27a | ABCC4 |
| hsa-miR-27a | ABHD6 |
| hsa-miR-27a | ACLY |
| hsa-miR-27a | ADAM9 |
| hsa-miR-27a | ADAMTS10 |
| hsa-miR-27a | ADCY6 |
| hsa-miR-27a | AFAP1L2 |
| hsa-miR-27a | AFMID |
| hsa-miR-27a | AHSG |
| hsa-miR-27a | AICDA |
| hsa-miR-27a | AKR7A2 |
| hsa-miR-27a | ALDH4A1 |
| hsa-miR-27a | ALDH5A1 |
| hsa-miR-27a | ANKDD1A |
| hsa-miR-27a | ANKS6 |
| hsa-miR-27a | APBA2 |
| hsa-miR-27a | ARMC4 |
| hsa-miR-27a | ATF3 |
| hsa-miR-27a | BACE1 |
| hsa-miR-27a | BAG2 |
| hsa-miR-27a | BAZ2A |
| hsa-miR-27a | BLM |
| hsa-miR-27a | BMPER |
| hsa-miR-27a | CACNB2 |
| hsa-miR-27a | CADM1 |
| hsa-miR-27a | CAPN7 |
| hsa-miR-27a | CCM2 |
| hsa-miR-27a | CDADC1 |
| hsa-miR-27a | CECR2 |
| hsa-miR-27a | CLIP4 |
| hsa-miR-27a | COL16A1 |
| hsa-miR-27a | COL5A3 |
| hsa-miR-27a | CRMP1 |
| hsa-miR-27a | CUBN |
| hsa-miR-27a | CYP24A1 |
| hsa-miR-27a | DCLK2 |
| hsa-miR-27a | DKK2 |
| hsa-miR-27a | DLC1 |
| hsa-miR-27a | DMKN |
| hsa-miR-27a | DNAH5 |
| hsa-miR-27a | DNAJC16 |
| hsa-miR-27a | DNAJC5 |
| hsa-miR-27a | DNAJC6 |
| hsa-miR-27a | DNMT3A |
| hsa-miR-27a | DPYD |
| hsa-miR-27a | DPYS |
| hsa-miR-27a | E2F5 |
| hsa-miR-27a | EDIL3 |
| hsa-miR-27a | ELMO1 |
| hsa-miR-27a | ETV7 |
| hsa-miR-27a | FAM57A |
| hsa-miR-27a | FAS |
| hsa-miR-27a | FAT2 |
| hsa-miR-27a | FBLN5 |
| hsa-miR-27a | FCRL6 |
| hsa-miR-27a | FNDC4 |
| hsa-miR-27a | GAD2 |
| hsa-miR-27a | GALNT10 |
| hsa-miR-27a | GALNT3 |
| hsa-miR-27a | GALNT7 |
| hsa-miR-27a | GATA2 |
| hsa-miR-27a | GBGT1 |
| hsa-miR-27a | GCNT2 |
| hsa-miR-27a | GDA |
| hsa-miR-27a | GEMIN8 |
| hsa-miR-27a | GFM1 |
| hsa-miR-27a | GFPT2 |
| hsa-miR-27a | GLYATL1 |
| hsa-miR-27a | GPAM |
| hsa-miR-27a | GPATCH2 |
| hsa-miR-27a | GPC6 |
| hsa-miR-27a | GRIA4 |
| hsa-miR-27a | GRP |
| hsa-miR-27a | HAPLN1 |
| hsa-miR-27a | HDAC9 |
| hsa-miR-27a | HDHD3 |
| hsa-miR-27a | HKDC1 |
| hsa-miR-27a | HSH2D |
| hsa-miR-27a | IL13RA1 |
| hsa-miR-27a | IL1RAP |
| hsa-miR-27a | ITGA5 |
| hsa-miR-27a | ITSN1 |
| hsa-miR-27a | JAG1 |
| hsa-miR-27a | JPH1 |
| hsa-miR-27a | KALRN |
| hsa-miR-27a | KCNJ10 |
| hsa-miR-27a | KIF23 |
| hsa-miR-27a | KNDC1 |
| hsa-miR-27a | LAPTM4B |
| hsa-miR-27a | LDLR |
| hsa-miR-27a | LDLRAP1 |
| hsa-miR-27a | LGALS3 |
| hsa-miR-27a | LGR4 |
| hsa-miR-27a | LIN7C |
| hsa-miR-27a | LPIN1 |
| hsa-miR-27a | LRP1 |
| hsa-miR-27a | LRP12 |
| hsa-miR-27a | MAP1B |
| hsa-miR-27a | MAP2 |
| hsa-miR-27a | MCCC2 |
| hsa-miR-27a | MDH1B |
| hsa-miR-27a | MEGF6 |
| hsa-miR-27a | MINPP1 |
| hsa-miR-27a | MLKL |
| hsa-miR-27a | MMD |
| hsa-miR-27a | MND1 |
| hsa-miR-27a | MRPS25 |
| hsa-miR-27a | MS4A7 |
| hsa-miR-27a | MTMR4 |
| hsa-miR-27a | MYB |
| hsa-miR-27a | NEK6 |
| hsa-miR-27a | NHS |
| hsa-miR-27a | NOVA1 |
| hsa-miR-27a | NOX5 |
| hsa-miR-27a | NR1I2 |
| hsa-miR-27a | NSD1 |
| hsa-miR-27a | OBSCN |
| hsa-miR-27a | OIT3 |
| hsa-miR-27a | PAK6 |
| hsa-miR-27a | PANK1 |
| hsa-miR-27a | PAPPA |
| hsa-miR-27a | PDHX |
| hsa-miR-27a | PDLIM2 |
| hsa-miR-27a | PDLIM4 |
| hsa-miR-27a | PFKFB4 |
| hsa-miR-27a | PHF7 |
| hsa-miR-27a | PHGDH |
| hsa-miR-27a | PIK3R1 |
| hsa-miR-27a | PMAIP1 |
| hsa-miR-27a | PPIF |
| hsa-miR-27a | PPIH |
| hsa-miR-27a | PPM1K |
| hsa-miR-27a | PRKG2 |
| hsa-miR-27a | PROCR |
| hsa-miR-27a | PROZ |
| hsa-miR-27a | RAB27A |
| hsa-miR-27a | RBP5 |
| hsa-miR-27a | RET |
| hsa-miR-27a | RIC8B |
| hsa-miR-27a | RND3 |
| hsa-miR-27a | RNF182 |
| hsa-miR-27a | RUNX1 |
| hsa-miR-27a | SETD4 |
| hsa-miR-27a | SETD7 |
| hsa-miR-27a | SHF |
| hsa-miR-27a | SIGLEC1 |
| hsa-miR-27a | SLC17A3 |
| hsa-miR-27a | SLC25A25 |
| hsa-miR-27a | SLC26A8 |
| hsa-miR-27a | SLIT1 |
| hsa-miR-27a | SNCG |
| hsa-miR-27a | ST14 |
| hsa-miR-27a | STAB2 |
| hsa-miR-27a | STAT1 |
| hsa-miR-27a | STYK1 |
| hsa-miR-27a | SYDE1 |
| hsa-miR-27a | SYK |
| hsa-miR-27a | SYNE1 |
| hsa-miR-27a | TEK |
| hsa-miR-27a | TESK2 |
| hsa-miR-27a | THBS2 |
| hsa-miR-27a | TINAG |
| hsa-miR-27a | TJP2 |
| hsa-miR-27a | TMEM123 |
| hsa-miR-27a | TMEM25 |
| hsa-miR-27a | TMEM52 |
| hsa-miR-27a | TMPRSS7 |
| hsa-miR-27a | TMTC1 |
| hsa-miR-27a | TRIM24 |
| hsa-miR-27a | TRIM36 |
| hsa-miR-27a | TSFM |
| hsa-miR-27a | VANGL1 |
| hsa-miR-27a | VASH2 |
| hsa-miR-27a | VCAN |
| hsa-miR-27a | VPS45 |
| hsa-miR-27a | WISP1 |
| hsa-miR-27a | WISP3 |
| hsa-miR-27a | WNT2B |
| hsa-miR-27a | ZCCHC2 |
| hsa-miR-27a | ZFP36L1 |
| hsa-miR-27a | ZNF292 |
| hsa-miR-301a | ACAD10 |
| hsa-miR-301a | ADAMTS18 |
| hsa-miR-301a | AHSA2 |
| hsa-miR-301a | AKAP1 |
| hsa-miR-301a | AP1G2 |
| hsa-miR-301a | BTBD3 |
| hsa-miR-301a | C1S |
| hsa-miR-301a | C5orf30 |
| hsa-miR-301a | CEP55 |
| hsa-miR-301a | CFL2 |
| hsa-miR-301a | DLG5 |
| hsa-miR-301a | DNAJC16 |
| hsa-miR-301a | E2F2 |
| hsa-miR-301a | F3 |
| hsa-miR-301a | FAH |
| hsa-miR-301a | FANCA |
| hsa-miR-301a | FMO5 |
| hsa-miR-301a | GIPR |
| hsa-miR-301a | GPT2 |
| hsa-miR-301a | HBS1L |
| hsa-miR-301a | HIF1A |
| hsa-miR-301a | KLRF1 |
| hsa-miR-301a | LAMA3 |
| hsa-miR-301a | MS4A1 |
| hsa-miR-301a | NPEPL1 |
| hsa-miR-301a | NPNT |
| hsa-miR-301a | NUDT12 |
| hsa-miR-301a | PKHD1 |
| hsa-miR-301a | PLCB1 |
| hsa-miR-301a | PPP1R13L |
| hsa-miR-301a | PPP2R1B |
| hsa-miR-301a | PTGER2 |
| hsa-miR-301a | RAB34 |
| hsa-miR-301a | RFWD3 |
| hsa-miR-301a | RIBC1 |
| hsa-miR-301a | RUFY2 |
| hsa-miR-301a | SLAIN1 |
| hsa-miR-301a | SLC22A15 |
| hsa-miR-301a | SMYD2 |
| hsa-miR-301a | ST3GAL6 |
| hsa-miR-301a | STX6 |
| hsa-miR-301a | SYT10 |
| hsa-miR-301a | TEK |
| hsa-miR-301a | TMEM132A |
| hsa-miR-301a | TMEM55A |
| hsa-miR-301a | TRAPPC4 |
| hsa-miR-301a | UROD |
| hsa-miR-301a | ZDHHC7 |
| hsa-miR-30a | ABCA6 |
| hsa-miR-30a | ABCB5 |
| hsa-miR-30a | ADSSL1 |
| hsa-miR-30a | ANKRD10 |
| hsa-miR-30a | ANKS6 |
| hsa-miR-30a | AP3M2 |
| hsa-miR-30a | ARHGAP10 |
| hsa-miR-30a | ARL6IP6 |
| hsa-miR-30a | ATP11C |
| hsa-miR-30a | BCL9 |
| hsa-miR-30a | C12orf29 |
| hsa-miR-30a | C7orf31 |
| hsa-miR-30a | C9orf72 |
| hsa-miR-30a | CACNA1D |
| hsa-miR-30a | CACNB2 |
| hsa-miR-30a | CADPS |
| hsa-miR-30a | CAPN7 |
| hsa-miR-30a | CCM2 |
| hsa-miR-30a | CCNF |
| hsa-miR-30a | CELSR3 |
| hsa-miR-30a | CHL1 |
| hsa-miR-30a | CIT |
| hsa-miR-30a | CRMP1 |
| hsa-miR-30a | DACH2 |
| hsa-miR-30a | 1-Dec |
| hsa-miR-30a | DLG5 |
| hsa-miR-30a | DLGAP1 |
| hsa-miR-30a | DNAH5 |
| hsa-miR-30a | DOCK4 |
| hsa-miR-30a | EBF3 |
| hsa-miR-30a | FAM81B |
| hsa-miR-30a | GABRE |
| hsa-miR-30a | GALNT3 |
| hsa-miR-30a | GAS8 |
| hsa-miR-30a | GFPT2 |
| hsa-miR-30a | GLT1D1 |
| hsa-miR-30a | GPATCH2 |
| hsa-miR-30a | GRAMD1C |
| hsa-miR-30a | GRIA3 |
| hsa-miR-30a | GULP1 |
| hsa-miR-30a | IL21R |
| hsa-miR-30a | ITGA2 |
| hsa-miR-30a | KIF23 |
| hsa-miR-30a | LAMA1 |
| hsa-miR-30a | LIN7C |
| hsa-miR-30a | LRP12 |
| hsa-miR-30a | LRRC3 |
| hsa-miR-30a | LRRFIP2 |
| hsa-miR-30a | MARK1 |
| hsa-miR-30a | MDN1 |
| hsa-miR-30a | MOCS2 |
| hsa-miR-30a | MSI2 |
| hsa-miR-30a | MTHFD1 |
| hsa-miR-30a | OSBP2 |
| hsa-miR-30a | OXNAD1 |
| hsa-miR-30a | P4HA2 |
| hsa-miR-30a | PAPPA2 |
| hsa-miR-30a | PIK3R4 |
| hsa-miR-30a | PRKAG2 |
| hsa-miR-30a | PRRT2 |
| hsa-miR-30a | RAPH1 |
| hsa-miR-30a | RCE1 |
| hsa-miR-30a | RHEBL1 |
| hsa-miR-30a | SATB1 |
| hsa-miR-30a | SCARA5 |
| hsa-miR-30a | SH3GL3 |
| hsa-miR-30a | SLC22A5 |
| hsa-miR-30a | SLC25A30 |
| hsa-miR-30a | SLC25A36 |
| hsa-miR-30a | SLC44A3 |
| hsa-miR-30a | SLC7A6 |
| hsa-miR-30a | SMARCD1 |
| hsa-miR-30a | SNRK |
| hsa-miR-30a | SOD2 |
| hsa-miR-30a | SPDYA |
| hsa-miR-30a | SSTR2 |
| hsa-miR-30a | STAT1 |
| hsa-miR-30a | STX6 |
| hsa-miR-30a | SYNE1 |
| hsa-miR-30a | THBS2 |
| hsa-miR-30a | TMEM87B |
| hsa-miR-30a | TRIO |
| hsa-miR-30a | TRO |
| hsa-miR-30a | UAP1L1 |
| hsa-miR-30a | VCAN |
| hsa-miR-30a | WDR47 |
| hsa-miR-30a | WNK3 |
| hsa-miR-30a | YWHAZ |
| hsa-miR-30a | ZIK1 |
| hsa-miR-30a | ZNF275 |
| hsa-miR-30a | ZNF488 |
| hsa-miR-30a | ZYG11B |
| hsa-miR-30b | ABCA6 |
| hsa-miR-30b | ABCB5 |
| hsa-miR-30b | ABCC10 |
| hsa-miR-30b | ABCC5 |
| hsa-miR-30b | ABCC9 |
| hsa-miR-30b | ACP2 |
| hsa-miR-30b | ADAM12 |
| hsa-miR-30b | ADAM9 |
| hsa-miR-30b | ADSSL1 |
| hsa-miR-30b | AFAP1L2 |
| hsa-miR-30b | ALDH2 |
| hsa-miR-30b | ANKRD13D |
| hsa-miR-30b | ANKS6 |
| hsa-miR-30b | ARHGEF10 |
| hsa-miR-30b | ATG4A |
| hsa-miR-30b | ATP10A |
| hsa-miR-30b | BCL2L1 |
| hsa-miR-30b | BCL9 |
| hsa-miR-30b | BDKRB2 |
| hsa-miR-30b | C7orf31 |
| hsa-miR-30b | C9orf72 |
| hsa-miR-30b | CACNA1D |
| hsa-miR-30b | CACNB2 |
| hsa-miR-30b | CADM2 |
| hsa-miR-30b | CADPS |
| hsa-miR-30b | CARD14 |
| hsa-miR-30b | CCM2 |
| hsa-miR-30b | CCNJL |
| hsa-miR-30b | CD151 |
| hsa-miR-30b | CDC7 |
| hsa-miR-30b | CDCA7 |
| hsa-miR-30b | CDCA8 |
| hsa-miR-30b | CECR2 |
| hsa-miR-30b | CELSR3 |
| hsa-miR-30b | CFL2 |
| hsa-miR-30b | CHEK1 |
| hsa-miR-30b | CHI3L1 |
| hsa-miR-30b | CHL1 |
| hsa-miR-30b | CIT |
| hsa-miR-30b | COBLL1 |
| hsa-miR-30b | COL13A1 |
| hsa-miR-30b | CPEB3 |
| hsa-miR-30b | CREG1 |
| hsa-miR-30b | CREM |
| hsa-miR-30b | CRMP1 |
| hsa-miR-30b | CSNK1G1 |
| hsa-miR-30b | CTNND2 |
| hsa-miR-30b | CYB561 |
| hsa-miR-30b | CYP24A1 |
| hsa-miR-30b | DGKH |
| hsa-miR-30b | DLC1 |
| hsa-miR-30b | DLG5 |
| hsa-miR-30b | DLGAP1 |
| hsa-miR-30b | DNAH5 |
| hsa-miR-30b | DNAJC16 |
| hsa-miR-30b | DOLPP1 |
| hsa-miR-30b | DZIP1L |
| hsa-miR-30b | EBF2 |
| hsa-miR-30b | EBPL |
| hsa-miR-30b | EDIL3 |
| hsa-miR-30b | ELMO1 |
| hsa-miR-30b | EMB |
| hsa-miR-30b | EMP2 |
| hsa-miR-30b | EPHB2 |
| hsa-miR-30b | ERRFI1 |
| hsa-miR-30b | FAM126A |
| hsa-miR-30b | FAM81B |
| hsa-miR-30b | FAP |
| hsa-miR-30b | FGFR1 |
| hsa-miR-30b | FNDC3A |
| hsa-miR-30b | FOXRED1 |
| hsa-miR-30b | GALNT10 |
| hsa-miR-30b | GALNT3 |
| hsa-miR-30b | GALNT7 |
| hsa-miR-30b | GAS8 |
| hsa-miR-30b | GATM |
| hsa-miR-30b | GCLC |
| hsa-miR-30b | GDA |
| hsa-miR-30b | GLI2 |
| hsa-miR-30b | GLYAT |
| hsa-miR-30b | GNAO1 |
| hsa-miR-30b | GON4L |
| hsa-miR-30b | GPATCH2 |
| hsa-miR-30b | GRHL2 |
| hsa-miR-30b | GRIN2A |
| hsa-miR-30b | GULP1 |
| hsa-miR-30b | HADHB |
| hsa-miR-30b | HBS1L |
| hsa-miR-30b | HCN3 |
| hsa-miR-30b | HDAC9 |
| hsa-miR-30b | IL21R |
| hsa-miR-30b | IL6R |
| hsa-miR-30b | IQCK |
| hsa-miR-30b | ITGA5 |
| hsa-miR-30b | ITPK1 |
| hsa-miR-30b | IVNS1ABP |
| hsa-miR-30b | KALRN |
| hsa-miR-30b | KCTD7 |
| hsa-miR-30b | LAMA1 |
| hsa-miR-30b | LDB2 |
| hsa-miR-30b | LRP1 |
| hsa-miR-30b | LRP12 |
| hsa-miR-30b | LRRC3 |
| hsa-miR-30b | LRRFIP2 |
| hsa-miR-30b | MAP3K12 |
| hsa-miR-30b | MAST2 |
| hsa-miR-30b | MCTP2 |
| hsa-miR-30b | MDN1 |
| hsa-miR-30b | MICAL1 |
| hsa-miR-30b | MRPS22 |
| hsa-miR-30b | MS4A7 |
| hsa-miR-30b | MSI2 |
| hsa-miR-30b | MTSS1 |
| hsa-miR-30b | MYBL2 |
| hsa-miR-30b | MYD88 |
| hsa-miR-30b | MYLK |
| hsa-miR-30b | MYO1C |
| hsa-miR-30b | NCOR2 |
| hsa-miR-30b | NEDD4 |
| hsa-miR-30b | NHS |
| hsa-miR-30b | NID1 |
| hsa-miR-30b | NPAS2 |
| hsa-miR-30b | NR4A2 |
| hsa-miR-30b | NT5E |
| hsa-miR-30b | OSBP2 |
| hsa-miR-30b | OVOL1 |
| hsa-miR-30b | OXNAD1 |
| hsa-miR-30b | P4HA2 |
| hsa-miR-30b | PANK1 |
| hsa-miR-30b | PECR |
| hsa-miR-30b | PER3 |
| hsa-miR-30b | PFKFB2 |
| hsa-miR-30b | PFKFB4 |
| hsa-miR-30b | PIK3C2B |
| hsa-miR-30b | PIK3R4 |
| hsa-miR-30b | PITPNM3 |
| hsa-miR-30b | PML |
| hsa-miR-30b | PNKD |
| hsa-miR-30b | PPFIBP2 |
| hsa-miR-30b | PRKG2 |
| hsa-miR-30b | PRRT2 |
| hsa-miR-30b | PTGER2 |
| hsa-miR-30b | PTK7 |
| hsa-miR-30b | PTP4A1 |
| hsa-miR-30b | PTPRB |
| hsa-miR-30b | RAD54B |
| hsa-miR-30b | RAPGEF2 |
| hsa-miR-30b | RAPH1 |
| hsa-miR-30b | RARG |
| hsa-miR-30b | RCE1 |
| hsa-miR-30b | RHEBL1 |
| hsa-miR-30b | RNF44 |
| hsa-miR-30b | SACS |
| hsa-miR-30b | SAMD4A |
| hsa-miR-30b | SCMH1 |
| hsa-miR-30b | SCML2 |
| hsa-miR-30b | SEC24D |
| hsa-miR-30b | 2-Sep |
| hsa-miR-30b | 3-Sep |
| hsa-miR-30b | 8-Sep |
| hsa-miR-30b | SERPINE1 |
| hsa-miR-30b | SETD3 |
| hsa-miR-30b | SH3BP5L |
| hsa-miR-30b | SH3GL3 |
| hsa-miR-30b | SLC22A5 |
| hsa-miR-30b | SLC30A2 |
| hsa-miR-30b | SLC38A2 |
| hsa-miR-30b | SLC6A9 |
| hsa-miR-30b | SMARCD1 |
| hsa-miR-30b | SMYD2 |
| hsa-miR-30b | SNRK |
| hsa-miR-30b | SOCS3 |
| hsa-miR-30b | SSTR2 |
| hsa-miR-30b | STAT1 |
| hsa-miR-30b | STX6 |
| hsa-miR-30b | SYP |
| hsa-miR-30b | SYT9 |
| hsa-miR-30b | TEC |
| hsa-miR-30b | TEX14 |
| hsa-miR-30b | TFAP2A |
| hsa-miR-30b | THSD4 |
| hsa-miR-30b | TIMP2 |
| hsa-miR-30b | TMEM165 |
| hsa-miR-30b | TMEM26 |
| hsa-miR-30b | TMEM79 |
| hsa-miR-30b | TMTC1 |
| hsa-miR-30b | TNFRSF19 |
| hsa-miR-30b | TRIO |
| hsa-miR-30b | TRO |
| hsa-miR-30b | TRPC4 |
| hsa-miR-30b | UAP1L1 |
| hsa-miR-30b | USP2 |
| hsa-miR-30b | VANGL1 |
| hsa-miR-30b | VAV3 |
| hsa-miR-30b | VIPR1 |
| hsa-miR-30b | WDR47 |
| hsa-miR-30b | ZCCHC6 |
| hsa-miR-30b | ZDHHC14 |
| hsa-miR-30b | ZFAND5 |
| hsa-miR-30b | ZFP1 |
| hsa-miR-30b | ZFP3 |
| hsa-miR-30b | ZIK1 |
| hsa-miR-30b | ZNF233 |
| hsa-miR-30b | ZNF337 |
| hsa-miR-30b | ZNF488 |
| hsa-miR-30b | ZNF528 |
| hsa-miR-30b | ZNF547 |
| hsa-miR-30b | ZNF608 |
| hsa-miR-30d | ABCA6 |
| hsa-miR-30d | ABCB5 |
| hsa-miR-30d | ADAM10 |
| hsa-miR-30d | ALDH2 |
| hsa-miR-30d | ANKS6 |
| hsa-miR-30d | ARHGAP10 |
| hsa-miR-30d | ARL6IP6 |
| hsa-miR-30d | BCL9 |
| hsa-miR-30d | BZW2 |
| hsa-miR-30d | CACNA1D |
| hsa-miR-30d | CACNB2 |
| hsa-miR-30d | CADPS |
| hsa-miR-30d | CBS |
| hsa-miR-30d | CCM2 |
| hsa-miR-30d | CCNF |
| hsa-miR-30d | CD80 |
| hsa-miR-30d | CHL1 |
| hsa-miR-30d | CRMP1 |
| hsa-miR-30d | DLG5 |
| hsa-miR-30d | DLGAP1 |
| hsa-miR-30d | DNAH5 |
| hsa-miR-30d | DZIP1L |
| hsa-miR-30d | EBF3 |
| hsa-miR-30d | EPHB2 |
| hsa-miR-30d | FAM81B |
| hsa-miR-30d | FGFR1 |
| hsa-miR-30d | FNDC3A |
| hsa-miR-30d | GALNT3 |
| hsa-miR-30d | GDA |
| hsa-miR-30d | GFPT2 |
| hsa-miR-30d | GLT1D1 |
| hsa-miR-30d | GON4L |
| hsa-miR-30d | GRIA3 |
| hsa-miR-30d | GULP1 |
| hsa-miR-30d | KALRN |
| hsa-miR-30d | KIAA1841 |
| hsa-miR-30d | LAMA1 |
| hsa-miR-30d | LIN7C |
| hsa-miR-30d | MSI2 |
| hsa-miR-30d | MTA3 |
| hsa-miR-30d | OXNAD1 |
| hsa-miR-30d | P4HA2 |
| hsa-miR-30d | PANK1 |
| hsa-miR-30d | PDHX |
| hsa-miR-30d | PFKFB4 |
| hsa-miR-30d | PGM1 |
| hsa-miR-30d | PIK3R4 |
| hsa-miR-30d | PRRT2 |
| hsa-miR-30d | PTK7 |
| hsa-miR-30d | RAD23B |
| hsa-miR-30d | RAPH1 |
| hsa-miR-30d | RCE1 |
| hsa-miR-30d | RHEBL1 |
| hsa-miR-30d | RIC8B |
| hsa-miR-30d | SAMD4A |
| hsa-miR-30d | SATB1 |
| hsa-miR-30d | SCARA5 |
| hsa-miR-30d | SH3GL3 |
| hsa-miR-30d | SLC22A5 |
| hsa-miR-30d | SLC25A30 |
| hsa-miR-30d | SLC25A36 |
| hsa-miR-30d | SLC30A1 |
| hsa-miR-30d | SLC30A2 |
| hsa-miR-30d | SLC38A4 |
| hsa-miR-30d | SLC7A6 |
| hsa-miR-30d | SMARCD1 |
| hsa-miR-30d | SMYD2 |
| hsa-miR-30d | SNRK |
| hsa-miR-30d | STX6 |
| hsa-miR-30d | SYNE1 |
| hsa-miR-30d | TMEM26 |
| hsa-miR-30d | TMEM87B |
| hsa-miR-30d | TNFRSF19 |
| hsa-miR-30d | TRIO |
| hsa-miR-30d | UAP1L1 |
| hsa-miR-30d | VCAN |
| hsa-miR-30d | WDR47 |
| hsa-miR-30d | WDR76 |
| hsa-miR-30d | WNK3 |
| hsa-miR-30d | ZIK1 |
| hsa-miR-30d | ZNF275 |
| hsa-miR-30d | ZYG11B |
| hsa-miR-31 | ABCB9 |
| hsa-miR-31 | ABHD1 |
| hsa-miR-31 | ACAT1 |
| hsa-miR-31 | ADCY6 |
| hsa-miR-31 | AGT |
| hsa-miR-31 | AKR1B1 |
| hsa-miR-31 | ALDH1L2 |
| hsa-miR-31 | ALDOC |
| hsa-miR-31 | AMT |
| hsa-miR-31 | ASB13 |
| hsa-miR-31 | ATP11C |
| hsa-miR-31 | AVPI1 |
| hsa-miR-31 | AVPR2 |
| hsa-miR-31 | C15orf39 |
| hsa-miR-31 | C19orf12 |
| hsa-miR-31 | C8orf46 |
| hsa-miR-31 | CACNB3 |
| hsa-miR-31 | CCL23 |
| hsa-miR-31 | CCNC |
| hsa-miR-31 | CD1A |
| hsa-miR-31 | CD79B |
| hsa-miR-31 | CHST4 |
| hsa-miR-31 | CTNND2 |
| hsa-miR-31 | CTSC |
| hsa-miR-31 | CXorf36 |
| hsa-miR-31 | DBF4B |
| hsa-miR-31 | DBP |
| hsa-miR-31 | DHCR24 |
| hsa-miR-31 | DICER1 |
| hsa-miR-31 | DLX4 |
| hsa-miR-31 | DNAJC10 |
| hsa-miR-31 | DNASE1L3 |
| hsa-miR-31 | DPP4 |
| hsa-miR-31 | ERBB3 |
| hsa-miR-31 | FA2H |
| hsa-miR-31 | FBLN1 |
| hsa-miR-31 | FHAD1 |
| hsa-miR-31 | FIBCD1 |
| hsa-miR-31 | FMO5 |
| hsa-miR-31 | FMOD |
| hsa-miR-31 | FNDC4 |
| hsa-miR-31 | FPGS |
| hsa-miR-31 | GAS8 |
| hsa-miR-31 | GCK |
| hsa-miR-31 | GLI2 |
| hsa-miR-31 | GPHN |
| hsa-miR-31 | GRAMD1C |
| hsa-miR-31 | GSTCD |
| hsa-miR-31 | GTSE1 |
| hsa-miR-31 | HAAO |
| hsa-miR-31 | HADH |
| hsa-miR-31 | HAO2 |
| hsa-miR-31 | HIP1R |
| hsa-miR-31 | HLF |
| hsa-miR-31 | HPGD |
| hsa-miR-31 | HTR3A |
| hsa-miR-31 | IGSF11 |
| hsa-miR-31 | IL1R1 |
| hsa-miR-31 | ITGAV |
| hsa-miR-31 | ITIH5 |
| hsa-miR-31 | IVNS1ABP |
| hsa-miR-31 | KCNG3 |
| hsa-miR-31 | KCNK2 |
| hsa-miR-31 | KCNK9 |
| hsa-miR-31 | KHDRBS3 |
| hsa-miR-31 | KIAA0556 |
| hsa-miR-31 | KIAA0907 |
| hsa-miR-31 | KRT80 |
| hsa-miR-31 | LGR4 |
| hsa-miR-31 | LIMS2 |
| hsa-miR-31 | LOXL4 |
| hsa-miR-31 | MAP2 |
| hsa-miR-31 | MCM2 |
| hsa-miR-31 | MERTK |
| hsa-miR-31 | MORN3 |
| hsa-miR-31 | MYO18B |
| hsa-miR-31 | NEDD4 |
| hsa-miR-31 | NPAS2 |
| hsa-miR-31 | NPC1L1 |
| hsa-miR-31 | NUDT8 |
| hsa-miR-31 | OIT3 |
| hsa-miR-31 | OPRL1 |
| hsa-miR-31 | OSBP2 |
| hsa-miR-31 | OSBPL10 |
| hsa-miR-31 | OSBPL5 |
| hsa-miR-31 | PAFAH2 |
| hsa-miR-31 | PAPPA2 |
| hsa-miR-31 | PDGFC |
| hsa-miR-31 | PDHX |
| hsa-miR-31 | PEX19 |
| hsa-miR-31 | PKHD1 |
| hsa-miR-31 | PLCB1 |
| hsa-miR-31 | PML |
| hsa-miR-31 | PMP22 |
| hsa-miR-31 | PPP1R9A |
| hsa-miR-31 | PRDX3 |
| hsa-miR-31 | PRRT2 |
| hsa-miR-31 | PRSS8 |
| hsa-miR-31 | PTPRE |
| hsa-miR-31 | RAB27A |
| hsa-miR-31 | RHOC |
| hsa-miR-31 | RIBC1 |
| hsa-miR-31 | RND3 |
| hsa-miR-31 | RNF123 |
| hsa-miR-31 | RUSC1 |
| hsa-miR-31 | SEMA5B |
| hsa-miR-31 | SIDT2 |
| hsa-miR-31 | SLC16A2 |
| hsa-miR-31 | SLC25A27 |
| hsa-miR-31 | SLC39A13 |
| hsa-miR-31 | SLC39A14 |
| hsa-miR-31 | SLC43A2 |
| hsa-miR-31 | SLCO2B1 |
| hsa-miR-31 | SMARCD1 |
| hsa-miR-31 | SPSB2 |
| hsa-miR-31 | SRC |
| hsa-miR-31 | ST3GAL3 |
| hsa-miR-31 | STARD8 |
| hsa-miR-31 | STMN1 |
| hsa-miR-31 | TACC1 |
| hsa-miR-31 | TACC2 |
| hsa-miR-31 | TBX19 |
| hsa-miR-31 | TEK |
| hsa-miR-31 | TESK2 |
| hsa-miR-31 | THBS2 |
| hsa-miR-31 | TNIK |
| hsa-miR-31 | TREM2 |
| hsa-miR-31 | TRIM29 |
| hsa-miR-31 | TRIOBP |
| hsa-miR-31 | TRPV6 |
| hsa-miR-31 | TSPAN15 |
| hsa-miR-31 | TTC21A |
| hsa-miR-31 | UGT3A1 |
| hsa-miR-31 | USP2 |
| hsa-miR-31 | VAV3 |
| hsa-miR-31 | VSIG4 |
| hsa-miR-31 | WISP1 |
| hsa-miR-31 | ZNF19 |
| hsa-miR-31 | ZNF474 |
| hsa-miR-33b | ABCA1 |
| hsa-miR-33b | ABCB5 |
| hsa-miR-33b | ACAD8 |
| hsa-miR-33b | ADAM12 |
| hsa-miR-33b | ADRA1A |
| hsa-miR-33b | ADSS |
| hsa-miR-33b | ANKRD28 |
| hsa-miR-33b | ANKRD29 |
| hsa-miR-33b | ANTXR1 |
| hsa-miR-33b | APBA2 |
| hsa-miR-33b | APH1B |
| hsa-miR-33b | ARHGAP27 |
| hsa-miR-33b | ARMC3 |
| hsa-miR-33b | C1S |
| hsa-miR-33b | C21orf91 |
| hsa-miR-33b | C8B |
| hsa-miR-33b | CABYR |
| hsa-miR-33b | CADM1 |
| hsa-miR-33b | CAPN10 |
| hsa-miR-33b | CCM2 |
| hsa-miR-33b | CDADC1 |
| hsa-miR-33b | CDCA3 |
| hsa-miR-33b | CHN2 |
| hsa-miR-33b | COL4A1 |
| hsa-miR-33b | COL6A3 |
| hsa-miR-33b | CPOX |
| hsa-miR-33b | CS |
| hsa-miR-33b | CYP4A11 |
| hsa-miR-33b | DACH2 |
| hsa-miR-33b | DNAH9 |
| hsa-miR-33b | EI24 |
| hsa-miR-33b | EPHA7 |
| hsa-miR-33b | FAM102B |
| hsa-miR-33b | FKBP5 |
| hsa-miR-33b | FUT2 |
| hsa-miR-33b | GCLC |
| hsa-miR-33b | GPC6 |
| hsa-miR-33b | GPR132 |
| hsa-miR-33b | GPSM2 |
| hsa-miR-33b | GREM1 |
| hsa-miR-33b | GSTA4 |
| hsa-miR-33b | HADHB |
| hsa-miR-33b | HDAC11 |
| hsa-miR-33b | ITGA2 |
| hsa-miR-33b | KALRN |
| hsa-miR-33b | KCNQ5 |
| hsa-miR-33b | KIF23 |
| hsa-miR-33b | LAMA3 |
| hsa-miR-33b | LAMA4 |
| hsa-miR-33b | LGR4 |
| hsa-miR-33b | MBOAT2 |
| hsa-miR-33b | MEIS2 |
| hsa-miR-33b | MMP28 |
| hsa-miR-33b | MOGAT2 |
| hsa-miR-33b | MPDZ |
| hsa-miR-33b | MRPS25 |
| hsa-miR-33b | MRPS28 |
| hsa-miR-33b | MTMR11 |
| hsa-miR-33b | NOVA1 |
| hsa-miR-33b | OSBPL6 |
| hsa-miR-33b | PCK1 |
| hsa-miR-33b | PGM1 |
| hsa-miR-33b | PKHD1 |
| hsa-miR-33b | PLVAP |
| hsa-miR-33b | PUS7 |
| hsa-miR-33b | RAD51 |
| hsa-miR-33b | RNF128 |
| hsa-miR-33b | SEL1L |
| hsa-miR-33b | SEMA5B |
| hsa-miR-33b | SFXN1 |
| hsa-miR-33b | SH3TC2 |
| hsa-miR-33b | SIPA1L2 |
| hsa-miR-33b | SIX4 |
| hsa-miR-33b | SLC13A1 |
| hsa-miR-33b | SLC22A15 |
| hsa-miR-33b | SLC25A25 |
| hsa-miR-33b | SLC2A5 |
| hsa-miR-33b | SLC4A10 |
| hsa-miR-33b | SLCO1A2 |
| hsa-miR-33b | SNRPB |
| hsa-miR-33b | SPATA18 |
| hsa-miR-33b | STEAP4 |
| hsa-miR-33b | SULF1 |
| hsa-miR-33b | SYNE1 |
| hsa-miR-33b | TBX15 |
| hsa-miR-33b | TCL1A |
| hsa-miR-33b | TFAP2A |
| hsa-miR-33b | TINAG |
| hsa-miR-33b | TMEM154 |
| hsa-miR-33b | TMEM87A |
| hsa-miR-33b | TPM3 |
| hsa-miR-33b | TTC13 |
| hsa-miR-33b | WDR7 |
| hsa-miR-33b | ZDHHC13 |
| hsa-miR-33b | ZDHHC14 |
| hsa-miR-33b | ZFAND5 |
| hsa-miR-33b | ZFP36L1 |
| hsa-miR-33b | ZNF496 |
| hsa-miR-346 | ALDH1L2 |
| hsa-miR-346 | ATP8B4 |
| hsa-miR-346 | CNNM1 |
| hsa-miR-346 | F7 |
| hsa-miR-346 | IL20RA |
| hsa-miR-346 | KCNC4 |
| hsa-miR-346 | NCDN |
| hsa-miR-346 | PAFAH2 |
| hsa-miR-346 | PPP1R9B |
| hsa-miR-346 | RIMS2 |
| hsa-miR-346 | 4-Sep |
| hsa-miR-346 | ZDHHC13 |
| hsa-miR-383 | ABCA1 |
| hsa-miR-383 | ABCC11 |
| hsa-miR-383 | ACLY |
| hsa-miR-383 | ADAMTS18 |
| hsa-miR-383 | ADIPOR2 |
| hsa-miR-383 | ADSS |
| hsa-miR-383 | ANKDD1A |
| hsa-miR-383 | ANXA5 |
| hsa-miR-383 | ARHGAP27 |
| hsa-miR-383 | ARHGEF18 |
| hsa-miR-383 | ASB9 |
| hsa-miR-383 | BARD1 |
| hsa-miR-383 | BCKDK |
| hsa-miR-383 | BPHL |
| hsa-miR-383 | CALCA |
| hsa-miR-383 | CAPN3 |
| hsa-miR-383 | CAPN6 |
| hsa-miR-383 | CEP250 |
| hsa-miR-383 | CHST10 |
| hsa-miR-383 | CLDN12 |
| hsa-miR-383 | CNNM1 |
| hsa-miR-383 | CYP27B1 |
| hsa-miR-383 | DCLK1 |
| hsa-miR-383 | DGKA |
| hsa-miR-383 | DIAPH3 |
| hsa-miR-383 | DYNLRB2 |
| hsa-miR-383 | DZIP1L |
| hsa-miR-383 | ELMO1 |
| hsa-miR-383 | FBLN1 |
| hsa-miR-383 | FNDC3A |
| hsa-miR-383 | GABARAPL1 |
| hsa-miR-383 | GADD45G |
| hsa-miR-383 | GRIA3 |
| hsa-miR-383 | GSTCD |
| hsa-miR-383 | GTSE1 |
| hsa-miR-383 | HDAC9 |
| hsa-miR-383 | IGF1R |
| hsa-miR-383 | IL1RAP |
| hsa-miR-383 | IVNS1ABP |
| hsa-miR-383 | KCNE1 |
| hsa-miR-383 | KCNG3 |
| hsa-miR-383 | KCNJ10 |
| hsa-miR-383 | LGI2 |
| hsa-miR-383 | LGR4 |
| hsa-miR-383 | LYPD5 |
| hsa-miR-383 | MAPT |
| hsa-miR-383 | MCTP2 |
| hsa-miR-383 | MORN3 |
| hsa-miR-383 | MTM1 |
| hsa-miR-383 | MTMR4 |
| hsa-miR-383 | MYLK |
| hsa-miR-383 | MYO18B |
| hsa-miR-383 | NOVA1 |
| hsa-miR-383 | OIP5 |
| hsa-miR-383 | OSBPL6 |
| hsa-miR-383 | PEX19 |
| hsa-miR-383 | PIAS3 |
| hsa-miR-383 | PIK3R1 |
| hsa-miR-383 | PLA1A |
| hsa-miR-383 | PMFBP1 |
| hsa-miR-383 | POGK |
| hsa-miR-383 | PPP1R9A |
| hsa-miR-383 | PRDX3 |
| hsa-miR-383 | PTPRB |
| hsa-miR-383 | PYCARD |
| hsa-miR-383 | RAPGEF2 |
| hsa-miR-383 | RBM3 |
| hsa-miR-383 | SACS |
| hsa-miR-383 | SCD |
| hsa-miR-383 | SERPINA3 |
| hsa-miR-383 | SHMT2 |
| hsa-miR-383 | SLC13A3 |
| hsa-miR-383 | SLC2A4 |
| hsa-miR-383 | SLC39A13 |
| hsa-miR-383 | SLC6A2 |
| hsa-miR-383 | SOCS7 |
| hsa-miR-383 | SORCS1 |
| hsa-miR-383 | SYNE1 |
| hsa-miR-383 | TIMM8A |
| hsa-miR-383 | TJP2 |
| hsa-miR-383 | TMEM45B |
| hsa-miR-383 | TRPC6 |
| hsa-miR-383 | TTC7B |
| hsa-miR-383 | TTLL3 |
| hsa-miR-383 | VPS72 |
| hsa-miR-383 | ZNF300 |
| hsa-miR-383 | ZNF605 |
| hsa-miR-424 | AADAT |
| hsa-miR-424 | ABCC10 |
| hsa-miR-424 | ABCC5 |
| hsa-miR-424 | ABHD1 |
| hsa-miR-424 | ABHD5 |
| hsa-miR-424 | ABI2 |
| hsa-miR-424 | ACAD10 |
| hsa-miR-424 | ACBD5 |
| hsa-miR-424 | ACOX3 |
| hsa-miR-424 | ACSBG2 |
| hsa-miR-424 | ACSS2 |
| hsa-miR-424 | ADAM12 |
| hsa-miR-424 | ADAMTS18 |
| hsa-miR-424 | ADD2 |
| hsa-miR-424 | ALB |
| hsa-miR-424 | ALDH2 |
| hsa-miR-424 | ALDOA |
| hsa-miR-424 | AMT |
| hsa-miR-424 | ANKRD13B |
| hsa-miR-424 | ANKRD29 |
| hsa-miR-424 | AP2B1 |
| hsa-miR-424 | ARHGAP22 |
| hsa-miR-424 | ARHGEF1 |
| hsa-miR-424 | ASTN1 |
| hsa-miR-424 | ATP10B |
| hsa-miR-424 | ATP13A2 |
| hsa-miR-424 | AURKAIP1 |
| hsa-miR-424 | B3GALNT1 |
| hsa-miR-424 | BCL7A |
| hsa-miR-424 | BCL9 |
| hsa-miR-424 | BMPER |
| hsa-miR-424 | BPHL |
| hsa-miR-424 | C1QB |
| hsa-miR-424 | CCDC25 |
| hsa-miR-424 | CCM2 |
| hsa-miR-424 | CCNE2 |
| hsa-miR-424 | CCNJL |
| hsa-miR-424 | CD151 |
| hsa-miR-424 | CD163 |
| hsa-miR-424 | CD1D |
| hsa-miR-424 | CD4 |
| hsa-miR-424 | CD80 |
| hsa-miR-424 | CDC25A |
| hsa-miR-424 | CDCA4 |
| hsa-miR-424 | CHRM3 |
| hsa-miR-424 | CHRNA4 |
| hsa-miR-424 | CHRNB4 |
| hsa-miR-424 | CLCN5 |
| hsa-miR-424 | CLDN12 |
| hsa-miR-424 | CLDN2 |
| hsa-miR-424 | CLIP4 |
| hsa-miR-424 | CLU |
| hsa-miR-424 | CMTM1 |
| hsa-miR-424 | COCH |
| hsa-miR-424 | COL1A2 |
| hsa-miR-424 | COL3A1 |
| hsa-miR-424 | COL4A1 |
| hsa-miR-424 | CPEB4 |
| hsa-miR-424 | CRIM1 |
| hsa-miR-424 | CSMD2 |
| hsa-miR-424 | CYP46A1 |
| hsa-miR-424 | DBF4B |
| hsa-miR-424 | DCUN1D4 |
| hsa-miR-424 | DENND2C |
| hsa-miR-424 | DGKH |
| hsa-miR-424 | DICER1 |
| hsa-miR-424 | DNAH3 |
| hsa-miR-424 | DNAJA4 |
| hsa-miR-424 | DNAJC10 |
| hsa-miR-424 | DNAJC5 |
| hsa-miR-424 | DNMT3B |
| hsa-miR-424 | DZIP1 |
| hsa-miR-424 | ELOVL7 |
| hsa-miR-424 | ENPP3 |
| hsa-miR-424 | EPHA7 |
| hsa-miR-424 | ERBB4 |
| hsa-miR-424 | ESR1 |
| hsa-miR-424 | FABP1 |
| hsa-miR-424 | FADS1 |
| hsa-miR-424 | FAM57B |
| hsa-miR-424 | FBXO43 |
| hsa-miR-424 | FCHO1 |
| hsa-miR-424 | FECH |
| hsa-miR-424 | FGL1 |
| hsa-miR-424 | FHOD3 |
| hsa-miR-424 | FKBP5 |
| hsa-miR-424 | FOXRED1 |
| hsa-miR-424 | FRMPD1 |
| hsa-miR-424 | FUT3 |
| hsa-miR-424 | GABRP |
| hsa-miR-424 | GADD45G |
| hsa-miR-424 | GALNT12 |
| hsa-miR-424 | GIPR |
| hsa-miR-424 | GLS2 |
| hsa-miR-424 | GOLGA4 |
| hsa-miR-424 | GOT2 |
| hsa-miR-424 | GPAM |
| hsa-miR-424 | GPC6 |
| hsa-miR-424 | GPD2 |
| hsa-miR-424 | GPR180 |
| hsa-miR-424 | GYPC |
| hsa-miR-424 | HADH |
| hsa-miR-424 | HECTD1 |
| hsa-miR-424 | HIGD1A |
| hsa-miR-424 | HK1 |
| hsa-miR-424 | HPGD |
| hsa-miR-424 | HTR3A |
| hsa-miR-424 | IFT57 |
| hsa-miR-424 | IL6R |
| hsa-miR-424 | ILDR1 |
| hsa-miR-424 | INVS |
| hsa-miR-424 | ITGA2 |
| hsa-miR-424 | ITIH1 |
| hsa-miR-424 | ITIH5 |
| hsa-miR-424 | IVNS1ABP |
| hsa-miR-424 | JAK3 |
| hsa-miR-424 | KALRN |
| hsa-miR-424 | KCNC4 |
| hsa-miR-424 | KCNK2 |
| hsa-miR-424 | KCNN4 |
| hsa-miR-424 | KCNS1 |
| hsa-miR-424 | KCNT2 |
| hsa-miR-424 | KDR |
| hsa-miR-424 | KIAA0100 |
| hsa-miR-424 | KIAA0907 |
| hsa-miR-424 | KIAA1324 |
| hsa-miR-424 | KLC4 |
| hsa-miR-424 | KREMEN2 |
| hsa-miR-424 | LGI2 |
| hsa-miR-424 | LGI4 |
| hsa-miR-424 | LGR6 |
| hsa-miR-424 | LPIN1 |
| hsa-miR-424 | LRRC1 |
| hsa-miR-424 | LRRC27 |
| hsa-miR-424 | LRRFIP2 |
| hsa-miR-424 | LRRK1 |
| hsa-miR-424 | LY6E |
| hsa-miR-424 | MDGA1 |
| hsa-miR-424 | METTL9 |
| hsa-miR-424 | MFN2 |
| hsa-miR-424 | MMAA |
| hsa-miR-424 | MOCS1 |
| hsa-miR-424 | MST1R |
| hsa-miR-424 | MT1X |
| hsa-miR-424 | MTCH2 |
| hsa-miR-424 | MTMR10 |
| hsa-miR-424 | MTMR11 |
| hsa-miR-424 | MTMR4 |
| hsa-miR-424 | MYLK |
| hsa-miR-424 | MYO1C |
| hsa-miR-424 | NAV1 |
| hsa-miR-424 | NDP |
| hsa-miR-424 | NEK10 |
| hsa-miR-424 | NEK11 |
| hsa-miR-424 | NEO1 |
| hsa-miR-424 | NOD1 |
| hsa-miR-424 | NOL3 |
| hsa-miR-424 | NPR2 |
| hsa-miR-424 | NRG1 |
| hsa-miR-424 | OR51E1 |
| hsa-miR-424 | OXNAD1 |
| hsa-miR-424 | PACS1 |
| hsa-miR-424 | PANK1 |
| hsa-miR-424 | PGM1 |
| hsa-miR-424 | PIK3R1 |
| hsa-miR-424 | PJA1 |
| hsa-miR-424 | PMP22 |
| hsa-miR-424 | PNPLA7 |
| hsa-miR-424 | PNPO |
| hsa-miR-424 | POLD3 |
| hsa-miR-424 | PPARGC1A |
| hsa-miR-424 | PPFIA4 |
| hsa-miR-424 | PPIF |
| hsa-miR-424 | PPP2R1B |
| hsa-miR-424 | PTK7 |
| hsa-miR-424 | PTPN3 |
| hsa-miR-424 | QPRT |
| hsa-miR-424 | RAB11FIP1 |
| hsa-miR-424 | RAB37 |
| hsa-miR-424 | RAD23B |
| hsa-miR-424 | RAD51 |
| hsa-miR-424 | RAF1 |
| hsa-miR-424 | RASGEF1B |
| hsa-miR-424 | RASSF4 |
| hsa-miR-424 | RBPMS |
| hsa-miR-424 | RCE1 |
| hsa-miR-424 | RCL1 |
| hsa-miR-424 | RCN2 |
| hsa-miR-424 | RDH13 |
| hsa-miR-424 | RET |
| hsa-miR-424 | RFWD3 |
| hsa-miR-424 | RHBG |
| hsa-miR-424 | RIC8B |
| hsa-miR-424 | RNASE7 |
| hsa-miR-424 | RNF152 |
| hsa-miR-424 | RTN1 |
| hsa-miR-424 | SACM1L |
| hsa-miR-424 | SACS |
| hsa-miR-424 | SAT1 |
| hsa-miR-424 | SCAMP3 |
| hsa-miR-424 | SCARB1 |
| hsa-miR-424 | SCIN |
| hsa-miR-424 | SELENBP1 |
| hsa-miR-424 | SERPINB9 |
| hsa-miR-424 | SETD3 |
| hsa-miR-424 | SFTPB |
| hsa-miR-424 | SIDT2 |
| hsa-miR-424 | SLC12A1 |
| hsa-miR-424 | SLC13A3 |
| hsa-miR-424 | SLC13A5 |
| hsa-miR-424 | SLC22A2 |
| hsa-miR-424 | SLC25A12 |
| hsa-miR-424 | SLC28A1 |
| hsa-miR-424 | SLC2A6 |
| hsa-miR-424 | SLC39A1 |
| hsa-miR-424 | SLC44A3 |
| hsa-miR-424 | SLC6A4 |
| hsa-miR-424 | SLCO3A1 |
| hsa-miR-424 | SNRK |
| hsa-miR-424 | SNURF |
| hsa-miR-424 | SOAT2 |
| hsa-miR-424 | SPEG |
| hsa-miR-424 | SPTBN2 |
| hsa-miR-424 | SRPX |
| hsa-miR-424 | SSH3 |
| hsa-miR-424 | STK32B |
| hsa-miR-424 | STX17 |
| hsa-miR-424 | STX1A |
| hsa-miR-424 | STXBP2 |
| hsa-miR-424 | SVIL |
| hsa-miR-424 | SYDE1 |
| hsa-miR-424 | SYNE1 |
| hsa-miR-424 | TACC2 |
| hsa-miR-424 | TARBP1 |
| hsa-miR-424 | TMEM130 |
| hsa-miR-424 | TMEM143 |
| hsa-miR-424 | TMEM14A |
| hsa-miR-424 | TMEM26 |
| hsa-miR-424 | TMEM79 |
| hsa-miR-424 | TMPRSS4 |
| hsa-miR-424 | TPM3 |
| hsa-miR-424 | TPST2 |
| hsa-miR-424 | TSPAN5 |
| hsa-miR-424 | TTC13 |
| hsa-miR-424 | TYRO3 |
| hsa-miR-424 | UGT8 |
| hsa-miR-424 | UNC5A |
| hsa-miR-424 | WDR47 |
| hsa-miR-424 | WDR7 |
| hsa-miR-424 | WWC1 |
| hsa-miR-424 | ZFAND5 |
| hsa-miR-424 | ZIK1 |
| hsa-miR-424 | ZNF496 |
| hsa-miR-429 | ABAT |
| hsa-miR-429 | ABCC9 |
| hsa-miR-429 | ACAT1 |
| hsa-miR-429 | ACVR1C |
| hsa-miR-429 | ADAM10 |
| hsa-miR-429 | ADCY2 |
| hsa-miR-429 | ADIPOR2 |
| hsa-miR-429 | ANGPTL3 |
| hsa-miR-429 | ANKRD28 |
| hsa-miR-429 | ANLN |
| hsa-miR-429 | ANO5 |
| hsa-miR-429 | ARHGAP20 |
| hsa-miR-429 | ARHGEF1 |
| hsa-miR-429 | ARHGEF17 |
| hsa-miR-429 | ARHGEF3 |
| hsa-miR-429 | ARID4A |
| hsa-miR-429 | ARL6IP6 |
| hsa-miR-429 | ASAP1 |
| hsa-miR-429 | ASH1L |
| hsa-miR-429 | ASXL3 |
| hsa-miR-429 | ATL2 |
| hsa-miR-429 | ATP11C |
| hsa-miR-429 | ATP1B1 |
| hsa-miR-429 | AUTS2 |
| hsa-miR-429 | BCL9 |
| hsa-miR-429 | BHLHE41 |
| hsa-miR-429 | C6orf120 |
| hsa-miR-429 | CACNA1C |
| hsa-miR-429 | CALHM1 |
| hsa-miR-429 | CASC1 |
| hsa-miR-429 | CASZ1 |
| hsa-miR-429 | CCNA2 |
| hsa-miR-429 | CCNE2 |
| hsa-miR-429 | CCNJL |
| hsa-miR-429 | CD58 |
| hsa-miR-429 | CDH11 |
| hsa-miR-429 | CDH6 |
| hsa-miR-429 | CDK16 |
| hsa-miR-429 | CDR2L |
| hsa-miR-429 | CEBPD |
| hsa-miR-429 | CECR2 |
| hsa-miR-429 | CEP41 |
| hsa-miR-429 | CFL2 |
| hsa-miR-429 | CHN2 |
| hsa-miR-429 | CITED2 |
| hsa-miR-429 | CLIP2 |
| hsa-miR-429 | CNOT6 |
| hsa-miR-429 | CNTFR |
| hsa-miR-429 | COBLL1 |
| hsa-miR-429 | COL4A3 |
| hsa-miR-429 | CREB5 |
| hsa-miR-429 | CTBP2 |
| hsa-miR-429 | CTNND2 |
| hsa-miR-429 | CYTH3 |
| hsa-miR-429 | DACH1 |
| hsa-miR-429 | DCDC2 |
| hsa-miR-429 | DENND5B |
| hsa-miR-429 | DGKA |
| hsa-miR-429 | DGKH |
| hsa-miR-429 | DIRAS2 |
| hsa-miR-429 | DLC1 |
| hsa-miR-429 | DLGAP2 |
| hsa-miR-429 | DMD |
| hsa-miR-429 | DMRT2 |
| hsa-miR-429 | DNAH5 |
| hsa-miR-429 | DNAJB9 |
| hsa-miR-429 | DNAJC10 |
| hsa-miR-429 | DNAJC5 |
| hsa-miR-429 | DNMT3A |
| hsa-miR-429 | DNMT3B |
| hsa-miR-429 | DOCK4 |
| hsa-miR-429 | DTNA |
| hsa-miR-429 | DUSP1 |
| hsa-miR-429 | DYRK2 |
| hsa-miR-429 | DZIP1 |
| hsa-miR-429 | E2F3 |
| hsa-miR-429 | ELAVL2 |
| hsa-miR-429 | ELL2 |
| hsa-miR-429 | ENDOD1 |
| hsa-miR-429 | ERBB4 |
| hsa-miR-429 | ERLIN1 |
| hsa-miR-429 | ERRFI1 |
| hsa-miR-429 | ESRP1 |
| hsa-miR-429 | ETS2 |
| hsa-miR-429 | EVI5 |
| hsa-miR-429 | FAM19A5 |
| hsa-miR-429 | FAM46C |
| hsa-miR-429 | FAM49B |
| hsa-miR-429 | FAM60A |
| hsa-miR-429 | FAM81A |
| hsa-miR-429 | FAM8A1 |
| hsa-miR-429 | FERMT2 |
| hsa-miR-429 | FGD1 |
| hsa-miR-429 | FHOD1 |
| hsa-miR-429 | FIGN |
| hsa-miR-429 | FOXF2 |
| hsa-miR-429 | FRMD4A |
| hsa-miR-429 | FRMD4B |
| hsa-miR-429 | FSCN1 |
| hsa-miR-429 | FUBP1 |
| hsa-miR-429 | FUT4 |
| hsa-miR-429 | FYN |
| hsa-miR-429 | G6PC |
| hsa-miR-429 | GABBR2 |
| hsa-miR-429 | GAL3ST1 |
| hsa-miR-429 | GALNT2 |
| hsa-miR-429 | GATA2 |
| hsa-miR-429 | GDA |
| hsa-miR-429 | GJC1 |
| hsa-miR-429 | GLI3 |
| hsa-miR-429 | GLIPR2 |
| hsa-miR-429 | GLIS2 |
| hsa-miR-429 | GOT1 |
| hsa-miR-429 | GPM6A |
| hsa-miR-429 | GPR107 |
| hsa-miR-429 | GPR146 |
| hsa-miR-429 | GRIP1 |
| hsa-miR-429 | HAL |
| hsa-miR-429 | HCFC2 |
| hsa-miR-429 | HLF |
| hsa-miR-429 | HNF1B |
| hsa-miR-429 | HS3ST1 |
| hsa-miR-429 | HS6ST2 |
| hsa-miR-429 | HSPB8 |
| hsa-miR-429 | IFNGR2 |
| hsa-miR-429 | IGSF3 |
| hsa-miR-429 | IL6ST |
| hsa-miR-429 | IRS1 |
| hsa-miR-429 | ITSN1 |
| hsa-miR-429 | JAG1 |
| hsa-miR-429 | JUN |
| hsa-miR-429 | KANK1 |
| hsa-miR-429 | KCND2 |
| hsa-miR-429 | KDR |
| hsa-miR-429 | KLF10 |
| hsa-miR-429 | KLF9 |
| hsa-miR-429 | KRT80 |
| hsa-miR-429 | LAMC1 |
| hsa-miR-429 | LARP1B |
| hsa-miR-429 | LCA5 |
| hsa-miR-429 | LEPROTL1 |
| hsa-miR-429 | LFNG |
| hsa-miR-429 | LMAN1 |
| hsa-miR-429 | LOX |
| hsa-miR-429 | LPIN1 |
| hsa-miR-429 | LRIG1 |
| hsa-miR-429 | LRP1 |
| hsa-miR-429 | MAP1B |
| hsa-miR-429 | MAP2 |
| hsa-miR-429 | MAP3K1 |
| hsa-miR-429 | MAP3K5 |
| hsa-miR-429 | MAP4K3 |
| hsa-miR-429 | MAPRE1 |
| hsa-miR-429 | MBLAC2 |
| hsa-miR-429 | MBOAT2 |
| hsa-miR-429 | MCC |
| hsa-miR-429 | MEX3B |
| hsa-miR-429 | MEX3D |
| hsa-miR-429 | MGAT2 |
| hsa-miR-429 | MLLT3 |
| hsa-miR-429 | MMAA |
| hsa-miR-429 | MMD |
| hsa-miR-429 | MMP16 |
| hsa-miR-429 | MOCS1 |
| hsa-miR-429 | MTFR1 |
| hsa-miR-429 | MTSS1L |
| hsa-miR-429 | MXD3 |
| hsa-miR-429 | MYB |
| hsa-miR-429 | MYLK |
| hsa-miR-429 | MYT1 |
| hsa-miR-429 | NANOS1 |
| hsa-miR-429 | NCOR2 |
| hsa-miR-429 | NCS1 |
| hsa-miR-429 | NDN |
| hsa-miR-429 | NEO1 |
| hsa-miR-429 | NFIA |
| hsa-miR-429 | NLGN4X |
| hsa-miR-429 | NOVA1 |
| hsa-miR-429 | NPNT |
| hsa-miR-429 | NPTX1 |
| hsa-miR-429 | NRBF2 |
| hsa-miR-429 | NRG1 |
| hsa-miR-429 | NRIP1 |
| hsa-miR-429 | NTF3 |
| hsa-miR-429 | OR51E1 |
| hsa-miR-429 | ORMDL3 |
| hsa-miR-429 | OSBPL11 |
| hsa-miR-429 | PAK6 |
| hsa-miR-429 | PALM2 |
| hsa-miR-429 | PALM2-AKAP2 |
| hsa-miR-429 | PAM |
| hsa-miR-429 | PAQR5 |
| hsa-miR-429 | PARD6B |
| hsa-miR-429 | PHF21B |
| hsa-miR-429 | PHLDB1 |
| hsa-miR-429 | PI4K2B |
| hsa-miR-429 | PITPNM3 |
| hsa-miR-429 | PKIA |
| hsa-miR-429 | PKP1 |
| hsa-miR-429 | PMAIP1 |
| hsa-miR-429 | POGZ |
| hsa-miR-429 | PPP2R1B |
| hsa-miR-429 | PPP2R2C |
| hsa-miR-429 | PPP4R2 |
| hsa-miR-429 | PRDM16 |
| hsa-miR-429 | PRKAG2 |
| hsa-miR-429 | PRKCA |
| hsa-miR-429 | PSAT1 |
| hsa-miR-429 | PTHLH |
| hsa-miR-429 | PTP4A1 |
| hsa-miR-429 | PTPN13 |
| hsa-miR-429 | RAB37 |
| hsa-miR-429 | RAP2C |
| hsa-miR-429 | RAPGEF2 |
| hsa-miR-429 | RASSF8 |
| hsa-miR-429 | RELN |
| hsa-miR-429 | RIMKLB |
| hsa-miR-429 | RIMS2 |
| hsa-miR-429 | RND3 |
| hsa-miR-429 | RPL22L1 |
| hsa-miR-429 | RUFY2 |
| hsa-miR-429 | SBF1 |
| hsa-miR-429 | SCD |
| hsa-miR-429 | SCML1 |
| hsa-miR-429 | SCN3B |
| hsa-miR-429 | SDC2 |
| hsa-miR-429 | SEC23A |
| hsa-miR-429 | SEC24A |
| hsa-miR-429 | SEC24D |
| hsa-miR-429 | SEC61A2 |
| hsa-miR-429 | SERINC1 |
| hsa-miR-429 | SESN1 |
| hsa-miR-429 | SFXN1 |
| hsa-miR-429 | SGIP1 |
| hsa-miR-429 | SHCBP1 |
| hsa-miR-429 | SLAIN1 |
| hsa-miR-429 | SLC14A1 |
| hsa-miR-429 | SLC16A2 |
| hsa-miR-429 | SLC17A4 |
| hsa-miR-429 | SLC23A2 |
| hsa-miR-429 | SLC30A10 |
| hsa-miR-429 | SLC35F2 |
| hsa-miR-429 | SLC38A2 |
| hsa-miR-429 | SLC38A4 |
| hsa-miR-429 | SLC39A14 |
| hsa-miR-429 | SLC6A1 |
| hsa-miR-429 | SMARCD1 |
| hsa-miR-429 | SMURF2 |
| hsa-miR-429 | SNAP25 |
| hsa-miR-429 | SOCS6 |
| hsa-miR-429 | SPRYD4 |
| hsa-miR-429 | SRGAP1 |
| hsa-miR-429 | ST6GALNAC5 |
| hsa-miR-429 | STYX |
| hsa-miR-429 | SULF1 |
| hsa-miR-429 | SWAP70 |
| hsa-miR-429 | SYDE1 |
| hsa-miR-429 | SYTL5 |
| hsa-miR-429 | SYVN1 |
| hsa-miR-429 | TBC1D22B |
| hsa-miR-429 | TFAP2A |
| hsa-miR-429 | TIMP2 |
| hsa-miR-429 | TLL2 |
| hsa-miR-429 | TMEM100 |
| hsa-miR-429 | TMEM135 |
| hsa-miR-429 | TMEM136 |
| hsa-miR-429 | TMEM14A |
| hsa-miR-429 | TMEM164 |
| hsa-miR-429 | TMEM170B |
| hsa-miR-429 | TMEM229B |
| hsa-miR-429 | TMEM57 |
| hsa-miR-429 | TOB1 |
| hsa-miR-429 | TP53INP1 |
| hsa-miR-429 | TRIO |
| hsa-miR-429 | TSC22D2 |
| hsa-miR-429 | TUBB3 |
| hsa-miR-429 | TUFT1 |
| hsa-miR-429 | UNC119B |
| hsa-miR-429 | USH1G |
| hsa-miR-429 | USH2A |
| hsa-miR-429 | UXS1 |
| hsa-miR-429 | VASH2 |
| hsa-miR-429 | VAT1L |
| hsa-miR-429 | VLDLR |
| hsa-miR-429 | WDR91 |
| hsa-miR-429 | WIPF3 |
| hsa-miR-429 | WWC3 |
| hsa-miR-429 | XKR6 |
| hsa-miR-429 | XKR8 |
| hsa-miR-429 | ZCCHC24 |
| hsa-miR-429 | ZFHX4 |
| hsa-miR-429 | ZNF292 |
| hsa-miR-429 | ZNF365 |
| hsa-miR-429 | ZNF532 |
| hsa-miR-429 | ZNF711 |
| hsa-miR-429 | ZSWIM4 |
| hsa-miR-429 | ZYG11B |
| hsa-miR-454 | ABCC9 |
| hsa-miR-454 | ACSL1 |
| hsa-miR-454 | AKAP1 |
| hsa-miR-454 | ATP11A |
| hsa-miR-454 | B4GALNT1 |
| hsa-miR-454 | C16orf70 |
| hsa-miR-454 | C1S |
| hsa-miR-454 | CDS1 |
| hsa-miR-454 | CIT |
| hsa-miR-454 | CPEB1 |
| hsa-miR-454 | CPEB3 |
| hsa-miR-454 | CRLS1 |
| hsa-miR-454 | CTSE |
| hsa-miR-454 | DICER1 |
| hsa-miR-454 | DLC1 |
| hsa-miR-454 | DLG5 |
| hsa-miR-454 | EDA2R |
| hsa-miR-454 | EPB41L5 |
| hsa-miR-454 | F3 |
| hsa-miR-454 | GLYATL1 |
| hsa-miR-454 | GPT2 |
| hsa-miR-454 | HIF1A |
| hsa-miR-454 | INPP5F |
| hsa-miR-454 | INTS6 |
| hsa-miR-454 | IQCK |
| hsa-miR-454 | ITIH5 |
| hsa-miR-454 | KALRN |
| hsa-miR-454 | KLHDC8A |
| hsa-miR-454 | LRP12 |
| hsa-miR-454 | MAP2 |
| hsa-miR-454 | MGST1 |
| hsa-miR-454 | NRSN1 |
| hsa-miR-454 | NUDT8 |
| hsa-miR-454 | PACS1 |
| hsa-miR-454 | PANK3 |
| hsa-miR-454 | PDLIM2 |
| hsa-miR-454 | PEX5L |
| hsa-miR-454 | PFKFB2 |
| hsa-miR-454 | PKHD1 |
| hsa-miR-454 | PPARG |
| hsa-miR-454 | PPP2R1B |
| hsa-miR-454 | PUS7 |
| hsa-miR-454 | RAB34 |
| hsa-miR-454 | RAD51 |
| hsa-miR-454 | RDH11 |
| hsa-miR-454 | RFWD3 |
| hsa-miR-454 | SACM1L |
| hsa-miR-454 | SAMD4A |
| hsa-miR-454 | SLAIN1 |
| hsa-miR-454 | SLC22A15 |
| hsa-miR-454 | SLC25A27 |
| hsa-miR-454 | SLCO2A1 |
| hsa-miR-454 | SOX5 |
| hsa-miR-454 | SRPX |
| hsa-miR-454 | ST3GAL6 |
| hsa-miR-454 | STAT3 |
| hsa-miR-454 | STK33 |
| hsa-miR-454 | SYT10 |
| hsa-miR-454 | SYT6 |
| hsa-miR-454 | TEK |
| hsa-miR-454 | THOP1 |
| hsa-miR-454 | WDR35 |
| hsa-miR-454 | WNT2B |
| hsa-miR-543 | CLPX |
| hsa-miR-543 | DGAT2 |
| hsa-miR-543 | DNAJC10 |
| hsa-miR-543 | LMO1 |
| hsa-miR-543 | MTHFD1L |
| hsa-miR-543 | PANK3 |
| hsa-miR-543 | SAMD4A |
| hsa-miR-543 | SNAP25 |
| hsa-miR-543 | SS18L1 |
| hsa-miR-543 | TCERG1 |
| hsa-miR-543 | ZFAND5 |
| hsa-miR-760 | ARNTL |
| hsa-miR-92b | AACS |
| hsa-miR-92b | ACAA1 |
| hsa-miR-92b | ACAD8 |
| hsa-miR-92b | ACAN |
| hsa-miR-92b | ADARB2 |
| hsa-miR-92b | ADM |
| hsa-miR-92b | ANKRD46 |
| hsa-miR-92b | ANTXR1 |
| hsa-miR-92b | ASTN1 |
| hsa-miR-92b | ATP13A4 |
| hsa-miR-92b | BPHL |
| hsa-miR-92b | C19orf12 |
| hsa-miR-92b | C1RL |
| hsa-miR-92b | C20orf194 |
| hsa-miR-92b | CCNJL |
| hsa-miR-92b | CD4 |
| hsa-miR-92b | CLCN5 |
| hsa-miR-92b | CNDP1 |
| hsa-miR-92b | CPEB3 |
| hsa-miR-92b | CREM |
| hsa-miR-92b | CSMD2 |
| hsa-miR-92b | CTTNBP2 |
| hsa-miR-92b | DCUN1D4 |
| hsa-miR-92b | DDO |
| hsa-miR-92b | DGKA |
| hsa-miR-92b | DKK3 |
| hsa-miR-92b | DLG5 |
| hsa-miR-92b | DOCK4 |
| hsa-miR-92b | DPPA4 |
| hsa-miR-92b | DTL |
| hsa-miR-92b | FAIM |
| hsa-miR-92b | FAM3B |
| hsa-miR-92b | FGF1 |
| hsa-miR-92b | FOSL2 |
| hsa-miR-92b | FZD6 |
| hsa-miR-92b | GATA2 |
| hsa-miR-92b | GATM |
| hsa-miR-92b | GOT1 |
| hsa-miR-92b | GPR180 |
| hsa-miR-92b | HECTD1 |
| hsa-miR-92b | HMGCR |
| hsa-miR-92b | IL1RL1 |
| hsa-miR-92b | ILF3 |
| hsa-miR-92b | ISG20 |
| hsa-miR-92b | ITGA5 |
| hsa-miR-92b | ITGAV |
| hsa-miR-92b | KCNC4 |
| hsa-miR-92b | KCTD2 |
| hsa-miR-92b | KCTD7 |
| hsa-miR-92b | KIAA0556 |
| hsa-miR-92b | KIAA1524 |
| hsa-miR-92b | KIAA1841 |
| hsa-miR-92b | KLRD1 |
| hsa-miR-92b | LARS2 |
| hsa-miR-92b | LGI2 |
| hsa-miR-92b | LIMK1 |
| hsa-miR-92b | MARK1 |
| hsa-miR-92b | MCOLN2 |
| hsa-miR-92b | MMD |
| hsa-miR-92b | MOCS2 |
| hsa-miR-92b | NEO1 |
| hsa-miR-92b | NF2 |
| hsa-miR-92b | NOX4 |
| hsa-miR-92b | NSMAF |
| hsa-miR-92b | OR51E1 |
| hsa-miR-92b | OSBPL5 |
| hsa-miR-92b | PCGF6 |
| hsa-miR-92b | PCSK6 |
| hsa-miR-92b | PCYT1B |
| hsa-miR-92b | PLCD3 |
| hsa-miR-92b | PPARA |
| hsa-miR-92b | PRKAG2 |
| hsa-miR-92b | PUS7 |
| hsa-miR-92b | RAP2C |
| hsa-miR-92b | RNF44 |
| hsa-miR-92b | RRBP1 |
| hsa-miR-92b | SDC2 |
| hsa-miR-92b | SH3BP5 |
| hsa-miR-92b | SHMT1 |
| hsa-miR-92b | SIM2 |
| hsa-miR-92b | SIRT6 |
| hsa-miR-92b | SLC12A2 |
| hsa-miR-92b | SLC25A16 |
| hsa-miR-92b | SLC25A36 |
| hsa-miR-92b | SLC4A10 |
| hsa-miR-92b | SLC9A1 |
| hsa-miR-92b | SLC9A3R2 |
| hsa-miR-92b | SLFN5 |
| hsa-miR-92b | SOCS2 |
| hsa-miR-92b | SORBS3 |
| hsa-miR-92b | SP6 |
| hsa-miR-92b | SPATA18 |
| hsa-miR-92b | SPATS2 |
| hsa-miR-92b | SSFA2 |
| hsa-miR-92b | STXBP4 |
| hsa-miR-92b | TACC2 |
| hsa-miR-92b | TBX3 |
| hsa-miR-92b | TEK |
| hsa-miR-92b | TMEM53 |
| hsa-miR-92b | TRIM6 |
| hsa-miR-92b | TRIO |
| hsa-miR-92b | VASH2 |
| hsa-miR-92b | ZNF605 |
| hsa-miR-96 | ACACB |
| hsa-miR-96 | ADCY6 |
| hsa-miR-96 | AKAP1 |
| hsa-miR-96 | ARHGEF10L |
| hsa-miR-96 | ARID4A |
| hsa-miR-96 | ARL4D |
| hsa-miR-96 | ATP1B3 |
| hsa-miR-96 | B4GALNT1 |
| hsa-miR-96 | BDKRB2 |
| hsa-miR-96 | BTBD3 |
| hsa-miR-96 | CCM2 |
| hsa-miR-96 | CD36 |
| hsa-miR-96 | CEP170 |
| hsa-miR-96 | CEP250 |
| hsa-miR-96 | CHMP4A |
| hsa-miR-96 | CHST10 |
| hsa-miR-96 | CMTM7 |
| hsa-miR-96 | CNKSR1 |
| hsa-miR-96 | CPEB1 |
| hsa-miR-96 | CTSB |
| hsa-miR-96 | CTTN |
| hsa-miR-96 | DGKH |
| hsa-miR-96 | DHCR24 |
| hsa-miR-96 | DICER1 |
| hsa-miR-96 | DPYD |
| hsa-miR-96 | EBF3 |
| hsa-miR-96 | ECT2 |
| hsa-miR-96 | EEFSEC |
| hsa-miR-96 | ENO3 |
| hsa-miR-96 | EPHA7 |
| hsa-miR-96 | ETS2 |
| hsa-miR-96 | FAM57A |
| hsa-miR-96 | FAM81A |
| hsa-miR-96 | FHL1 |
| hsa-miR-96 | FNDC3A |
| hsa-miR-96 | GAD2 |
| hsa-miR-96 | GNE |
| hsa-miR-96 | GPHN |
| hsa-miR-96 | GPR180 |
| hsa-miR-96 | GPRC5C |
| hsa-miR-96 | GRAMD1C |
| hsa-miR-96 | HDAC9 |
| hsa-miR-96 | HMGB2 |
| hsa-miR-96 | HS6ST2 |
| hsa-miR-96 | ITGA6 |
| hsa-miR-96 | KCNG1 |
| hsa-miR-96 | KCNK2 |
| hsa-miR-96 | KIAA1841 |
| hsa-miR-96 | LGR6 |
| hsa-miR-96 | LOXL4 |
| hsa-miR-96 | LRRC3 |
| hsa-miR-96 | MAP2K3 |
| hsa-miR-96 | MARVELD1 |
| hsa-miR-96 | MASP1 |
| hsa-miR-96 | MAT1A |
| hsa-miR-96 | MCCC2 |
| hsa-miR-96 | MCM4 |
| hsa-miR-96 | MOGAT2 |
| hsa-miR-96 | MT1X |
| hsa-miR-96 | MTA3 |
| hsa-miR-96 | MTM1 |
| hsa-miR-96 | MYRIP |
| hsa-miR-96 | NEDD4 |
| hsa-miR-96 | NIPA1 |
| hsa-miR-96 | NMNAT1 |
| hsa-miR-96 | NOD1 |
| hsa-miR-96 | NOVA1 |
| hsa-miR-96 | NRG1 |
| hsa-miR-96 | OLFML1 |
| hsa-miR-96 | OSBPL10 |
| hsa-miR-96 | PAQR8 |
| hsa-miR-96 | PCCB |
| hsa-miR-96 | PFKFB2 |
| hsa-miR-96 | PFKFB3 |
| hsa-miR-96 | PLCH1 |
| hsa-miR-96 | PPFIA4 |
| hsa-miR-96 | PRCP |
| hsa-miR-96 | PTPRB |
| hsa-miR-96 | RAB34 |
| hsa-miR-96 | RAC2 |
| hsa-miR-96 | RAPGEF2 |
| hsa-miR-96 | RIC8B |
| hsa-miR-96 | RIMS2 |
| hsa-miR-96 | RNASE7 |
| hsa-miR-96 | RNF152 |
| hsa-miR-96 | SALL2 |
| hsa-miR-96 | SDC2 |
| hsa-miR-96 | SERPINA5 |
| hsa-miR-96 | SHC1 |
| hsa-miR-96 | SLC25A1 |
| hsa-miR-96 | SLC25A16 |
| hsa-miR-96 | SLC6A13 |
| hsa-miR-96 | SLCO3A1 |
| hsa-miR-96 | SNURF |
| hsa-miR-96 | SPATA20 |
| hsa-miR-96 | STK33 |
| hsa-miR-96 | SYT9 |
| hsa-miR-96 | TBC1D22B |
| hsa-miR-96 | TBX15 |
| hsa-miR-96 | TEAD4 |
| hsa-miR-96 | THSD4 |
| hsa-miR-96 | TINAG |
| hsa-miR-96 | TPM2 |
| hsa-miR-96 | TRMT11 |
| hsa-miR-96 | TYRO3 |
| hsa-miR-96 | ZFAND5 |
| hsa-miR-96 | ZFP36L1 |
| hsa-miR-96 | ZHX1 |
| hsa-miR-96 | ZNF365 |
| hsa-miR-96 | ZNF496 |
| hsa-miR-96 | ZNHIT1 |
| hsa-miR-99a | ACADVL |
| hsa-miR-99a | ANKRD28 |
| hsa-miR-99a | ENPP1 |
| hsa-miR-99a | GPRC5B |
| hsa-miR-99a | IMPDH1 |
| hsa-miR-99a | LAMA5 |
| hsa-miR-99a | LRP1 |
| hsa-miR-99a | PODXL |
| hsa-miR-99a | SLC14A1 |
| hsa-miR-99a | ST6GALNAC4 |
| hsa-miR-99a | VNN1 |
| hsa-miR-99a | ZNF19 |
| hsa-miR-99b | ACADVL |
| hsa-miR-99b | ANKRD28 |
| hsa-miR-99b | CPNE5 |
| hsa-miR-99b | ENPP1 |
| hsa-miR-99b | GPRC5B |
| hsa-miR-99b | IMPDH1 |
| hsa-miR-99b | LAMA5 |
| hsa-miR-99b | NLRP2 |
| hsa-miR-99b | SLC14A1 |
| hsa-miR-99b | ST6GALNAC4 |
| hsa-miR-99b | VNN1 |
| hsa-miR-99b | ZNF19 |

| **Table S3. Details of upregulated and downregulated miRNAs in ceRNA network.** | | | | |
| --- | --- | --- | --- | --- |
| miRNA | log2FC | P-value | FDR | Up or down regulated |
| hsa-miR-183 | 5.236291 | 3.34E-28 | 1.85E-25 | up |
| hsa-miR-182 | 4.858979 | 1.56E-27 | 4.33E-25 | up |
| hsa-miR-96 | 5.632485 | 4.87E-21 | 6.74E-19 | up |
| hsa-miR-21 | 2.746804 | 7.53E-16 | 2.61E-14 | up |
| hsa-miR-27a | 2.186178 | 5.95E-13 | 1.50E-11 | up |
| hsa-miR-92b | 3.082046 | 1.79E-10 | 3.81E-09 | up |
| hsa-miR-23a | 1.71952 | 2.46E-10 | 5.04E-09 | up |
| hsa-miR-200a | 2.664065 | 6.20E-10 | 1.19E-08 | up |
| hsa-miR-181d | 2.853487 | 1.94E-09 | 3.26E-08 | up |
| hsa-miR-383 | -2.9442 | 2.17E-09 | 3.54E-08 | down |
| hsa-miR-122 | -4.18617 | 4.09E-09 | 6.29E-08 | down |
| hsa-miR-429 | 2.696647 | 9.93E-09 | 1.45E-07 | up |
| hsa-miR-222 | 2.120086 | 1.87E-08 | 2.59E-07 | up |
| hsa-miR-221 | 1.959898 | 6.03E-08 | 7.42E-07 | up |
| hsa-miR-22 | -1.23312 | 6.53E-08 | 7.75E-07 | down |
| hsa-miR-152 | -1.21855 | 7.96E-08 | 9.04E-07 | down |
| hsa-miR-181c | 2.108285 | 9.35E-08 | 1.04E-06 | up |
| hsa-miR-454 | 1.727235 | 3.56E-07 | 3.79E-06 | up |
| hsa-miR-135b | 5.097718 | 4.54E-07 | 4.74E-06 | up |
| hsa-miR-424 | -1.37941 | 1.35E-06 | 1.29E-05 | down |
| hsa-miR-301a | 1.354072 | 1.33E-05 | 0.000111 | up |
| hsa-miR-200c | 4.743502 | 0.000103 | 0.000693 | up |
| hsa-miR-196b | 3.947407 | 0.000105 | 0.0007 | up |
| hsa-miR-33b | -1.48591 | 0.000117 | 0.000754 | down |
| hsa-miR-141 | 4.46886 | 0.000167 | 0.001039 | up |
| hsa-miR-187 | 2.759895 | 0.000627 | 0.003309 | up |
| hsa-miR-211 | -2.1953 | 0.001355 | 0.006418 | down |
| hsa-miR-31 | 4.349912 | 0.003019 | 0.012121 | up |
| hsa-miR-137 | 5.021968 | 0.003384 | 0.013019 | up |
| hsa-miR-205 | 5.980153 | 0.006533 | 0.022341 | up |

| **Table S4. Details of upregulated and downregulated lncRNAs in ceRNA network.** | | | | |
| --- | --- | --- | --- | --- |
| lncRNA | log2FC | P-value | FDR | Up or down regulated |
| U91324.1 | -5.85198 | 2.23E-16 | 1.73E-14 | down |
| FAM99B | -5.80796 | 2.29E-10 | 6.12E-09 | down |
| AC104809.2 | -4.06251 | 3.52E-09 | 7.41E-08 | down |
| HULC | -4.37183 | 2.36E-08 | 4.04E-07 | down |
| FTCD-AS1 | -3.18139 | 5.53E-06 | 4.86E-05 | down |
| ARHGEF26-AS1 | -1.88314 | 1.10E-05 | 8.68E-05 | down |
| TM4SF19-AS1 | 1.823411 | 0.000769 | 0.003054 | up |
| AC022182.1 | 3.615764 | 0.002058 | 0.006916 | up |
| MIAT | 2.03911 | 0.003795 | 0.01152 | up |
| AC112721.2 | 4.390708 | 0.004139 | 0.012366 | up |
| LINC00402 | -2.35256 | 0.004617 | 0.013535 | down |
| LINC00501 | 3.600156 | 0.007082 | 0.019267 | up |
| AC092296.1 | -1.61737 | 0.011154 | 0.027943 | down |
| HMGA1P4 | 1.508017 | 0.013311 | 0.032315 | up |
| LINC00517 | 2.690748 | 0.015259 | 0.03625 | up |
| AC007386.2 | 2.603391 | 0.016422 | 0.03843 | up |

| **Table S5. Details of upregulated and downregulated mRNAs in ceRNA network.** | | | | |
| --- | --- | --- | --- | --- |
| mRNA | log2FC | P-value | FDR | Up or down regulated |
| SHMT1 | -4.13974 | 3.48E-31 | 5.38E-29 | down |
| CNDP1 | -6.2096 | 1.70E-22 | 9.77E-21 | down |
| ABCC9 | -4.37841 | 6.61E-21 | 3.19E-19 | down |
| STAB2 | -4.18274 | 2.17E-15 | 5.27E-14 | down |
| DPYS | -6.42478 | 1.19E-14 | 2.61E-13 | down |
| ANGPTL3 | -5.76878 | 8.45E-14 | 1.63E-12 | down |
| CES2 | -3.17562 | 2.79E-13 | 4.94E-12 | down |
| GPLD1 | -3.90657 | 2.04E-12 | 3.19E-11 | down |
| AHSG | -6.25504 | 3.65E-12 | 5.46E-11 | down |
| NAGS | -3.74007 | 5.19E-12 | 7.61E-11 | down |
| CD4 | -2.26044 | 2.67E-11 | 3.51E-10 | down |
| UGT3A1 | -5.42476 | 3.27E-11 | 4.22E-10 | down |
| PLA2G12B | -5.26539 | 1.50E-10 | 1.75E-09 | down |
| CLEC4M | -5.6848 | 8.94E-10 | 9.18E-09 | down |
| CCDC25 | -1.33135 | 3.60E-09 | 3.35E-08 | down |
| KLF9 | -2.07083 | 1.11E-08 | 9.58E-08 | down |
| HFE2 | -5.19823 | 2.99E-08 | 2.39E-07 | down |
| CD1D | -2.43416 | 2.80E-07 | 1.90E-06 | down |
| AP1G2 | 2.417316 | 9.23E-07 | 5.61E-06 | up |
| SLC22A9 | -3.72288 | 1.65E-06 | 9.54E-06 | down |
| GNAO1 | -2.85055 | 4.99E-06 | 2.61E-05 | down |
| TMEM52 | -2.12707 | 6.13E-06 | 3.16E-05 | down |
| SCN3B | 3.323541 | 7.44E-06 | 3.77E-05 | up |
| TF | -4.20629 | 1.25E-05 | 6.01E-05 | down |
| HSH2D | 2.687355 | 1.52E-05 | 7.14E-05 | up |
| MCC | -2.06848 | 2.17E-05 | 9.82E-05 | down |
| LDLRAP1 | -1.34048 | 4.99E-05 | 0.000207 | down |
| NKAIN1 | 5.595859 | 5.51E-05 | 0.000227 | up |
| CSMD2 | 4.433466 | 5.67E-05 | 0.000233 | up |
| DENND3 | 1.302999 | 0.000112 | 0.000426 | up |
| TIAM1 | -1.48105 | 0.000135 | 0.000503 | down |
| PRKG2 | 5.186772 | 0.000218 | 0.000771 | up |
| OPRL1 | 1.643363 | 0.00023 | 0.000808 | up |
| FCAMR | -2.99078 | 0.000237 | 0.00083 | down |
| RAC2 | 1.93685 | 0.000355 | 0.001184 | up |
| TNFRSF1B | -1.29218 | 0.000441 | 0.001429 | down |
| PPM1K | -1.43978 | 0.000482 | 0.001546 | down |
| SLC6A2 | -3.40814 | 0.000642 | 0.001995 | down |
| HCFC2 | -1.04581 | 0.000703 | 0.002168 | down |
| SEMA4D | 1.416061 | 0.000752 | 0.002302 | up |
| FAM117A | -1.12379 | 0.000954 | 0.002838 | down |
| ARHGEF1 | 1.248299 | 0.001069 | 0.003137 | up |
| DLG2 | -1.72399 | 0.001121 | 0.003264 | down |
| PJA1 | 1.292343 | 0.001319 | 0.003768 | up |
| ASB13 | -1.11626 | 0.001359 | 0.003867 | down |
| SYP | 2.454361 | 0.001448 | 0.004093 | up |
| SLC31A2 | -1.55755 | 0.001522 | 0.004277 | down |
| JAK3 | 1.83144 | 0.001539 | 0.004315 | up |
| SLC20A1 | -1.01663 | 0.001682 | 0.004672 | down |
| CDH20 | 5.783934 | 0.001721 | 0.004762 | up |
| BBS12 | 1.585067 | 0.002226 | 0.00595 | up |
| PEX5L | 3.222058 | 0.002237 | 0.005977 | up |
| DGKA | 2.005489 | 0.002285 | 0.006083 | up |
| KLRF1 | -1.71996 | 0.002343 | 0.006222 | down |
| KLRD1 | -1.44772 | 0.002534 | 0.006677 | down |
| GPR132 | 1.813448 | 0.002925 | 0.007563 | up |
| SERPINB9 | -1.06772 | 0.002971 | 0.007666 | down |
| RAB37 | -1.18274 | 0.003163 | 0.008105 | down |
| CCDC42 | -1.858 | 0.003175 | 0.008133 | down |
| NFATC1 | 1.120969 | 0.00323 | 0.008256 | up |
| ERBB4 | 2.805099 | 0.003466 | 0.008762 | up |
| PROCR | 2.408152 | 0.004717 | 0.011458 | up |
| CACNA1C | 1.530414 | 0.006026 | 0.014154 | up |
| ZNF589 | 1.408236 | 0.006811 | 0.01574 | up |
| MCOLN2 | 2.13974 | 0.007169 | 0.016464 | up |
| CCR7 | 3.16893 | 0.008474 | 0.018997 | up |
| ASTN1 | -1.56899 | 0.00853 | 0.019094 | down |
| CYP4B1 | 3.178786 | 0.009092 | 0.020173 | up |
| DOK3 | 1.472823 | 0.009738 | 0.021453 | up |
| TKTL1 | -1.66805 | 0.010437 | 0.022759 | down |
| IL21R | 1.869044 | 0.010769 | 0.023379 | up |
| RGS10 | 1.289135 | 0.010897 | 0.023615 | up |
| GPM6B | 1.472636 | 0.012794 | 0.027171 | up |
| MS4A1 | 4.360733 | 0.013225 | 0.027936 | up |
| SRPX | -1.33337 | 0.01436 | 0.030028 | down |
| TXNIP | -1.04843 | 0.019059 | 0.038252 | down |
| ST8SIA1 | 1.647046 | 0.021063 | 0.04174 | up |
| CD79B | 2.345123 | 0.022336 | 0.043928 | up |
| PCSK1 | 3.152916 | 0.022533 | 0.044265 | up |
| TCL1A | 3.969527 | 0.024464 | 0.047513 | up |

| **Table S6. GO pathway enrichment of mRNAs in ceRNA network.** | | | | | | |
| --- | --- | --- | --- | --- | --- | --- |
| Category | Term | | Count | % | P-Value | Genes |
| GOTERM_BP | | GO:0002250~adaptive immune response | 5 | 6.25 | 0.00413 | CD4, CD79B, JAK3, FCAMR, CLEC4M |
| GOTERM_BP | | GO:0045058~T cell selection | 2 | 2.5 | 0.01316 | CD4, CD1D |
| GOTERM_BP | | GO:0019233~sensory perception of pain | 3 | 3.75 | 0.02204 | OPRL1, SCN3B, DLG2 |
| GOTERM_BP | | GO:0042102~positive regulation of T cell proliferation | 3 | 3.75 | 0.02877 | CD4, JAK3, CD1D |
| GOTERM_BP | | GO:0006955~immune response | 6 | 7.5 | 0.03809 | SERPINB9, TNFRSF1B, CCR7, CD4, CD79B, SEMA4D |
| GOTERM_BP | | GO:0043547~positive regulation of GTPase activity | 7 | 8.75 | 0.03817 | RGS10, GNAO1, ARHGEF1, ERBB4, SEMA4D, JAK3, DENND3 |
| GOTERM_BP | | GO:0007167~enzyme linked receptor protein signaling pathway | 2 | 2.5 | 0.03898 | CD4, JAK3 |
| GOTERM_BP | | GO:0001503~ossification | 3 | 3.75 | 0.04856 | GPLD1, GPM6B, AHSG |
| GOTERM_CC | | GO:0005886~plasma membrane | 36 | 45 | ####### | HFE2, SLC20A1, ERBB4, SCN3B, SLC6A2, NKAIN1, HCFC2, GPM6B, CSMD2, DGKA, RGS10, CDH20, TNFRSF1B, RAC2, PROCR, TIAM1, CD4, MCOLN2, KLRF1, KLRD1, DLG2, GNAO1, ARHGEF1, KLF9, OPRL1, GPR132, STAB2, FCAMR, CD1D, ABCC9, CCR7, DOK3, CD79B, MCC, SEMA4D, CACNA1C |
| GOTERM_CC | | GO:0009897~external side of plasma membrane | 7 | 8.75 | ####### | CCR7, MS4A1, ASTN1, CD4, CD79B, STAB2, KLRD1 |
| GOTERM_CC | | GO:0005887~integral component of plasma membrane | 14 | 17.5 | 0.00675 | TNFRSF1B, SLC22A9, PROCR, SLC20A1, OPRL1, SLC6A2, MS4A1, CD79B, STAB2, SEMA4D, KLRF1, SLC31A2, CD1D, CLEC4M |
| GOTERM_CC | | GO:0009986~cell surface | 8 | 10 | 0.00848 | TF, CCR7, SRPX, HFE2, PROCR, SLC6A2, ANGPTL3, CD1D |
| GOTERM_CC | | GO:0030121~AP-1 adaptor complex | 2 | 2.5 | 0.02541 | AP1G2, LDLRAP1 |
| GOTERM_CC | | GO:1990712~HFE-transferrin receptor complex | 2 | 2.5 | 0.03374 | TF, HFE2 |
| GOTERM_CC | | GO:0016323~basolateral plasma membrane | 4 | 5 | 0.04206 | SLC22A9, ERBB4, DLG2, CD1D |
| GOTERM_CC | | GO:0034774~secretory granule lumen | 2 | 2.5 | 0.05018 | TF, PCSK1 |
| GOTERM_MF | | GO:0004872~receptor activity | 7 | 8.75 | ####### | SLC20A1, PROCR, CD4, MCC, SEMA4D, CD1D, CLEC4M |
| GOTERM_MF | | GO:0004888~transmembrane signaling receptor activity | 6 | 7.5 | 0.00233 | CD4, CD79B, SEMA4D, KLRF1, FCAMR, KLRD1 |
| GOTERM_MF | | GO:1990459~transferrin receptor binding | 2 | 2.5 | 0.03408 | TF, HFE2 |

| **Table S7. KEGG pathway enrichment of subnetworks in ceRNA network.** | | | | | | |
| --- | --- | --- | --- | --- | --- | --- |
| Category | Term | Count | % | P-Value | Genes | Subnetwork |
| KEGG_PATHWAY | hsa04062:Chemokine signaling pathway | 4 | 0.131148 | 0.004554 | CCR7, RAC2, TIAM1, JAK3 | 1 |
| KEGG_PATHWAY | hsa04662:B cell receptor signaling pathway | 3 | 0.098361 | 0.007202 | RAC2, CD79B, NFATC1 | 1 |
| KEGG_PATHWAY | hsa04640:Hematopoietic cell lineage | 3 | 0.098361 | 0.011266 | MS4A1, CD4, CD1D | 1 |
| KEGG_PATHWAY | hsa04650:Natural killer cell mediated cytotoxicity | 3 | 0.098361 | 0.021416 | RAC2, KLRD1, NFATC1 | 1 |
| KEGG_PATHWAY | hsa04024:cAMP signaling pathway | 3 | 0.098361 | 0.052201 | RAC2, TIAM1, NFATC1 | 1 |
| KEGG_PATHWAY | hsa04713:Circadian entrainment | 3 | 0.199203 | 0.001112 | GNAO1, PRKG2, CACNA1C | 2 |
| KEGG_PATHWAY | hsa04730:Long-term depression | 2 | 0.132802 | 0.034442 | GNAO1, PRKG2 | 2 |
| KEGG_PATHWAY | hsa04924:Renin secretion | 2 | 0.132802 | 0.036706 | PRKG2, CACNA1C | 2 |
| KEGG_PATHWAY | hsa04727:GABAergic synapse | 2 | 0.132802 | 0.048528 | GNAO1, CACNA1C | 2 |
| KEGG_PATHWAY | hsa01230:Biosynthesis of amino acids | 3 | 0.539568 | 3.22E-04 | SHMT1, NAGS, TKTL1 | 3 |
| KEGG_PATHWAY | hsa01100:Metabolic pathways | 4 | 0.719424 | 0.005553 | SHMT1, NAGS, CNDP1, TKTL1 | 3 |
| KEGG_PATHWAY | hsa01200:Carbon metabolism | 2 | 0.359712 | 0.048482 | SHMT1, TKTL1 | 3 |
